# Supplementary material for: Flexibility and sensitivity in gene regulation out of equilibrium
Source: Proc Natl Acad Sci U S A. 2024 Nov 5;121(46):e2411395121. doi: 10.1073/pnas.2411395121 (PMC11573582; doi:10.1073/pnas.2411395121)
Supplement: Supplementary file 1 — Appendix 01 (PDF) [file pnas.2411395121.sapp.pdf]

Supporting Information for

Flexibility and sensitivity in gene regulation out of equilibrium

S. D. Mahdavi*, G. L. Salmon*, P. Daghlian, H. G. Garcia, and R. Phillips (* contributed equally)

Gabriel Salmon
E-mail: gsalmon@caltech.edu
Rob Phillips
E-mail: phillips@pboc.caltech.edu

This PDF file includes:

Figs. S1 to S51
Tables S1 to S2
SI References

Contents

1 Linear Markovian dynamics, $\frac{d\mathbf{p}}{dt} = \mathbf{L}\mathbf{p}$, and cycles, are common **3**
A Mathematically, to first order, many dynamics are continuous time Markov chains . . . 3
B Closed steady-state systems are either equilibrium or cyclic . . . 3
B.1 Example of an acyclic system: the simple repression motif . . . 4
C The cycle condition relates a ratio of rate constants to (non)equilibrium . . . 5
D Discussion of various ways of quantifying dissipation . . . 6
E Other destinies of biochemical dissipation in cells beyond gene expression . . . 6

2 Insights into the square graph **7**
A The simple four-state cycle motif pervades prokaryotic and eukaryotic gene regulation . . . 7
A.1 Up-to-date census of known regulatory interactions in *E. coli* . . . 7
A.2 Up-to-date census of known regulatory interactions in *D. melanogaster* . . . 10
B Order of magnitude estimated rate constants for prokaryotic transcription . . . 11
C Biologically, timescales are plausibly separated enough that transcription is well represented by small Markov chains . . . 13
D Deriving the universal form: The Matrix Tree Theorem on the square graph yields a ratio of quadratic polynomials 15
E Discussion on observable conventions: the logarithmic control variable . . . 16
F Collapse of eight parameters into two emergent fundamental shape parameters (a, b) . . . 17
G Equilibrium responses of the square graph . . . 18
G.1 Demotion of responses to a (monotonic) ratio of linear polynomials at equilibrium . . . 18
G.2 Leakiness, saturation, and EC50 are tunable at equilibrium . . . 20
G.3 Validating consilience between kinetic and thermodynamic viewpoints . . . 22
G.4 Detailed balance is implied by $\gamma = 1$ and steady-state . . . 24
G.5 The cycle condition implies that changing transcription factor or polymerase concentrations does not affect the extent of disequilibrium in the square graph . . . 25
G.6 Connection and contrast with earlier results on kinetic cycles or square-graph analogs . . . 25
H Driving different arrows in the square graph can still yield a ratio of quadratic polynomials . . . 26
I Any averaged observable $\langle r \rangle$ has zero, one, two, or three inflection points, with varying monotonicity . . . 27
I.1 Descartes' rule of signs on second-derivative-polynomial with (a, b) reveals precise restrictions on numbers of inflections . . . 27
I.2 Monotonicity of response via (a, b) parameterization . . . 28
I.3 Bounds on the absolute magnitudes of response extrema . . . 29
I.4 Number of inflection points via the (a, b) parameterization . . . 30
J New bounds on nonequilibrium sensitivity . . . 32
J.1 Motivation of the the definition of the normalized sensitivity . . . 32
J.2 Connection to other measures of sensitivity and the effective Hill coefficient . . . 33
J.3 Summary of our results; contrast with existing bounds . . . 33
J.4 General upper bound on a related, differently-normalized slope . . . 34
J.5 General upper bound on our normalized sensitivity . . . 36
J.6 Symbolic derivation of bounds for triply-inflected outputs . . . 36
K Systematic census of effects of driving one or two edges . . . 37
K.1 Scaling a single rate constant at a time is identified with a proportional drive . . . 37
K.2 Quantitative comparisons of response curves with and without rate symmetries enforced . . . 37
L Crucial imbalances in rate-constants are required for nonmonotonic responses . . . 41
L.1 Minimum drive to reach nonmonotonic phenotypes . . . 43
L.2 Conditions that suffice to forbid nonmonotonicity . . . 43
M Implications of critical symmetry conditions for widespread numerical screens . . . 45
M.1 Enforced monotonocity of transcriptional responses in a decision theory model of nonequilibrium gene regulation . . . 45
M.2 Another recent nonequilibrium transcription model often remains monotonic in enhancer concentration 47

3 Discovering generalizations of response behaviors for more complex networks **48**
A Summary of nonequilibrium generalization of important transcriptional motifs . . . 48
B The hypercubic graph: regulation by N transcription factors . . . 50
B.1 Illustration with the 3−cube case . . . 51
B.2 Slicing the response function when one transcription factor is fixed . . . 57
B.3 Full generalization to the hypercube in n dimensions: cubes within cubes . . . 57
B.4 Further illustration with $n = 4$ tesseract graph . . . 59
B.5 Collapse of the output function at detailed balance . . . 60

B.6 Accommodating multiple binding sites for a transcription factor . . . 61
C Common graphs formed by subsets of the hypercube . . . 68
C.1 N strictly exclusive transcription factors: the book/rolodex graph . . . 68
C.2 General response function of the book graph from multiple exclusive transcription factors . . . 70
D All nontrivial, transcriptionally-potent subgraphs of the $n = 4$ hypercube . . . 71
E Connecting to successful nonequilibrium dynamic models of transcription-factor-driven chromatin accessibility . 74
F Analysis of larger regulatory networks involving DNA looping . . . 75
F.1 Repression . . . 75
F.2 Repression by DNA looping is subject to kinetic criteria constraining shapes . . . 80
F.3 Activation . . . 82

1. Linear Markovian dynamics, $\frac{d\mathbf{p}}{dt} = \mathbf{L}\mathbf{p}$, and cycles, are common

A. Mathematically, to first order, many dynamics are continuous time Markov chains. Also referred to as *kinetic schemes* (1) or viewed as representations of *chemical master equations* (2), continuous time Markov chains capture (approximately or exactly) how many systems change in time. When a single (possibly effective) typical timescale τ_{ij} (or rate $k_{ij} = 1/\tau_{ij}$) is used to describe a transition between every pair of states i and j, the description amounts to a continuous time Markov chain. Or, if the ith component $p_i(t)$ of the system's state probability evolves in time according to some function $f(\mathbf{p}(t))$ that depends on only the current state, we propose that a Taylor expansion to first order in $\mathbf{p}$ around a (hypothetical) empty system's state $\mathbf{0}$ also yields such a description,

$$\frac{dp_i}{dt} = f_i(\mathbf{p}(t)) \quad [1]$$

$$= \nabla \mathbf{f}_i^\top (\mathbf{p}(t) - \mathbf{0}) \;+\; (\mathbf{p}(t) - \mathbf{0})^\top \left(\frac{\partial^2 f_i}{\partial \mathbf{p}^2}\right)(\mathbf{p}(t) - \mathbf{0}) \;+\; \cdots \quad [2]$$

$$\approx \nabla \mathbf{f}_i^\top \mathbf{p}(t) = \sum_j \frac{\partial f_i}{\partial p_j} p_j(t) = \sum_j \frac{\partial \frac{dp_i}{dt}}{\partial p_j} p_j(t); \quad [3]$$

we can store these equations in a matrix form, defining $L_{ij} \equiv \frac{\partial \frac{dp_i}{dt}}{\partial p_j}$ to give

$$\frac{d\mathbf{p}}{dt} = \mathbf{L}\mathbf{p}. \quad [4]$$

Armed with the fact that total probability is conserved, $\sum_i p_i = 1$, one can further immediately conclude that

$$\frac{d}{dt}\left(\sum_i p_i\right) = \sum_i \frac{dp_i}{dt} = 0 \quad [5]$$

$$= \vec{1}^\top (\mathbf{L}\mathbf{p}) = 0, \quad [6]$$

and since this must hold for arbitrary $\mathbf{p}$, we see that $\vec{1}^\top \mathbf{L} = \vec{0}^\top$, namely the rows of $\mathbf{L}$ sum to zero.* So the diagonal entries of $\mathbf{L}$ can be expressed as $L_{ii} = -\sum_{j \neq i} L_{ji}$.

B. Closed steady-state systems are either equilibrium or cyclic. Why can we conclude that a graph without cycles cannot show nonequilibrium steady-states (and so must be in detailed balance at steady-state)? Since this question is about graph structures and generic steady-states, we turn to the Matrix Tree Theorem, discussed more fully in the main text and illustrated in this supplement's §D, which insights come from the nature of spanning trees. We recall that a spanning tree of a graph G is a tree (a connected and acyclic subgraph of G) that includes all the vertices of G. Furthermore, a spanning tree *rooted* in a state i contains no outgoing edges from state i (and exactly one outgoing edge for every other state $j \neq i$). We denote by $T_i^{(}m)$ the product of all the rates participating in one of the tree rooted in i; the subscript m, being a simple label for the tree. These notions are summarized in the example of Fig. 3A in the main text.

First, recall that detailed balance occurs when for any pair of states (i, j), the steady-state probabilities satisfy,

$$p_i k_{ij} = p_j k_{ji} \quad [7]$$

*In more (indicial) words, $\sum_i \frac{dp_i}{dt} = \sum_i \left(\sum_j L_{ji} p_j\right) = \sum_j p_j \left(\sum_i L_{ji}\right) = \sum_j p_j \left(L_{jj} + \sum_{i \neq j} L_{ji}\right)$. Since this must hold true for any value of p_j, we see that $L_{jj} + \sum_{i \neq j} L_{ji} = 0$ for all states j, confirming the form of the diagonal entries of the matrix.

Next, consider how spanning trees in a graph are structured and their algebraic consequences. For any steady state, whether in or out of equilibrium, the statistical weight of a state i is the sum of spanning trees rooted in i,

$$\rho_i = \sum_{\text{span. trees } m} \underbrace{\prod_{k_{rs} \in \text{ tree } m} k_{rs}}, \tag{8}$$

$$= \sum_{\text{span. trees } m} T_i^{(m)}, \tag{9}$$

where we have included the algebraic reminder that some mth spanning tree $T_i^{(m)}$ rooted in node i is a product of suitable rate constants k_{rs} such that every node is visited exactly once and there is no outgoing edge from the root i. Plus, we recall that the Matrix Tree Theorem applied to a graph G states that $p_i = \frac{\rho_i}{\rho_{tot}}$, for each vertex i in graph G, with $\rho_{tot} = \sum_{i \textit{ vertex in } G} \rho_i$.

How are the spanning trees rooted in a node i related to those rooted in a connected node j? By structural requirement these trees are quite similar. Indeed, *if* a tree T_i rooted in i contains the edge k_{ji}, then we can always convert it to a valid spanning tree rooted in j instead by "flipping" that edge to contain k_{ij} instead, building the newly rooted tree $T_j = \frac{k_{ij}}{k_{ji}} T_i$. (This re-rooting works because the rest of the edges in the original i-rooted tree T_i have not been altered so still have out-degree exactly one; all the nodes in the graph are still visited by the tree; and now j has out-degree zero, as required of a valid spanning tree rooted in j.) If all the spanning trees rooted in i contain the edge k_{ji}, then this re-rooting operation works to build all the trees rooted in j, giving

$$p_j = \frac{\rho_j}{\rho_{tot}} = \frac{1}{\rho_{tot}} \sum_m T_j^{(m)} = \frac{1}{\rho_{tot}} \sum_m \frac{k_{ij}}{k_{ji}} T_i^{(m)} \tag{10}$$

$$= \frac{1}{\rho_{tot}} \frac{k_{ij}}{k_{ji}} \sum_m T_i^{(m)} \tag{11}$$

$$= \frac{k_{ij}}{k_{ji}} p_i, \tag{12}$$

which is exactly the requirement of detailed balance between i and j.

However, while every spanning tree of an acyclic graph (where i and j are connected) *will* contain the edge k_{ij} or k_{ji} (since there is one path in the graph allowing them to be connected), this is no longer true for graphs containing a cycle: other paths can connect i and j that do not directly contain the (i, j) edges and thus build valid spanning trees. In that case, we cannot always write $T_j = \frac{k_{ij}}{k_{ji}} T_i$ and so cannot factor out $\frac{k_{ij}}{k_{ji}}$ from the weights ρ_i and ρ_j. This means that only such cyclic graphs can violate detailed balance at steady-state.

B.1. Example of an acyclic system: the simple repression motif. This connection between structure and the impossibility of violating detailed balance is illustrated in the simple repression motif. Here, repressors are assumed to sterically exclude the polymerase's binding (3, 4); this condition permits just three states in a linear graph that lacks a cycle. Specifically, call "S" the empty genome substrate state; R the repressor-bound genome state; and P the polymerase-bound genome state. These states form the linear graph,

$$R \underset{k_{SR}[R]}{\overset{k_{RS}}{\rightleftharpoons}} S \underset{k_{PS}}{\overset{k_{SP}[P]}{\rightleftharpoons}} P. \tag{13}$$

Since there is only one rooted spanning tree per root state, the Matrix Tree Theorem says that the steady-state statistical weights of the states are

$$\begin{pmatrix} \rho_R \\ \rho_S \\ \rho_P \end{pmatrix} = \begin{pmatrix} k_{SR}[R]k_{PS} \\ k_{RS}k_{PS} \\ k_{RS}k_{SP}[P] \end{pmatrix}. \tag{14}$$

Thus, the *ratios* between these statistical weights must be $\frac{\rho_R}{\rho_S} = \frac{k_{SR}[R]k_{PS}}{k_{RS}k_{PS}} = \frac{k_{SR}[R]}{k_{RS}}$, and $\frac{\rho_P}{\rho_S} = \frac{k_{RS}k_{SP}[P]}{k_{RS}k_{PS}} = \frac{k_{SP}[P]}{k_{PS}}$.

Now we explicitly verify that given this special case of an acyclic architecture, these statistical weights are unchanged by imposing the further requirement of detailed balance. The condition of detailed balance is equivalent to stating that the input and output fluxes between any pair of nodes must equal,

$$\begin{cases} \rho_S k_{SR}[R] & = \rho_R k_{RS} \\ \rho_S k_{SP}[P] & = \rho_P k_{PS}. \end{cases} \tag{15}$$

We see at once that indeed, this statement of detailed balance is fully equivalent to the relative statistical weights we found by the Matrix Tree Theorem. (We need only consider $N - 1 = 2$ ratios in this case, by the normalization of total probability.) So as expected, the stationary probabilities found by the Matrix Tree Theorem further satisfy detailed balance, for this linear (acyclic) simple repression motif.

C. The cycle condition relates a ratio of rate constants to (non)equilibrium. In a graph composed of a single cycle of states, the net drive maintaining a nonequilibrium steady-state is related to the ratio of products of rate constants taken in opposing directions around the cycle (5). Here we pedagogically discuss this connection by showing that when this ratio is one, and the system is at steady-state, then the system must be at detailed balance, and vice versa.

Consider such a cyclic weighted graph composed of N nodes and $2N$ edges (encoding the bidirectional transitions); enumerate the states from 1 to N, and the corresponding edge weights as the rates $k_{i,i+1}$ and $k_{i+1,i}$ between neighboring nodes $(i, i+1)$. (In what follows, given the cyclic structure of the graph, we adopt the notational convention that indices are to be taken modulo N.) For notational convenience, define the product of rate constants in the clockwise (+; increasing index i direction) as

$$\gamma_+ \equiv \prod_{i=1}^{N} k_{i,i+1},$$

and the analogous product in the counter-clockwise direction as

$$\gamma_- \equiv \prod_{i=1}^{N} k_{i+1,i}.$$

Our goal is to show that when their ratio γ is unity,

$$\gamma \equiv \frac{\gamma_+}{\gamma_-} = \frac{\prod_{i=1}^{N} k_{i,i+1}}{\prod_{i=1}^{N} k_{i+1,i}} = 1 \tag{16}$$

and the system is at steady-state—namely that the net influxes and outfluxes balance for each node in graph,

$$0 = J_{i,i+1} - J_{i+1,i} + J_{i-1,i} - J_{i,i-1}, \forall i \in [\![1\ ;\ N]\!], \tag{17}$$

detailed balance is automatically satisfied, and vice versa. The detailed balance condition is that

$$J_{i,i+1} = k_{i,i+1}\rho_i = k_{i+1,i}\rho_{i+1} = J_{i+1,i}, \forall i \in [\![1\ ;\ N]\!]. \tag{18}$$

First, we verify the logical direction Detailed Balance, Eq. [18] $\Rightarrow$ (Steady State, Eq. [17] AND $\gamma = 1$, Eq. [16]). Rewriting the detailed balance condition Eq. [18] readily confirms this desired logical direction; specifically, we see,

$$\gamma = \frac{\prod_{i=1}^{N} k_{i,i+1}}{\prod_{i=1}^{N} k_{i+1,i}} = \frac{\Pi_{i=1}^{N} J_{i,i+1}}{\Pi_{i=1}^{N} J_{i+1,i}} = 1.$$

Next we verify the opposite logical direction, that Eq. [18] $\Leftarrow$ (Steady State, Eq. [17] AND $\gamma = 1$, Eq. [16]). Starting from the cycle condition of $\gamma = 1$ allows us to rewrite the influx through a given node m as $J_{m+1,m} = \dfrac{\prod_{j=1}^{N} J_{j,j+1}}{\prod_{j=1, j\neq m}^{N} J_{j+1,j}}$. The outflux through a node p is analogously $J_{p,p+1} = \dfrac{\prod_{j=1, j\neq p}^{N} J_{j,j+1}}{\prod_{j=1}^{N} J_{j+1,j}}$. Using these expressions to replace each of the four flux terms that appear in the steady-state condition Eq. [17], for all nodes $m \in \{i, i-1\}$ and $p \in \{i, i-1\}$, gives

$$0 = \frac{J_{12} \ldots J_{i-1,i} J_{i+2,i+3} \ldots J_{N1}}{J_{21} \ldots J_{i,i-1} J_{i+1,i+2} \ldots J_{1N}} \left[\left(\frac{J_{i+1,i+2}}{J_{i+2,i+1}} - \frac{J_{i,i+1}}{J_{i,i+1}} \right) \frac{J_{i-1,i}}{J_{i,i-1}} - \frac{J_{i+1,i+2}}{J_{i+2,i+1}} \left(\frac{J_{i,i+1}}{J_{i+1,i}} - \frac{J_{i-1,i}}{J_{i,i-1}} \right) \right]. \tag{19}$$

This expression simplifies to imply that the ratio of influxes to outfluxes must be the same across all pairs of edges :

$$\frac{J_{i-1,i}}{J_{i,i-1}} = \frac{J_{i+1,i+2}}{J_{i+2,i+1}} = H, \forall i \in [\![2\ ,\ N]\!], \tag{20}$$

with H being a constant defined by Eq. [20]. Last, substituting the condition Eq. [16] implies that $H = 1$, and therefore implies Eq. [18], completing the desired correspondence.

D. Discussion of various ways of quantifying dissipation. The field of nonequilibrium thermodynamics quantifies nonequilibrium using different mathematical quantities. The nonequilibrium driving force, also referred to as the net (chemical) drive, is one key quantity. For a single cycle, the net drive $\Delta\mu$ is the net difference in chemical potential, namely free energy, imposed by one progression around the cycle along the nonequilibrium steady-state flux (5), (6, Ch. 13). For a single cycle, this net drive is related to the cycle parameter γ we have just discussed in the previous subsection via

$$\Delta\mu = k_B T \ln \gamma. \qquad [21]$$

The units of this nonequilibrium driving force are energy ($k_B T$); in view of its centrality in describing nonequilibrium steady-states, this net drive is the quantity we use to analyze nonequilibrium in this paper.

Another related, central quantity that governs nonequilibrium behavior is the dissipation rate, or entropy production rate, which for a single cycle (at steady-state) is

$$\dot{W} \equiv \frac{1}{T}\Delta J \Delta\mu = \left(J_{i,i+1} - J_{i+1,i}\right)\, k_B \;\; \ln\gamma, \qquad [22]$$

where $\Delta J = J_{i,i+1} - J_{i+1,i}$ is the nonequilibrium steady-state's net flux difference along any of the cycle graph's edges.

Interestingly, note that Eq. [22] makes clear that even if a cycle requires a finite net drive $\Delta\mu \neq 0$ to maintain a nonequilibrium probability distribution over states, if the system is made to operate slowly enough—by reducing the magnitudes of all rates (hence fluxes J) simultaneously (while retaining their relative imbalances, e.g. in the same γ and hence the same $\Delta\mu$)—the entropy production rate can be made arbitrarily small, $\dot{W} \to 0$. (Since our chief focus is on the statically controlled, steady-state behavior of regulatory systems, we do not analyze the entropy production rate in this paper, in favor of the net drive $\Delta\mu$.)

E. Other destinies of biochemical dissipation in cells beyond gene expression. In this work, we confine our study to focus on the impact of dissipation on quantitative behaviors most relevant to gene regulation. However, naturally, other crucial biological behaviors are also modified when energy is spent. While beyond the scope of this paper, a wide, fertile, and creative literature has dissected the quantitative consequences of nonequilibrium in contexts besides transcription. For the further interest of the reader, we give a (necessarily incomplete) list of some references explicating some of these separate behaviors below.

- **Kinetic proofreading.** Energy investment can improve the ability of enzymes, polymerization systems, or assembly systems to incorporate "correct"/cognate substrates in favor of spurious substrates, over the discrimination offered by equilibrium energy differences alone; see classic references by Hopfield (7) and Ninio (8), along with modern progress in reports like those by Murugan and coworkers (9) and beyond.

- **Adaptation and receptor response.** Cells executing sensing can use energy to tune the environmental level of a stimulus at which they are most sensitive, for example to continue following a chemotactic gradient along a changing baseline. See work reported in references such as Lan *et al.* (10), Barato *et al.* (11), and Hartrich *et al.* (12).

- **Ultrasensitivity.** The maximal slope of an input-output curve can be made larger under energy expenditure; this manifests in the famous classic Goldbeter-Koshland (13) scheme, in addition to recent works such as de Los Reyes *et al.* (14) and Qian (15).

- **Noise reduction.** The impact of noise in biochemical circuits can be tuned and countered by dissipation; see work such as those by Govern and coworkers (16, 17).

- **Dynamic behavior such as oscillation.** Coherent oscillations in biochemical state spaces can be improved with dissipation; see discussions described by references such as Barato *et al.* (18).

- **Directional transport by molecular motors.** Molecular motors accomplish hugely important transport of cargo in quasiballistic trajectories powered by free energy transduction; see syntheses such as Howard (19).

- **Accurate, precise self-assembly.** The accuracy, speed, and sophistication of molecular self-assembly can be tuned by dissipation; see creative recent work such as References (20–23).

We thank a considerate Reviewer for recommending inclusion of such references. In addition, we refer the reader to texts such as *The Restless Cell* (6), Terrell Hill's classic text *Free Energy Transduction and Biochemical Cycle Kinetics* (24), and beyond for broader context.

2. Insights into the square graph

A. The simple four-state cycle motif pervades prokaryotic and eukaryotic gene regulation. First, we review the state of knowledge about regulation in *E. coli*, and *D. melanogaster*, by extracting fresh up-to-date empirical histograms of regulatory architectures and their incidence. This analysis results in the histograms reported in Figure 2 of the main text. The data of interest are how many distinct transcription factors (whether distinct in biological identity or not) can simultaneously occupy each promoter or gene; this gives a putative upper bound on the dimensionality of the state space of the underlying graph.

A.1. Up-to-date census of known regulatory interactions in E. coli. To understand what state space topologies are most common in *E. coli*, we reanalyzed the bipartite[†] network of all known regulatory interactions between transcription factors and promoters, as reported in the most recent release 11.2 of the canonical database *RegulonDB* (25). These data report 216 unique transcription factors and 1152 unique regulated promoters. During data processing, we parsed all interactions (retaining all interactions, reported as strong or weak evidence; this choice does not significantly the incidences observed). See https://www.its.caltech.edu/~gsalmon/gs069_Ecoli_biadjacency_RegulonDB11pt2.html for an interactive version of a heatmap where individual interactions can be gleaned by hovering with a mouse.

We then counted each distinct binding site according to whether the transcription factor is reported to be repressing, activating, or more rarely both at each distinctly-positioned binding site on the genome. Fig. S1 schematizes this count; the indicated layers stratify both the type of interactions (indicated by layer color) and whether the interactions are annotated with the required granularity of the number of binding sites between the interacting transcription factor and promoter (indicated by dotted or solid layer outline).

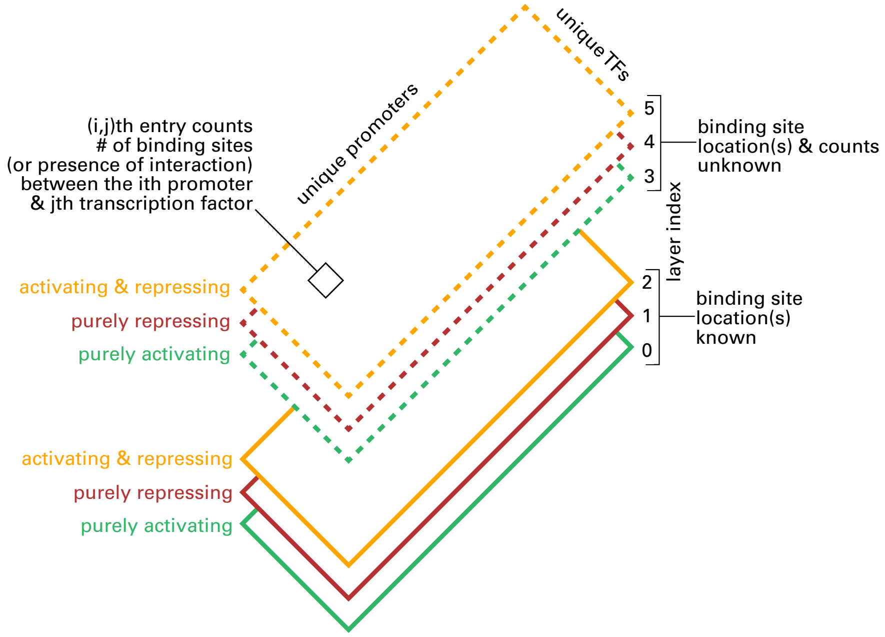

Fig. S1. Categorical classification of counts of binding sites between promoters and transcription factors, separately acknowledging regulatory interactions where the locations or counts of binding site are not known/reported (dashed outlined layers). Since interactions without any localized binding sites cannot report how many individual transcription factors plausibly reside on the given promoter, the entries without specific binding site locations reported (layers 3-5) were omitted from future counts.

Next, we chose and followed a convention for counting the regulatory architectures achievable by the rare but present cases of binding sites identified as both repressing and activating.

[†] A network is called *bipartite* if its nodes can be divided into two classes, and all edges in the network connect nodes of one class to the other class. This is naturally the case for how promoters and transcription factors interact.

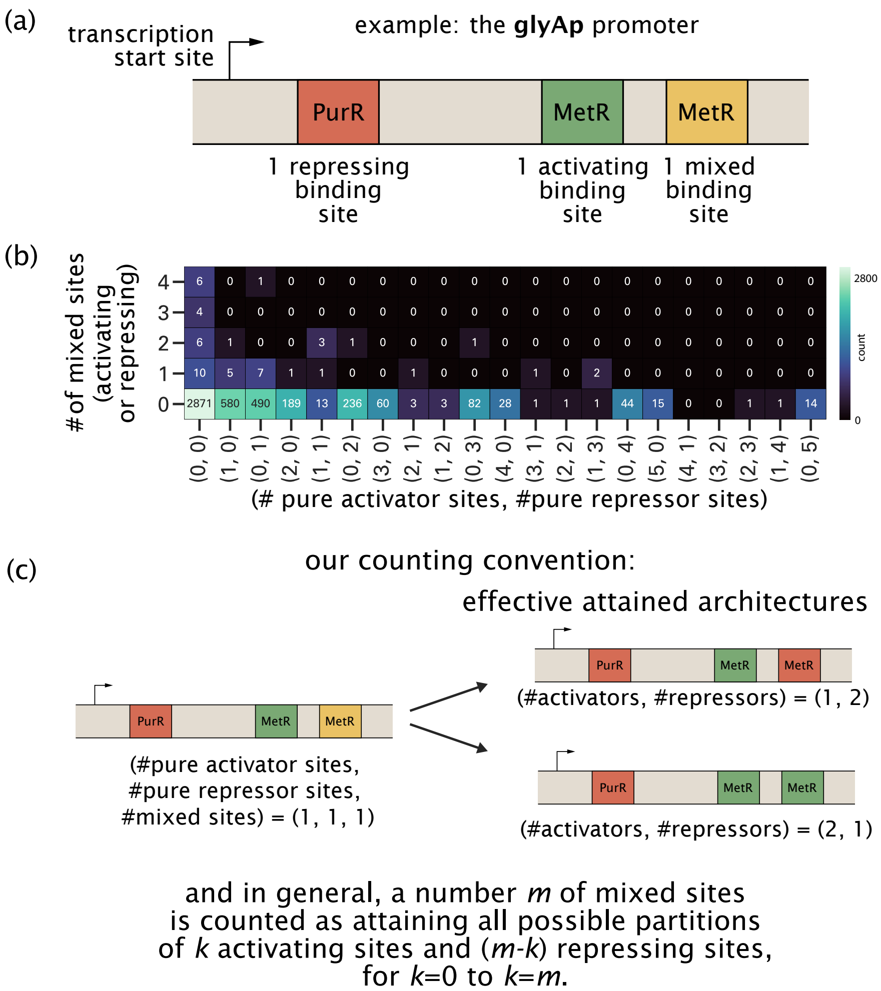

Fig. S2. Accommodating cases in the regulatory network where individual binding sites (on a promoter for a particular transcription factor) are reported to be both activating and repressing. (A) One example of this type of mixed binding site is found in the glyAp promoter, where repressing, activating and mixed binding sites are respectively represented in red, green and yellow. (B) Heatmap showing counts of these mixed binding sites as rows, against columns depicting different counts of binding sites annotated as pure activating or repressing. Mixed sites are uncommon, but present. (C) Since such mixed sites can act as either repressors or activators, we adopt the convention of counting all possible effective architectures where the mixed binding sites assume either repressing roles (e.g. top) and/or activating role (e.g. bottom)s. Mathematically, this means that when there are m mixed binding sites, k sites can act as extra activating sites and $(m-k)$ can act as extra repressing sites, for $0 \leq k \leq m$. This convention allows us to collapse the architectures to represent examples of some number of activators and some number of repressors on the promoter.

As illustrated in Fig. S2, when we see a promoter with r purely repressive binding sites; a purely activating binding sites; and m sites that can be either repressing or activating, we count this scenario as attaining multiple architectures, namely the set $\{(a+k, r+(m-k))\}_{0\leq k\leq m}$. Note that this convention only (very mildly) shapes the observed empirical distributions of architectures, since appreciably less than a percent of all binding sites are annotated as both repressing and activating. Further, by construction, this convention does not affect the total copy number of transcription factors that are counted per promoter, the number of interest in defining what state space topologies promoters have.

The resulting raw counts of regulatory architectures are summarized in S3.

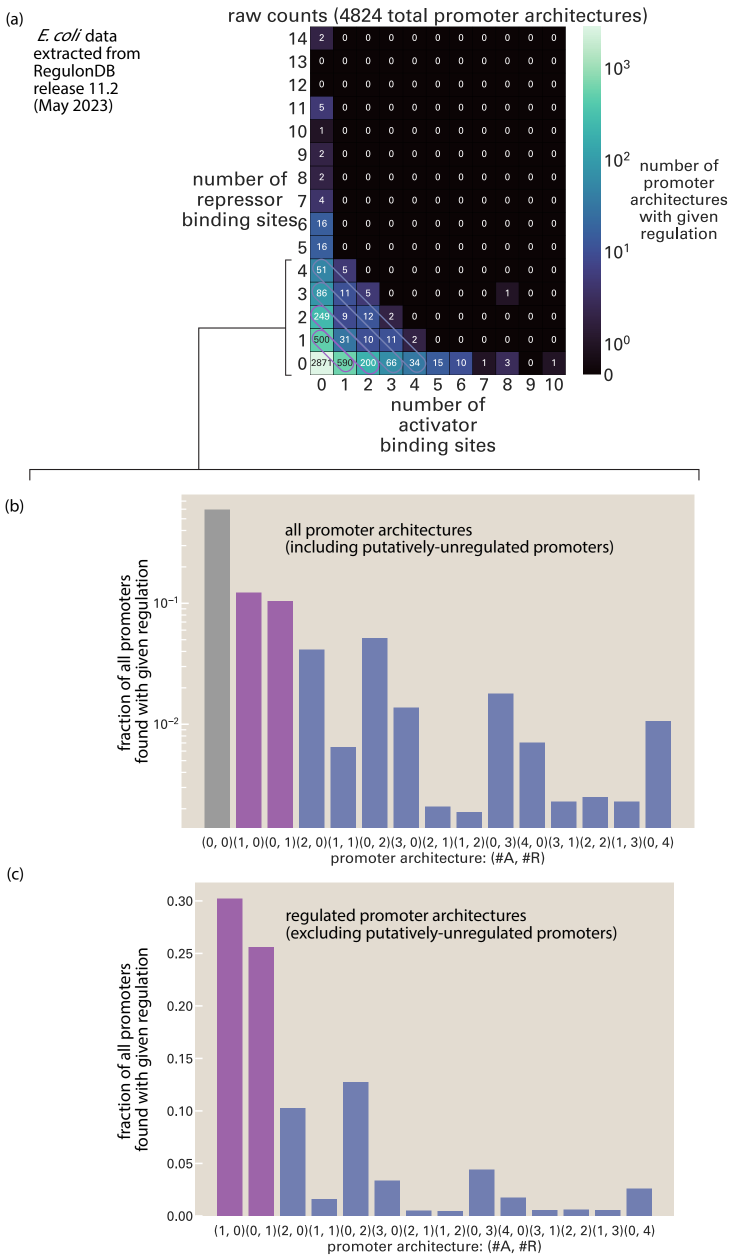

Fig. S3. Incidence of regulatory architectures reported in *E. coli*, according to data in the RegulonDB database (25) release 11.2 (May 2023), organized by the joint numbers of activating and repressing transcription factors associated with each promoter. Architectures are regarded as the copy number of possibly-non-distinct proteins that can be bound to sites on each promoter. (A) The joint empirical distribution of the number of repressors and activators is largely symmetric (that is, $p(\#A, \#R) \approx p(\#R, \#A)$). (B) The joint distribution projected on a one-dimensional histogram on a logarithmic y-scale, including putatively-unregulated promoters with no known regulatory interactions (in grey) that comprise the majority ($\sim 71\%$) of promoters. Purple bars highlight promoters reported to have one associated transcription factor. (B) The incidence of regulatory architectures among those promoters with at least one known regulatory interaction. Over half of these regulated promoters are reported to be be regulated by the most common architecture of a single transcription factor (purple bars), and thus are plausibly describable by the square graph of states analyzed most thoroughly in the main text. (This figure forms the basis of Fig. 2A in the main text.)

We also note that this observed regulatory architectures from an aggregating database is also highly compatible with the

results of individual concerted experimental surveys.

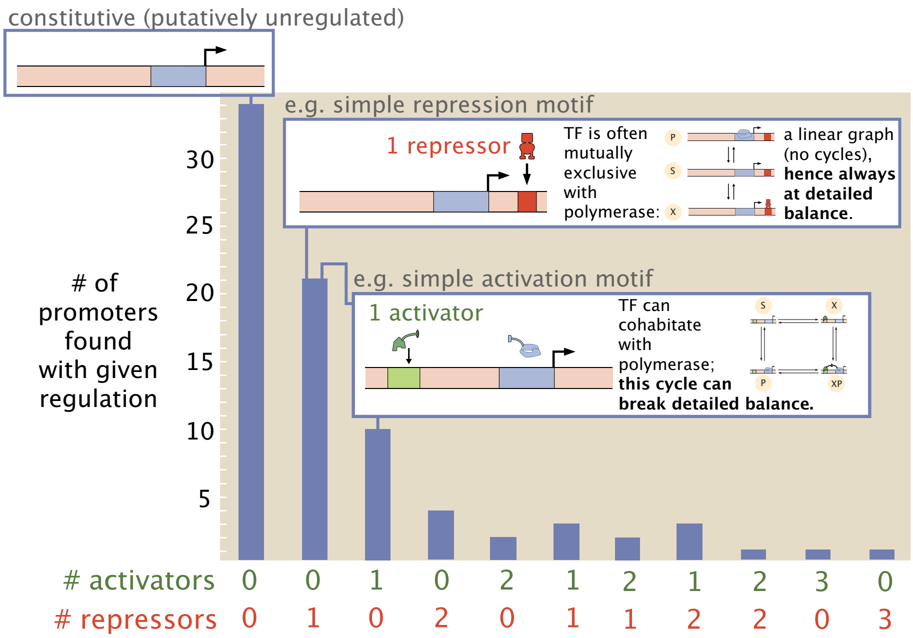

Fig. S4. An experimental histogram of empirically-observed gene regulatory motifs in *E. coli* (26) reveals that many promoter sites are regulated by a single repressor or activator. A single repressor can often implement the simple repression motif, where the repressor excludes the polymerase from binding, allowing just three states in a linear graph. (Raw histogram data are courtesy of Reference (26).) The reason that the "simple activation motif" is schematized as linked to both the (0 activator, 1 repressor) and (1 repressor, 0 activator) histogram bar is that while steric exclusion commonly occurs for repressors, often making single repressors well described by a linear graph of three states, some repressors do not completely exclude the polymerase, permitting a cycle motif too.

Reference (26) is among the widest experimental censuses discovering regulatory interactions in *E. coli* in the recent literature. This study also found that transcriptional architectures with one activator or repressor are the most commonly observed regulated transcriptional architectures.

Thanks to common steric overlaps between the repressor binding site and polymerase binding site (27), a repressor is often—though not necessarily—mutually exclusive with the polymerase (see Fig. S4). In this case, the repressor implements a simple repression motif, a graph which lacks a cycle (3). However, when the repressor does not sterically exclude the polymerase, a cycle of four states emerges. The same cycle of four states emerges with activators, whose binding sites rarely directly overlap with the polymerase binding site (27); this produces a "simple activation motif." These observations affirm that a single cycle of four states is a common motif in prokaryotic gene regulation.

A.2. Up-to-date census of known regulatory interactions in D. melanogaster. Equivalents of such a four-state cycle also occur regularly in models of eukaryotic gene regulation (28–31). To more quantitatively assess the putative commonality of regulatory architectures up to the literature's state of regulatory knowledge, we analyzed transcription factor-gene interactions reported by the most recent release v9.6.2 of the *Redfly* database (32). These data report only 318 reported unique regulated genes, governed by only 213 unique transcription factors. See https://www.its.caltech.edu/~gsalmon/gs069_Dmelanogaster_biadjacency_Redfly202310.html for an interactive version of a heatmap where individual interactions can be gleaned by hovering with a mouse.

We counted the total maximum putative copy number of transcription factors (total number of distinct binding sites) associated with each gene. While more diverse regulation appears more common in *Drosophila*, the results, summarized in Figure S5, appear to emphasize the pervasiveness of a putatively-four-state regulatory architecture, given by a single transcription factor binding site annotated per gene.

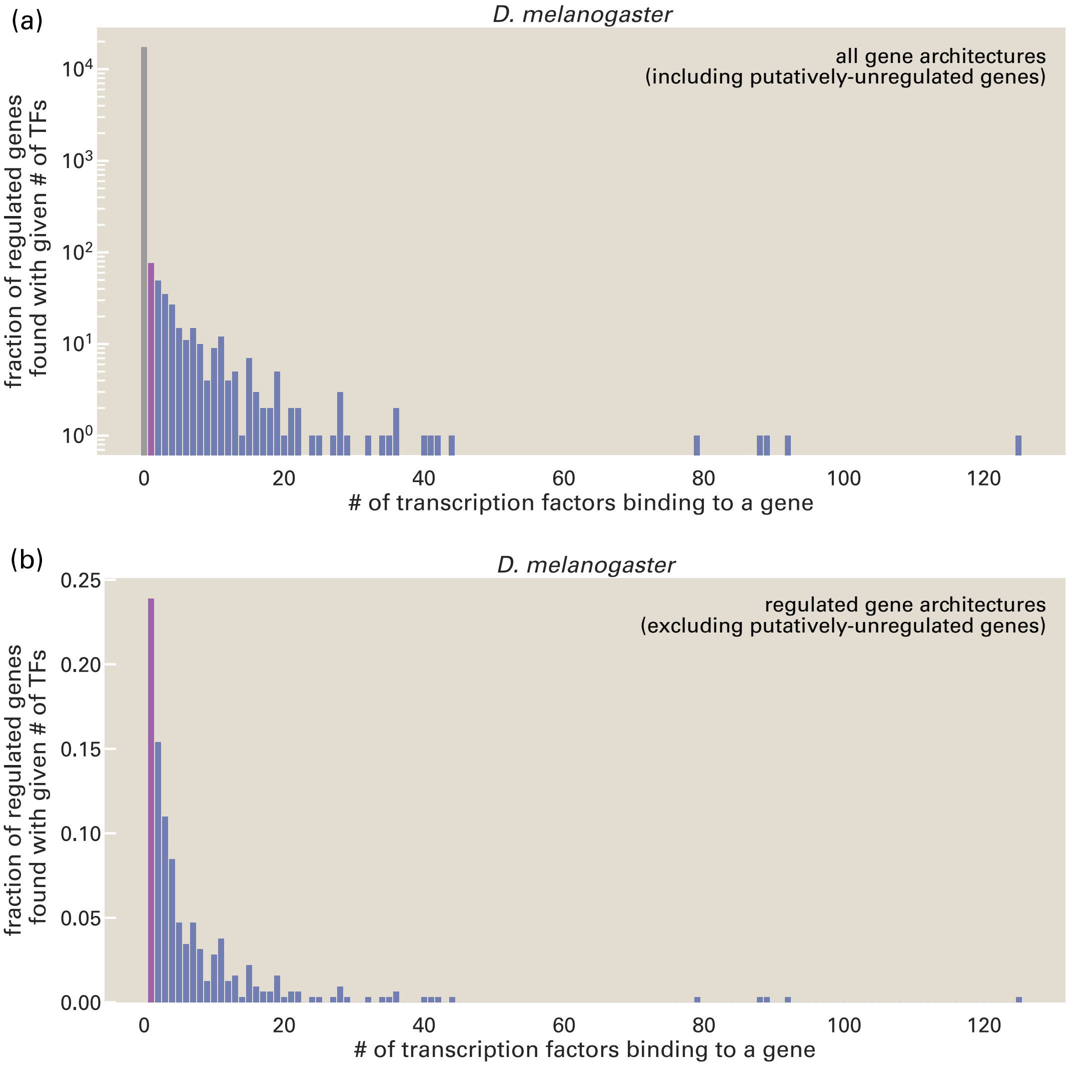

Fig. S5. Histograms of the copy numbers of transcription factors capable of interacting with each gene in *Drosophila*, equivalent to the numbers of transcription factor reported binding sites per gene, according to data reported the Redfly database (32) v9.6.2 as of October 2023. (A) Histogram of the number of distinct transcription factors reported to bind a gene, including the putatively-unregulated genes in Drosophila (gray bar). (B) Histogram of the number of transcription factors associated with genes with at least one reported regulatory transcription factor; the purple bar represents the large proportion (about half) of regulated genes reported to be regulated by a single transcription factor. These data (up to five transcription factors) form the inset of Fig. 2A in the main text.

However, we stress that this survey reported in Fig. S5 should be regarded as highly provisional, betraying a poverty of consolidated regulatory information in eukaryotes. *D. melanogaster* has a total of total of $\sim 17,728$ genes (33); since Redfly reports only 318 regulated genes, this highlights that only $\approx \frac{318}{17728} \sim 1.8\%$ of genes have any regulatory interactions reported in canonical databases. Given this state of substantial regulatory ignorance—which itself argues for wider quantitative interrogation and centralized reporting of genetic regulation in eukaryotes—it is prudent to regard the apparent commonality of the square graph in *Drosophila* as a potential artifact of incomplete knowledge in the field, rather than a finding robust across eukaryotes were they to be rigorously characterized.

Indeed, consistent with their distinct architectural constraints, eukaryotes often have a greater number of binding sites associated with each gene than in prokaryotes (34, 35). These features potentially give eukaryotes a greater capacity for more combinatorial regulation by transcription factors than prokaryotes.

B. Order of magnitude estimated rate constants for prokaryotic transcription. Here, to allow us to assess how accessible interesting regulatory shape phenotypes are in the vicinity of biological rate parameters, we estimate default equilibrium rates typical of transcriptional systems in prokaryotes.

First, we remark that the correspondences between thermodynamic and kinetic viewpoints discussed in §G.3—specifically, Eq. [62]—provides the following parameter correspondences useful for our estimates:

$$\begin{cases} K_1 = \frac{k_{PS}}{k_{SP}} = C_N e^{\beta \Delta \epsilon_{pd}} \\ K_2 = \frac{k_{XP,P}}{k_{P,XP}} = C_N e^{\beta(\Delta \epsilon_{xd} + \epsilon_{xp})} \\ K_3 = \frac{k_{XP,X}}{k_{X,XP}} = C_N e^{\beta(\Delta \epsilon_{pd} + \epsilon_{xp})} \\ K_4 = \frac{k_{XS}}{k_{SX}} = C_N e^{\beta \Delta \epsilon_{xd}} \end{cases}, \quad [23]$$

where we have defined four equilibrium constants $K_1, \ldots, K_4$ (with units of concentration); C_N is the molar concentration of non specific sites in the cell, $\Delta\epsilon_{yd}$ is the energy difference between a state where molecule Y is bound specifically to the genome versus nonspecifically; and ϵ_{xp} is the interaction energy between the transcription factor X and the polymerase P. The concentration C_N can be expressed by the following formula :

$$C_N = \frac{N}{N_A V_{cell}} \quad [24]$$

where N_A is Avogadro's number; V_{cell} is the volume of the cell, usually taken here to be that characteristic of *E. coli*, $V_{cell} \approx 1\ \mu m^3$; N is the number of non specific binding sites. We will assume that the number of non specific sites is $N = 5 \times 10^6$, like in reference (36), which gives us $C_N \approx 10^{-2}\ M$.

Furthermore, the concentration $[P]$ and $[X]$, can be written in terms of their respective absolute copy number P and X :

$$\begin{cases} [X] & = \frac{X}{N_A V_{cell}} \\ [P] & = \frac{P}{N_A V_{cell}} \end{cases}. \quad [25]$$

Conveniently, $1\ nM \approx \frac{1}{N_A V_{cell}}$: a natural unit for the rate constants depending on the concentration of transcription factor or polymerase is $s^{-1} nM^{-1}$.

Armed with these conventions, we now estimate the order of magnitude of governing rate constants from available measurements and empirical data.

- First, we consider plausible binding e.g. **on-rates** of polymerase or transcription factors to the genome.
  - Taking the Lac repressor as evocative of transcription factors, three empirical measurements give plausible on-rate values, and illustrate some empirical variation:
    - Ref. (37) (BNID 106392; (38)) reports a $k_{on} \approx 2.8 \times 10^7\ s^{-1}\ M^{-1} = \boxed{2.8 \times 10^{-2}\ s^{-1}\ nM^{-1}}$ for the Lac repressor.
    - Ref. (39) (BNID 104607; (38)) reports an appreciably larger association rate of $k_{on} \approx 7 \times 10^9\ s^{-1}\ M^{-1} = \boxed{7\ s^{-1}\ nM^{-1}}$.
    - In their SI, reference (40) report that they took measurements from a paper by Hammar *et al.* (41), who in their Fig. 2 report (from single molecule, *in vivo* measurements) that in *E. coli*, it takes the Lac repressor an average time of about $\tau_{on} \approx 30\ s$ to bind to O1 or Osym operator sites. The later reference (40) report without citation that the copy number of Lac repressors in this older paper's setting was in fact about 4 copies per cell ($\approx$ 4 nM). This implies an association rate of about $k_{on} \approx \frac{1}{\tau_{on}\ c} \approx \frac{1}{30\ s\ \times 4\ nM} \sim \boxed{10^{-2}\ s^{-1}\ nM^{-1}}$.

    An intermediate average of these various empirical data suggest a few tenths of a nanomolar per second is a reasonable scale for the basal association rate.
  - We compare the empirical measurements above with an order-of-magnitude theoretical estimate presuming diffusion-limited binding. RNAP's binding site is approximately $20 - 34$ bp long; each base-pair is separated by 3.4Å (42); so the characteristic scale a we could expect of this binding site is about $a \approx 9$ nm. The diffusion coefficient of polymerase is $D_{poly} \approx 0.4 \mu m^2/s$ (43), while the (effective, *in vivo*) diffusion coefficient for LacI is $D_{LacI} \sim 0.4 \mu m^2/s$ (BNID 102038; (38); this effective diffusion constant for LacI plausibly reflects both 3D diffusion between nonspecific binding events and 1D genome-associated diffusion (43)). Reference (44) reports that the apparent (3D) diffusion coefficient of RNA polymerase II in the nucleus is (1-5) $\mu m^2/s$, similar to other transcription factors. (Altogether, these values indicate taking a diffusion constant of about $D \sim 1\ \mu m^2/s$ is reasonable.) A diffusion limited on-rate calculation then predicts that

$$k_{on} = 4\pi D a \sim 12(1 \mu m^2/s)(9 \times 10^{-3}\ \mu m) \sim 0.11\ /s \underbrace{\mu m^3}_{(1/0.602) nM^{-1}} = 0.17/s/nM \sim \boxed{10^{-1}/s/nM}. \quad [26]$$

    Compared to the orders of magnitude of this on rate found in the literature (varying between $10^{-2}/s/nM$ (37, 40) and $10^1/s/nM$ (39)), this approximated rate by a diffusion limited process is a good middle ground.
- Next we appraise characteristic energy scales among transcription factors, polymerase, and the specific sites on the genome:

- According to Ref. (36) (BNID 103594; (38)), the polymerase binds more favorably to the Lac specific binding site than nonspecific sites on the genome by an energy difference of about $\Delta\epsilon_{pd} \approx -2.9\ k_B T$ so $\boxed{\beta\Delta\epsilon_{pd} \sim -3}$.
- Ref. (45) reports that the Lac repressor preferentially binds to the specific operator binding sites with energies ranging from $\Delta\epsilon_{xd} \approx -15.3\ k_B T$ (for the O1 site) to $\Delta\epsilon_{xd} \approx -9.7\ k_B T$ (for the O3 site). So we take as representative $\boxed{\beta\Delta\epsilon_{xd} \sim -13}$.
- Ref. (36, Fig. 2) (BNID 103591; (38)) reports that the CRP activator interacts with RNAP with an interaction energy of approximately $\boxed{\beta\epsilon_{xp} \sim -4}$.

By using known binding energy values, the dissociation equilibrium constants depicted in Eq. [23] can be computed. By accounting for the diffusion-limited value of k_{on} as stipulated in Eq. [26], the respective off rates can be determined as follows: $k_{off} = K_D k_{on}$. Since the transcription factors plausibly stick to the genome by a factor $K_1/K_4 \approx \exp(-\beta\Delta\epsilon_{xd} + \beta\Delta\epsilon_{pd}) \sim \exp(13-3) = \exp(10) \sim 2 * 10^4$ stronger compared to the polymerase's interaction with the genome (36), it remains apparent that their equilibrium dissociation constants exhibit significant disparity. Therefore, assuming uniformity in the on rates of polymerase and transcription factor, the magnitudes of their off rates still differ by several orders of magnitude. So, any few-fold difference in the on-rate of polymerase to the genome (compared to the on-rate of the transcription factor to the genome) is not likely to be hugely significant in estimating $k_{off} = K_D k_{on}$. Therefore we make the reasonable assumption of taking the on-rates of polymerase and transcription factor to be essentially the same (diffusion-limited) value:

$$\boxed{k_{on} \sim 0.1/s/nM}.$$

- We recall that $1\ nM \approx \frac{1}{N_A V_{cell}}$ and we consider that the polymerase copy number is about $P \approx 10^3$ copies per cell (45). This suggests $[P] = \frac{P}{N_A V_{cell}} \approx 10^3\ nM$ and we estimate $k_{SP}[P] \equiv k_{X,XP}[P] \approx \left(0.1\ s^{-1}\ nM^{-1}\right)(10^3\ nM) \approx \boxed{10^2\ s^{-1}}$.
- While it is precisely how variation in the concentration $[X]$ tunes transcription that we are interested in, it is still instructive to report typical ranges for these transcription factor concentrations. As summarized in (42) (namely http://book.bionumbers.org/what-are-the-copy-numbers-of-transcription-factors/), cellular censuses show that repressing transcription factors typically have between $10 - 10^3$ copies per cell and activating transcription factors typically have between $1 - 10^2$ copies. This implies $[X] \sim$ few $\times\ 10^2$ nM. So ignoring the very variation in $[X]$ we're interested in, point estimates for $k_{SX}[X] \equiv k_{P,XP}[X]$ are $\approx (0.1\ s^{-1}\ nM^{-1})(\text{few} \times 10^2\ nM) \approx \boxed{\text{few} \times 10\ s^{-1}}$.

Altogether, these estimates can enter Eq. 23 and imply an approximate, default set of all rates. We summarize these order of magnitude values in Figure 1A of the main text and the table S1 below. (In the later analyses examining the consequences of drive along individual edges or pairs of edges, we choose and analyze more precise sets of default rate values consistent with these orders of magnitude; see Figures S15 and S16.)

rate	meaning	calculation	order of magnitude estimate
k_{XS}	unbinding of TF from empty genome	$k_{SX} C_N e^{\beta\Delta\epsilon_{xd}}$	$0.8\ s^{-1}$
k_{XPP}	unbinding of TF from RNAP-bound genome	$k_{PXP} C_N e^{\beta(\Delta\epsilon_{xd}+\epsilon_{xp})}$	$2 \times 10^{-2}\ s^{-1}$
k_{SX}	binding of TF to empty genome	$:= k_{on}$	$0.1\ s^{-1}\ nM^{-1}$
k_{PXP}	binding of TF to RNAP-bound genome	$:= k_{on}$	$0.1\ s^{-1}\ nM^{-1}$
k_{PS}	unbinding of RNAP from empty genome	$k_{SP} C_N e^{\beta\Delta\epsilon_{pd}}$	$2 \times 10^4\ s^{-1}$
k_{XPX}	unbinding of RNAP from TF-bound genome	$k_{XXP} C_N e^{\beta(\Delta\epsilon_{pd}+\epsilon_{xp})}$	$5 \times 10^2\ s^{-1}$
k_{SP}	binding of RNAP to empty genome	$:= k_{on}$	$0.1\ s^{-1}\ nM^{-1}$
k_{XXP}	binding of RNAP to TF-bound genome	$:= k_{on}$	$0.1\ s^{-1}\ nM^{-1}$

Table S1. Summary of orders-of-magnitude estimates of rates at equilibrium that govern transcription. Here, C_N is the volumetric concentration of N nonspecific sites on the genome; we note that $C_N = \frac{N}{N_A V_{\text{cell}}} = N(1\ nM)$. In the column *calculation*, we write down some expressions for the rate constants, from first principles that we evaluate at some values based on measurements reported in the literature. We took $\beta\Delta\epsilon_{xd} = -13$ (source: (45)), $\beta\epsilon_{xp} = -4$ (36, 43), $\beta\Delta\epsilon_{pd} = -3$ (36, 43), $N = 5 \times 10^6$ (43), and $k_{on} = 4\pi Da$, with $D = 1\mu m^2/s$ (38, 43, 44) and $a = 9nm$ (42).

C. Biologically, timescales are plausibly separated enough that transcription is well represented by small Markov chains.

(A)

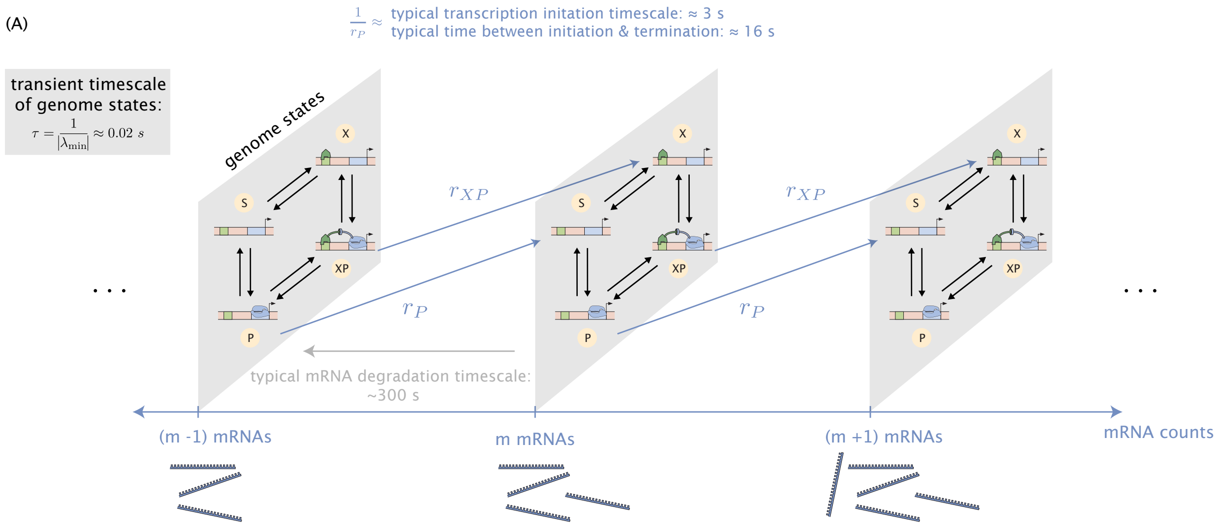

(B) The magnitudes of eigenvalues λ<0 of the Laplacian L are decay rates that set how slowly **p**(t) transiently approaches steady state; this decay is dominated by the slowest rate $\lambda_{\min}$.

Assuming that the abundance of the transcription factor is about [X] ~ 200 copies/cell = 200 nM; and the polymerase concentration is [P] ~ 10^3 copies/cell = 10^3 nM, the Laplacian is

$$\rightarrow \mathbf{L}_{\text{genome}} = \begin{array}{c|cccc} & \multicolumn{4}{c}{\text{source state}} \\ & S & X & XP & P \\ \hline S & -120 & 0.8 & 0 & 20000 \\ X & 20 & -100.8 & 500 & 0 \\ XP & 0 & 100 & -500.02 & 20 \\ P & 100 & 0 & 0.02 & -20020 \end{array} \; s^{-1}$$

(rows: destination state)

and computing the eigenvalues, we find that the smallest decay rate $\lambda_{\min} \approx -20\ s^{-1}$ is fast relative to transcription or degradation.

Fig. S6. A separation of timescales between transcription and binding (or unbinding) is well justified, for the order-of-magnitude rate constant estimates we adopt to model transcription. Panel (A) depicts a fuller state-space graph of transcription, accounting for not just states of the genome (bound or unbound by transcription factors and polymerase), but also the current count of mRNA transcripts. This graph is composed of many copies of the four-state graph, each indexed by the current mRNA count; transitions among these layers is accomplished by successful transcription by polymerase or by mRNA degradation. Transitions from layers at high mRNA count to lower mRNA count (corresponding to degradation by mRNA, denoted by a light gray arrow) can be neglected in this picture, since mRNA degradation is much slower than the other events of interest. Panel (B) shows the Laplacian matrix $\mathbf{L}_{\text{genome}}$ of each four-state graph layer describing transitions among the genome states. This matrix gives a governing second-largest-eigenvalue of $\lambda_{\min} \approx -20\ s^{-1}$, which sets the timescale for the Markov chain to equilibrate over genome states conditioned on a specific mRNA count. The fact that this eigenvalue is fast relative to the transitions between layers allows us to collapse this multi-layer graph into study of just a four-state network inside each layer.

Technically, gene expression is governed by a fuller chemical master equation than that defined by merely the states of the genome. In principle, the current number of mRNA transcripts could affect the allowed transitions, and *a priori* one might worry that an additional mechanism to transition from a state where the polymerase is bound to the genome (P or XP) to a state where it is unbound (S or X) is when the polymerase has transcribed a transcript successfully enough to vacate the polymerase binding site. These technicalities would in fact imply a larger, fuller ladder of states that define the discrete state Markov chain, as visualized in Figure S6. However, here we argue that both the time between initiation events and the time taken by the polymerase to actually transcribe along the genome are typically much longer than the equilibration timescale of the four states of the genome alone. This separation of timescales formally justifies the assumption that the net accumulation of mRNA transcripts is proportional to the probability of being in the polymerase-bound states.

First, we estimate the rate at which the count of mRNA transcripts accumulate once the polymerase is bound. RNAP elongates nascent transcripts at a rate of about 3.72 kb/min in *E. coli* (BNID 103021; (38)); this is $v = 62$ nucleotides/second. The average protein is $L_p \approx 340$ peptides long (BNID 10895; (38)), implying that protein-coding mRNAs are about $3L_P \approx 10^3$ nucleotides long, consistent with reports elsewhere of mean mRNA lengths of 924 nt across prokaryoates (46). Hence, once transcribing, it takes approximately $\tau_{\text{transcribe}} \approx \frac{3L_P}{v} \approx 1000/62 \approx 16$ seconds to serially transcribe a typical gene. This is a lower bound on the accumulation rate, however, since the RNAP can leave the promoter faster than a transcript is complete, permitting a larger transcription initiation rate. In *E. coli*, transcription initiation has been reported to occur at a typical rate of 20 initiations/min/gene, or at a rate of ~ 0.3 initiations per second (BNID 111997; (38)). Therefore, in the fuller lattice of states of a Markov chain explicitly tracking mRNA counts (Fig. S6), the rates of transitions from states with count m to states with count $m+1$ are plausibly between the lower bound of $r_{\text{transcribe}} = 1/\tau_{\text{transcribe}} \approx 1/16s \approx 0.06\ s^{-1}$ and an upper bound of $r_{\text{initiate}} \approx 0.3\ s^{-1}$, or in summary, we take $r \sim \boxed{\text{few} \times 10^{-1}\ s^{-1}}$. In addition, degradation is even slower: the typical half-life of an mRNA in *E. coli* is reported to be on the order of a few minutes (BNID 108598; (38)), implying the degradation

rate (governing how quickly m mRNAs could decrement to $m-1$ mRNAs) is on the order of $\gamma_d \sim$ few $\times\, 10^{-3}\ s^{-1}$.

In contrast, the slowest timescale within which the four genome states converge towards their steady-state distribution—set by the smallest magnitude eigenvalue of the four state Laplacian matrix of transition rates for the genome—is approximately $1/20 \approx 0.05$ seconds (see Fig. S6 for the calculation). This is much faster than the transcriptional transition timescales. Therefore, the condensation of the larger ladder graph into the smaller graph of just four binding and unbinding reactions on the genome is justified, for this particular set of plausible rate constants.

D. Deriving the universal form: The Matrix Tree Theorem on the square graph yields a ratio of quadratic polynomials. Applying the Matrix Tree Theorem to derive steady-state probabilities p_i of each state i, and hence any response observable $\langle r \rangle \equiv \sum_{\text{states } i} r_i p_i$, reveals that these responses follow the following universal form,

$$\boxed{\langle r \rangle = \frac{A + B[X] + C[X]^2}{D + E[X] + F[X]^2}}, \qquad [27]$$

where the coefficients are given by weighted sums of spanning trees with different possible $[X]$-dependencies, namely,

$$\begin{cases} A = r_P T_P^0 + r_S T_S^0 \\ B = r_P T_P^1 + r_S T_S^1 + r_{XP} T_{XP}^1 + r_X T_X^1 \\ C = r_{XP} T_{XP}^2 + r_X T_X^2 \\ D = T_P^0 + T_S^0 \\ E = T_P^1 + T_S^1 + T_{XP}^1 + T_X^1 \\ F = T_{XP}^2 + T_X^2, \end{cases} . \qquad [28]$$

Here, $T_Y^n [X]^n$ is the sum of spanning trees rooted in Y where n edges depend on $[X]$ participate. For example, $T_{XP}^1 = k_{SP}[P] k_{PXP} k_{XXP}[P] + k_{PS} k_{SX} k_{XXP}[P] + k_{XS} k_{SP}[P] k_{PXP}$ is the sum of all spanning trees rooted in state XP that carry a linear $[X]$-dependence. The other explicit expressions of the coefficients T_Y^n are visualized in Figure S7.

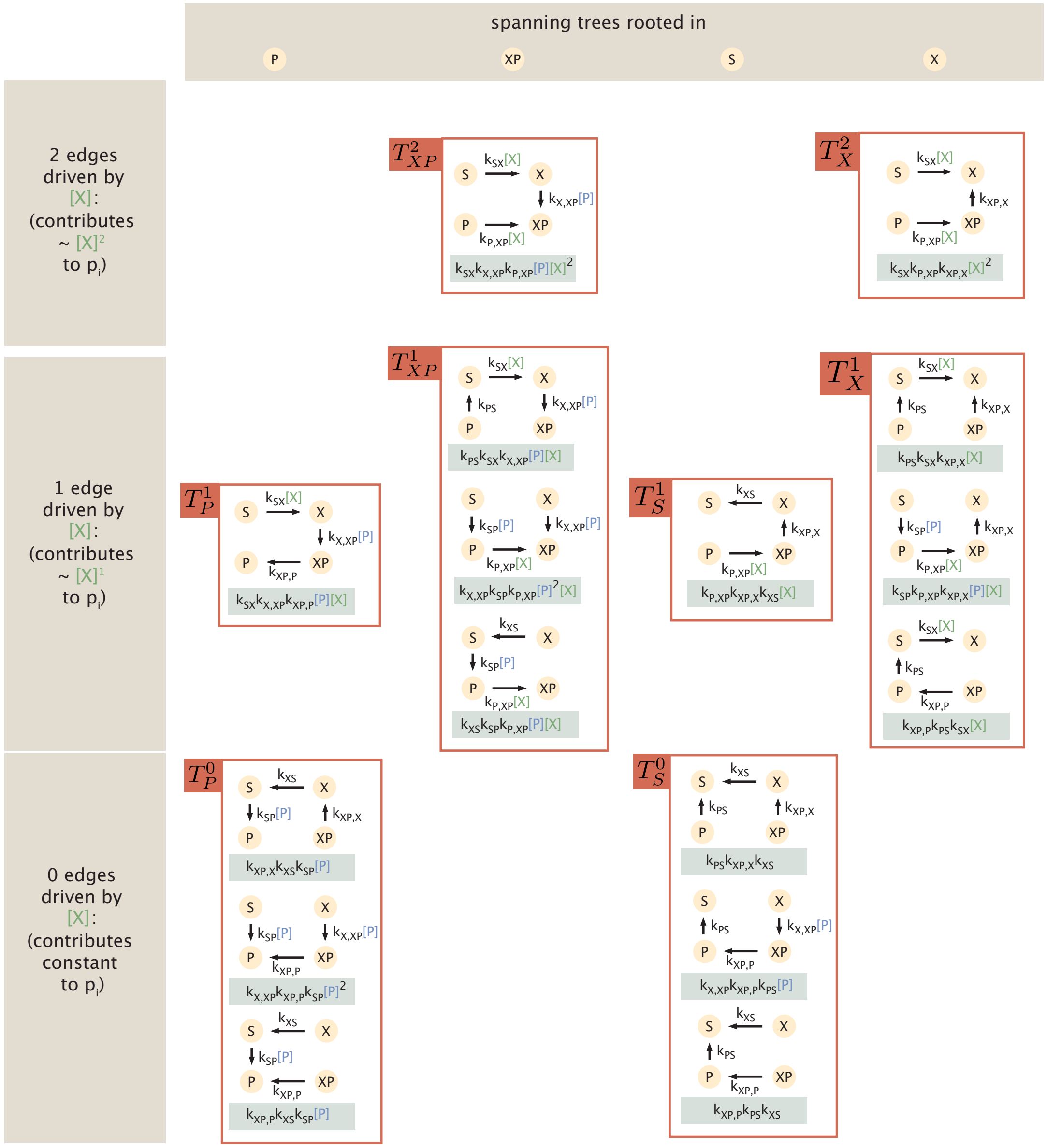

Fig. S7. All 16 rooted spanning trees of the four-state cycle can be classified by which node serves as the root (in columns) and the participating number of edges that contribute a power n of the transcription factor concentration $[X]$ (in row n). The weighted spanning trees completely determine the universal form of the fold-change output, as specified by Eqs.27 and 28.

E. Discussion on observable conventions: the logarithmic control variable. Throughout our analysis and discussion in this paper, we monitor the shape, number of inflection points, and sensitivity of transcriptional outputs with respect to the control parameter of the concentration of transcription factor, on a *logarithmic* scale. We use this logarithmic convention in alignment with common practice in biochemical and transcriptional studies (1, 45, 47). Using log concentration is convenient in the common setting where environmental inputs or governing transcription factor concentrations can vary over orders of magnitude, or where biochemical control systems are conceptually implementing a sort of fold-change detection (48).

This logarithmic convention is largely benign, since it is grounded in a monotonic one-to-one transformation of the control variable measured on a linear scale; however, it has two small mathematical consequences we briefly appraise. First, counting the number of inflection points with respect to the *logarithmic* control variable can introduce an additional point of inflection compared to the linear control variable. This occurs for the discussions of the shape of detailed balance responses,

$$\langle r \rangle^{\mathrm{eq}} = \frac{A' + B'[X]}{C' + D'[X]}. \qquad [29]$$

This is famously just a Langmuir binding curve or Hill function of order one, which on a linear scale is a hyperbola (nonsigmoidal and without any inflection points). However, it is quite common to depict such curves on a logarithmic scale, where the curve gains sigmoidal character and a point of inflection; the inflection point's local slope defines an effective Hill coefficient. This

canonical view, with respect to a logarithmic control variable, is the picture we invoke while counting inflection points or describing shapes.

Second, taking a logarithm invites a mathematical comment on units. Any logarithm of a concentration control variable must be understood as a logarithm of that concentration relative to some standard concentration scale, for instance 1 nanomolar. In plots where $\log[X]$ appears, the reference concentration merely denotes the horizontal offset/position of the curve. The particular choice of such a standard reference concentration scale $[X]_0$ has no effect on logarithmic derivatives, because of the simple fact that

$$\frac{df(x)}{d\log([X]/[X]_0)} = \frac{df(x)}{d(\log[X] - \log[X]_0)} = \frac{df(x)}{d\log[X]}. \quad [30]$$

F. Collapse of eight parameters into two emergent fundamental shape parameters (a, b). Now, by neglecting scales and shifts, we show how we can reduce the ratio of quadratic polynomials Eq. [28]—possessing six coefficients that are functions of eight rate constants—to an emergent form of just two shape parameters, namely:

$$\boxed{\frac{\langle r\rangle - \langle r\rangle_0}{\langle r\rangle_\infty - \langle r\rangle_0} = \frac{ax + x^2}{1 + bx + x^2}}, \quad [31]$$

where $\langle r\rangle_0$ and $\langle r\rangle_\infty$ are the leakiness and saturation of the function, expressible in terms of ratios of coefficients: $\lim_{[X]\to 0}\langle r\rangle = \langle r\rangle_0 = \frac{A}{D}$ and $\lim_{[X]\to\infty}\langle r\rangle \equiv \langle r\rangle_\infty = \frac{C}{F}$.

To show this two-parameter form of Eq. 31, we preview our procedure as follows. We divide by one of the six original coefficients of Eq. 27 (here, the coefficient D); extract an additive factor of the leakiness $\langle r\rangle_0$; nondimensionalize the concentration $[X]$ by a convenient concentration scale that emerges; perceive that a multiplicative factor of the dynamic range $\langle r\rangle_\infty - \langle r\rangle_0$ can be demanded to appear; and summarize the resulting expression by defining just two emergent shape parameters. To wit,

$$\begin{aligned}
\langle r\rangle &= \frac{A + B[X] + C[X]^2}{D + E[X] + F[X]^2} && [32]\\
&= \frac{\frac{A}{D} + \frac{B}{D}[X] + \frac{C}{D}[X]^2}{1 + \frac{E}{D}[X] + \frac{F}{D}[X]^2} && [33]\\
&= \langle r\rangle_0 + \frac{\frac{A}{D} + \frac{B}{D}[X] + \frac{C}{D}[X]^2 - \langle r\rangle_0(1 + \frac{E}{D}[X] + \frac{F}{D}[X]^2)}{1 + \frac{E}{D}[X] + \frac{F}{D}[X]^2} && [34]\\
&= \langle r\rangle_0 + \frac{(\frac{B}{D} - \langle r\rangle_0\frac{E}{D})[X] + (\frac{C}{D} - \langle r\rangle_0\frac{F}{D})[X]^2}{1 + \frac{E}{D}[X] + \frac{F}{D}[X]^2} && [35]
\end{aligned}$$

Now we nondimensionalize the control parameter by a convenient concentration scale, $[X]_0 = \sqrt{\frac{D}{F}}$, thus expressing the observable with respect to the rescaled concentration variable, $x \equiv \frac{[X]}{[X]_0}$:

$$\langle r\rangle = \langle r\rangle_0 + \frac{\frac{E}{\sqrt{DF}}(\frac{B}{E} - \langle r\rangle_0)x + (\frac{C}{F} - \langle r\rangle_0)x^2}{1 + \frac{E}{\sqrt{DF}}x + x^2} \quad [36]$$

As long as $\langle r\rangle_\infty \neq \langle r\rangle_0$, a condition we will consider shortly, we can rewrite this form of the observable as

$$\langle r\rangle = \langle r\rangle_0 + (\langle r\rangle_\infty - \langle r\rangle_0)\frac{\frac{E}{\sqrt{DF}}\frac{\frac{B}{E} - \langle r\rangle_0}{\langle r\rangle_\infty - \langle r\rangle_0}x + x^2}{1 + \frac{E}{\sqrt{DF}}x + x^2}. \quad [37]$$

Finally, this form invites us to define shape parameters a, b as

$$\begin{cases} b = \frac{E}{\sqrt{DF}} \\ a = b\frac{\frac{B}{E} - \langle r\rangle_0}{\langle r\rangle_\infty - \langle r\rangle_0}, \end{cases} \quad [38]$$

and allows us to write

$$\langle r\rangle = \langle r\rangle_0 + (\langle r\rangle_\infty - \langle r\rangle_0)\frac{ax + x^2}{1 + bx + x^2}, \quad [39]$$

recovering the simplified expression Eq. [31].

Now we return to address the assumption that $\langle r\rangle_0 \neq \langle r\rangle_\infty$, i.e. that the uninduced response (leakiness) is different from the maximally induced response (saturation). If instead we are in the unusual special case that the response does not change with $[X]$ at all, we extend a by continuity to $a = b$. In this constant case, $\frac{B}{E} - \langle r\rangle_0 = \langle r\rangle_\infty - \langle r\rangle_0$. In fact the function is constant when $\frac{B}{E} = \frac{A}{D} = \frac{C}{F}$ and the whole polynomial of order two factors out. Is this limit, $\frac{a}{b} \to 1$, and the form of Eq. [31] still holds.

Otherwise, if $\langle r\rangle_0 = \langle r\rangle_0$ but the function is *not* constant everywhere, a is infinite and the proper simplified parameterization of the observable instead becomes $\langle r\rangle = \langle r\rangle_0 + \frac{cx}{1+bx+x^2}$, with $c = b(\frac{B}{E} - \langle r\rangle_0)$. In this case, the function is non-monotonic. Indeed, the function has to both increase and decrease to have the same limit at zero and infinity without being constant. We do not make an elaborate quantitative study of this class of function, because we propose that in biological systems that succeed at accomplishing regulation, it is usually the case that the uninduced and maximally induced responses are at least infinitesimally different, namely $|\langle r\rangle_\infty - \langle r\rangle_0| = \epsilon$ with ϵ finite. However, philosophically, this type of eccentric response is still accommodated by the parameterization of Eq. [31] in the limit that $a \to \infty$.

G. Equilibrium responses of the square graph.

G.1. Demotion of responses to a (monotonic) ratio of linear polynomials at equilibrium. Here, we derive Eq. 3 of the main text (also reproduced here as Eq. [29]), that any observable produced by the square graph is demoted to a ratio of *linear* polynomials in $[X]$ at detailed balance. Informally, our strategy will be to factor out a statistical weight of a particular reference state from every statistical weight that participates in defining the observable $\langle r\rangle$; this forces ratios of statistical weights to appear, which the detailed balance condition can express in terms of ratios of rate constants. In the square graph, the ratios of rate constants can carry only a single power of $[X]$, motivating the appearance of linear terms only. (Along the quick mathematical journey, we will resolve the minor mathematical wrinkle that the detailed balance condition only comments immediately on the ratio of two statistical weights when those states are connected in the graph.)

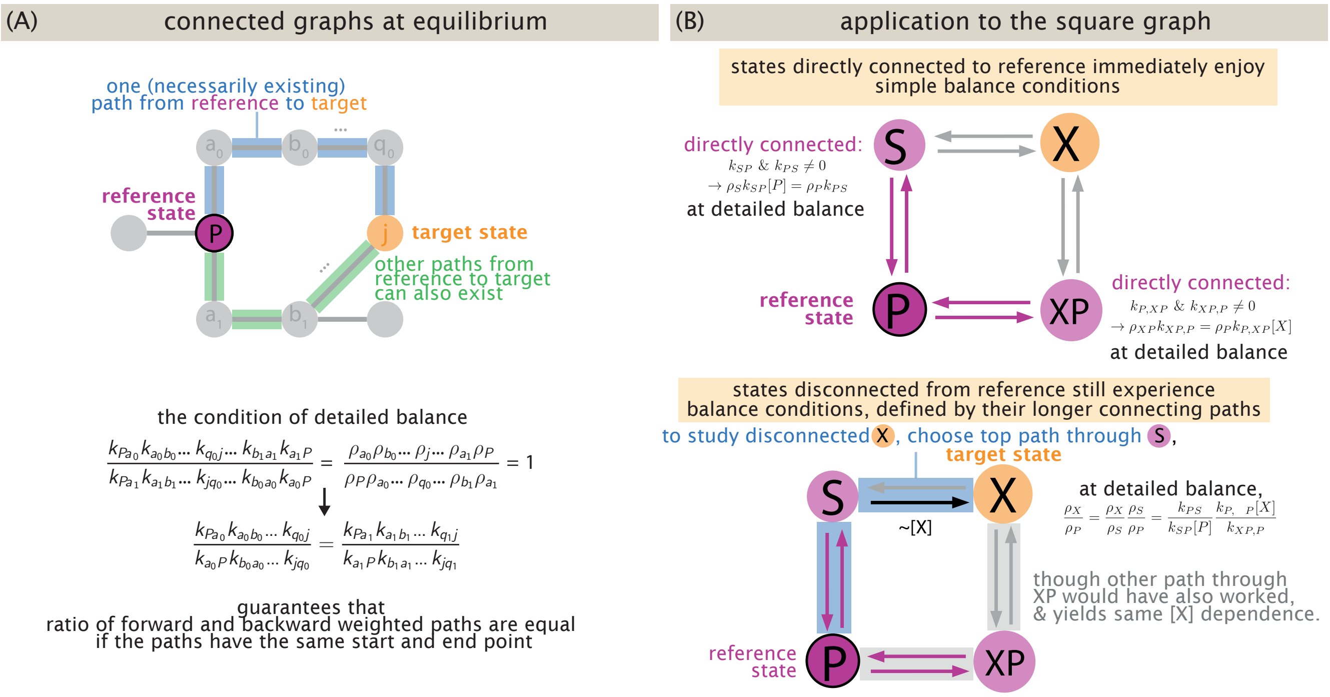

Fig. S8. Illustrating how the equilibrium output function of a graph can be described from paths between a target state and the other states of the graph. (A): In any connected graph, there is at least one path going from say a reference state (P) to a target state (q_0) (represented in blue). This path is not necessary unique; if another path exists between the reference state and the target state (represented in green), we can apply detailed balance and conclude that the ratios of forward and backward weighted green and blue paths are equal. (B): Expression of the weighted paths between a reference state and all the other states of the square graph. We can split these paths between paths to directly-connected-to-target states, which is a single edge, and paths to disconnected-from-target states, which consists in a collection of edges between adjacent states. In both cases we can apply detailed balance for each pair of edges.

We proceed. Choose the reference state to be state P, for concreteness though arbitrarily (as long as this reference state has

nonzero steady-state probability). We can write,

$$\langle r\rangle = \sum_i r_i p_i \tag{40}$$

$$= \frac{\sum_i r_i \rho_i}{\sum_i \rho_i} \tag{41}$$

$$= \frac{\rho_P \sum_i r_i \frac{\rho_i}{\rho_P}}{\rho_P \sum_i \frac{\rho_i}{\rho_P}} \tag{42}$$

$$= \frac{r_P + \sum_{\substack{\text{connected}\\ i\neq P}} r_i \frac{k_{Pi}}{k_{iP}} + \sum_{\substack{\textbf{dis}\text{connected}\\ j\neq P}} r_j \frac{\rho_j}{\rho_P}}{1 + \sum_{\substack{\text{connected}\\ i\neq P}} \frac{k_{Pi}}{k_{iP}} + \sum_{\substack{\textbf{dis}\text{connected}\\ j\neq P}} \frac{\rho_j}{\rho_P}} \tag{43}$$

Why does the last line have separated sums? This is the mathematical wrinkle we alluded to. Detailed balance guarantees that $\rho_i k_{iP} = \rho_P k_{Pi}$ for any state i. Normally, if the rates are nonzero, this suggests we can replace a ratio of statistical weights by a ratio of rate constants (the first sum). However, if a state j is *not* connected to P (namely $k_{jP} = k_{Pj} = 0$), then we can no longer necessarily write $\frac{\rho_j}{\rho_P}$ as a pure ratio of just two rate constants. We illustrate this separation in two cases in the right panel of Figure S8.

To make further progress, we consider the second sum in the numerator, whose summands are those ratios ρ_j/ρ_P for states j that are not connected to P. By the strongly-connected structural assumption that empowers us to apply the Matrix Tree Theorem, there must be at least one path (built from some number q of edges in the graph) that connects state j to state P. Hence, the ratio of statistical weights can be written as a product of rate ratios along that path, giving

$$\frac{\rho_j}{\rho_P} = \frac{\rho_j}{\rho_a}\frac{\rho_a}{\rho_b}\frac{\rho_b}{\rho_c}\cdots\frac{\rho_r}{\rho_q}\frac{\rho_q}{\rho_P} \tag{44}$$

$$= \underbrace{\frac{k_{aj}}{k_{ja}}\frac{k_{ba}}{k_{ab}}\frac{k_{cb}}{k_{bc}}\cdots\frac{k_{qr}}{k_{rq}}\frac{k_{Pq}}{k_{qP}}}_{q \text{ ratios}}. \tag{45}$$

Writing the ratio of these two equilibrium statistical weights in term of rate constants, allows us to write the functional of the output $\langle r\rangle$ with respect to the concentration of transcription factor. Indeed, for bi-molecular reactions, rates of binding of this transcription factor depend linearly on its concentration. We write $W_{j\to P}$ the weight of the path going from j to P. We are interested in the functional form of the output with respect to a set of variables $\{x_1, ..., x_n\}$ which are linearly involved in a set of edges E_x. That is to say, for an edge e in the set E_x, its weight is $w_e = k_e x_i$, with k_e a constant with respect to all $i \in [1, n]$. Let P be a chosen state in the studied graph, knowing the dependency of $\frac{W_{j\to P}}{W_{P\to j}}$ on the set $\{x_1, ..., x_n\}$ for every state j, then the form of the output at equilibrium is also known and written in Eq. [46]. We write F the ensemble of tuples $(i_1, ..., i_n)$, which correspond to the dependency on $(x_1, ..., x_n)$ of a ratio of paths from any state j of the graph to reference state P. This is illustrated in the left panel (A) of Figure S8.

$$\langle r\rangle_{eq} = \frac{\sum_{(i_1...i_n)\in F} a_{i_1...i_n} x_1^{i_1}...x_n^{i_n}}{\sum_{(i_1...i_n)\in F} b_{i_1...i_n} x_1^{i_1}...x_n^{i_n}} \tag{46}$$

The coefficients $(a_{i_1,...,i_n}, b_{i_1,...,i_n})$ are constant and non negative . This reasoning is possible, because we study cases where a variation in any of the variables $\{x_1, ..., x_n\}$, doesn't break detailed balance. Therefore, the choice of the path taken between any node j of the graph and P doesn't have any influence on the result because, the weight of a cycle in one direction should be equal to the weight of the cycle in the other direction. Indeed, applying the detailed balance condition along all of the edges of the cycle, proves this remark. So if two paths have the same endpoint, they form a loop and we can apply the previous statement (49). In other terms, let's consider a path $L_1 := P \to a_0 \to b_0 \to ... \to q_0 \to j$ from P to j and we suppose that there exists another path from P to j $L_2 := P \to a_1 \to b_1 \to ... \to j$. We call L_{-1} and L_{-2} the path associated respectively to L_1 and L_2 but in the other direction that is to say from j to P. Moreover W_L is weight of a path L. We notice that $L_1 \cup L_{-2}$ and $L_2 \cup L_{-1}$ form cycles in opposite directions, at equilibrium $\dfrac{W_{L_1}W_{L_{-2}}}{W_{L_{-1}}W_{L_2}} = \dfrac{k_{Pa_0}k_{a_0b_0}...k_{q_0j}...k_{b_1a_1}k_{a_1P}}{k_{Pa_1}k_{a_1b_1}...k_{jq_0}...k_{b_0a_0}k_{a_0P}} = \dfrac{\rho_{a_0}\rho_{b_0}...\rho_j...\rho_{a_1}\rho_P}{\rho_P\rho_{a_0}...\rho_{q_0}...\rho_{b_1}\rho_{a_1}} = 1$. So $\dfrac{W_{L_1}}{W_{L_{-1}}} = \dfrac{W_{L_2}}{W_{L_{-2}}}$.

Returning to the specifics of the four-state graph and our reference state P, we see that states S and XP are both connected to P, giving the first, connected-state sum as the equation bellow.

$$\sum_{\substack{\text{connected}\\ i\neq P}} r_i \frac{k_{Pi}}{k_{iP}} = r_S \frac{k_{PS}}{k_{SP}[P]} + r_{XP}\frac{k_{PXP}[X]}{k_{XPP}} \qquad [47]$$

The only state that is disconnected from state P, giving the disconnected sum, is state X. Without loss of generality, we now rewrite $\frac{\rho_X}{\rho_P}$ using the path of edges that goes through S. (We recover the same ultimate $[X]$-dependency if we had chosen the path through XP instead.) This gives,

$$\frac{\rho_X}{\rho_P} = \frac{\rho_X}{\rho_S}\frac{\rho_S}{\rho_P} \qquad [48]$$

$$= \frac{k_{SX}[X]}{k_{XS}}\frac{k_{PS}}{k_{SP}[P]}. \qquad [49]$$

So the disconnected sum is just $\sum_{\substack{\textbf{dis}\text{connected}\\ j\neq P}} r_j \frac{\rho_j}{\rho_P} = r_X \frac{k_{SX}[X]}{k_{XS}}\frac{k_{PS}}{k_{SP}[P]}$. Altogether, we recover

$$\langle r\rangle^{\text{eq.}} = \frac{r_P + \left(r_S \frac{k_{PS}}{k_{SP}[P]} + r_{XP}\frac{k_{PXP}[X]}{k_{XPP}}\right) + \left(r_X \frac{k_{SX}[X]}{k_{XS}}\frac{k_{PS}}{k_{SP}[P]}\right)}{1 + \left(\frac{k_{PS}}{k_{SP}[P]} + \frac{k_{PXP}[X]}{k_{XPP}}\right) + \left(\frac{k_{SX}[X]}{k_{XS}}\frac{k_{PS}}{k_{SP}[P]}\right)} \qquad [50]$$

$$:= \boxed{\frac{A' + B'[X]}{C' + D'[X]}}, \qquad [51]$$

where we have highlighted how both the numerator and denominator admit only up to a linear dependence on $[X]$, and A', B', C', D' are coefficients that depend only on weighted ratios of opposing rate constants (and are hence set fully thermodynamically by energy parameters).

The reasoning above suggests that the fact that every path connecting two states contains at most one power of $[X]$ was a crucial architectural ingredient for the collapse of the ratio of quadratic polynomials to a ratio of linear polynomials in the square graph. One interesting transparent consequence this reasoning highlights is that the same collapse (to a ratio of linear polynomials at detailed balance) must occur for the completely-connected graph.

REMARK. The reasoning above is one way to establish (or alternatively, itself follows from) the celebrated path-independence of ratios of detailed-balance probabilities (49, Eq. 7).

G.2. Leakiness, saturation, and EC50 are tunable at equilibrium. As mentioned in the main text, the response's leakiness (value when $[X]$ is completely absent) and saturation (value when $[X]\to\infty$) are set by the fact that the four state graph collapses into a different two-state linear graph for each limit. Specifically, the kinetics reduce to,

$$\begin{cases} [X]\to 0: & S \underset{k_{PS}}{\overset{k_{SP}[P]}{\rightleftharpoons}} P \\ [X]\to\infty: & X \underset{k_{XP,X}}{\overset{k_{X,XP}[P]}{\rightleftharpoons}} XP \end{cases}. \qquad [52]$$

Since these two-state truncated graphs are linear, and so must be at equilibrium, we observe that the values of the leakiness and saturation must be thermodynamic statistical averages of the r_i. We conclude that

$$\begin{cases} \langle r\rangle_0 = r_P p_P + r_S\,(1-p_P) \\ \langle r\rangle_\infty = r_{XP} p_{XP} + r_X\,(1-p_{XP}), \end{cases} \qquad [53]$$

where

$$\begin{cases} p_P = \frac{k_{SP}[P]}{k_{SP}[P]+k_{PS}} \equiv \frac{1}{1+e^{-\beta\Delta\epsilon_{SP}}} \\ \\ p_{XP} = \frac{k_{X,XP}[P]}{k_{X,XP}[P]+k_{XP,X}} \equiv \frac{1}{1+e^{-\beta\Delta\epsilon_{XXP}}}, \end{cases} \qquad [54]$$

are the simple stationary-solutions of each two-state system, and where we have defined the appropriate Boltzmann energy parameters via each ratio of rates. Indeed, we can express the probabilities of being in one of two state system at equilibrium solving for the Lagrangian matrix of the Markov system, and then the correspondence between the kinetic rates and the energies through the equilibrium constants Eq. [23]. Hence leakiness and saturation are controllable by thermodynamic means.

Further assessing the form of the inflection point when the observable is at detailed balance reveals that it can be set by another ratio of rates, hence energy parameter. However, the raw sharpness at the inflection point remains equal to one fourth of the dynamic range. We demonstrate this obligatory proportionality between the maximum raw sharpness and dynamic

range as follows. At equilibrium, taking one derivative of the detailed balance response described by Eq. [51] gives the raw sharpness as,

$$\frac{d\langle r\rangle^{\text{eq.}}}{d(\ln[X]/[X]_0)} = \frac{(B'C' - A'D')[X]}{(C' + D'[X])^2}. \quad [55]$$

Taking an additional derivative to solve for the inflection point where $\frac{d^2\langle r\rangle^{\text{eq.}}}{d(\ln[X]/[X]_0)^2} = 0$ gives,

$$\frac{d^2\langle r\rangle^{\text{eq.}}}{d(\ln[X]/[X]_0)^2} = \frac{(B'C' - A'D')(C' - D'[X])[X]}{C' + D'[X]}. \quad [56]$$

The inflection point, where this second derivative vanishes and the raw sharpness is maximized, occurs at $[X]_* = C'/D'$. Substituting this into the maximal sharpness expression, we find the maximum sharpness at equilibrium is merely

$$\max \frac{d\langle r\rangle^{\text{eq.}}}{d(\ln[X]/[X]_0)} = \frac{1}{4}\left(\frac{B'}{D'} - \frac{A'}{C'}\right). \quad [57]$$

Now, note that the equilibrium leakiness is given by

$$\langle r\rangle_0^{\text{eq}} \equiv \lim_{[X]\to 0} \langle r\rangle^{\text{eq}} = \frac{A'}{C'}, \quad [58]$$

and the saturation is given by

$$\langle r\rangle_\infty^{\text{eq}} \equiv \lim_{[X]\to \infty} \langle r\rangle^{\text{eq}} = \frac{B'}{D'}, \quad [59]$$

so the maximum sharpness is indeed one fourth the dynamic range,

$$\max \frac{d\langle r\rangle^{\text{eq.}}}{d(\ln[X]/[X]_0)} = \frac{1}{4}\left(\langle r\rangle_\infty^{\text{eq}} - \langle r\rangle_0^{\text{eq}}\right). \quad [60]$$

These constrained behaviors of the equilibrium response are summarized in Figure S9.

A transcription factor is a global, overall repressor when the saturation is smaller than the leakiness, $\langle r\rangle_\infty < \langle r\rangle_0$. Conversely, a transcription factor is overall an activator when the saturation is larger than the leakiness, $\langle r\rangle_\infty > \langle r\rangle_0$. As we have just seen, since the leakiness and saturation are set thermodynamically, so too is the global nature of the transcription factor as an overall repressor or activator.

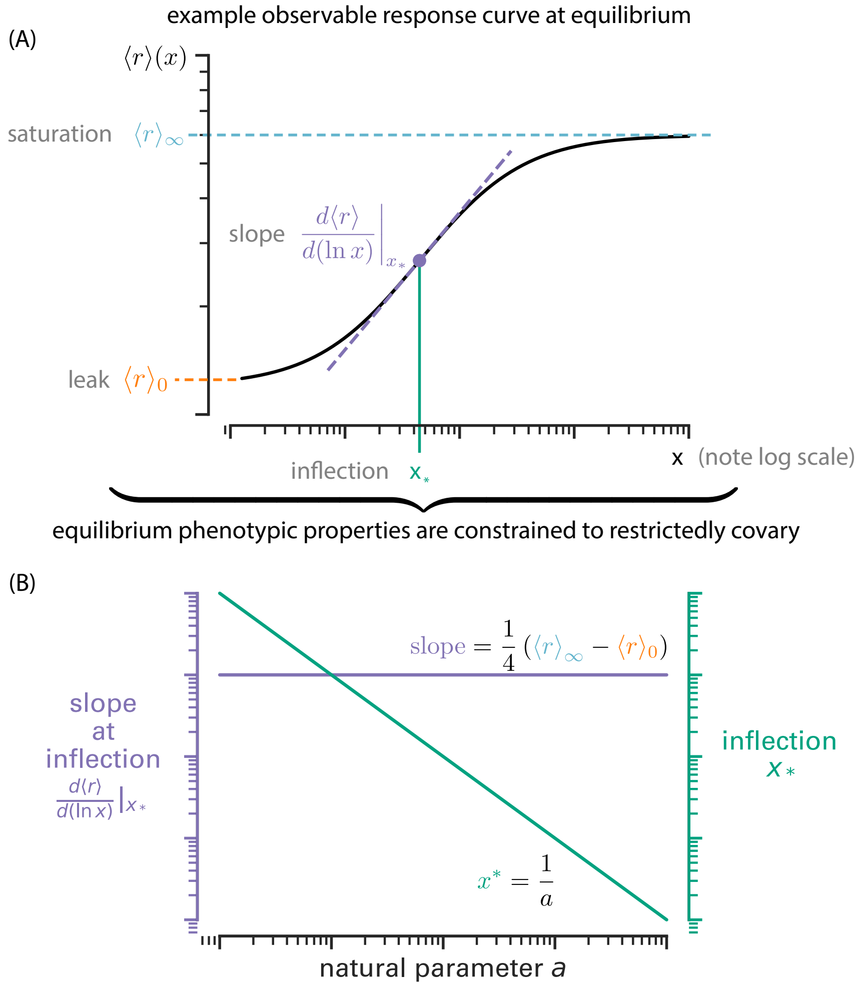

Fig. S9. Response curves at equilibrium. (A): At equilibrium, response curves are always monotonic in the control variable x, with (at most) one inflection point in a log scale with respect to x. The leak (observable at zero x, $\langle r \rangle_0$, in orange); location x_* of the inflection point (in green); slope at the inflection (in purple); and saturation limit (in pale blue) capture the properties of the curve. (B): Equilibrium imposes the constraint that these phenotypic properties vary in fixed relationships.

G.3. Validating consilience between kinetic and thermodynamic viewpoints. To be helpful to the reader interested in reconciling thermodynamic models, experimental parameters such as equilibrium dissociation constants that may parameterize them, and the more elaborate kinetic parameterization of continuous-time Markov chains and the Matrix Tree Theorem, below we endeavor a parameter-by-parameter correspondence between these viewpoints. This correspondence is valid when energy dissipation vanishes.

From a kinetic viewpoint, detailed balance implies that the ratio of two states is expressible as a ratio of rate constants. From a thermodynamic viewpoint, the same ratio of two states is expressible as a ratio of Boltzmann weights set by thermodynamic energy parameters. To link these perspectives, we define an effective equilibrium dissociation constant between a molecule Y and a site H, where the site can either be completely empty or also occupied by another molecule in its vicinity. We denote these dissociationequilibrium constants K_{HY} and largely following the conventions discussed in Ref. (36), define them as

$$K_{HY} = \frac{[Y]}{y^H}, \tag{61}$$

where $y^H = \frac{\rho_{HY}}{\rho_H}$ is a ratio of statistical weights. Specifically, ρ_{HY} is statistical weight of the molecule Y bound to the site H, and ρ_H is the statistical weight of the state where the molecule Y is not bound to the site H. With this definition, the ratio of probabilities of two states is constant and the dissociation constant has units of a concentration.

For the square graph of four states—namely when the site is empty (S), when the transcription factor is bound to the DNA (X), when the polymerase is bound to the DNA (P), and when both the transcription factor and the polymerase are both

bound to the DNA (XP)—we can define the effective equilibrium dissociation constants explicitly, seeing,

$$\begin{cases} K_{SP} = \dfrac{[P]}{p^S} = \dfrac{[P]\rho_S}{\rho_P} \\ K_{XP} = \dfrac{[X]}{x^P} = \dfrac{[X]\rho_P}{\rho_{XP}} \\ K_{XP} = \dfrac{[P]}{p^X} = \dfrac{[P]\rho_X}{\rho_{XP}} \\ K_{SX} = \dfrac{[X]}{x^S} = \dfrac{[X]\rho_S}{\rho_X}. \end{cases} \quad [62]$$

In the 4-state-graph, detailed balance implies that,

$$\begin{cases} \dfrac{\rho_X}{\rho_S} = \dfrac{[X]k_{SX}}{k_{XS}} \\ \dfrac{\rho_P}{\rho_S} = \dfrac{[P]k_{SP}}{k_{PS}} \\ \dfrac{\rho_X}{\rho_{XP}} = \dfrac{k_{XP,X}}{[P]k_{XP,X}} \\ \dfrac{\rho_P}{\rho_{XP}} = \dfrac{k_{XP,P}}{[X]k_{XP,P}}. \end{cases} \quad [63]$$

So we can express the effective equilibrium dissociation constants as functions of rate constants, recovering,

$$\begin{cases} K_{SP} = \dfrac{k_{PS}}{k_{SP}} \\ K_{XP} = \dfrac{k_{XP,P}}{k_{XP,P}} \\ K_{XP} = \dfrac{k_{XP,X}}{k_{XP,X}} \\ K_{SX} = \dfrac{k_{XS}}{k_{SX}}. \end{cases} \quad [64]$$

Similarly, we can derive their expression with the thermodynamic formalism. Referring to Reference (36), we can define the partition function of the 4 states characterising the simple activation as follows,

$$Z(P,X) = \frac{N!}{P!X!(N-P-X)!} e^{-P\beta\epsilon_{pd}^{ns} - X\beta\epsilon_{xd}^{ns}}, \quad [65]$$

where $\beta = 1/k_B T$, k_B is the Boltzmann constant,T the temperature and N the number of non specific binding sites. For the transcription case we can define $\Delta\epsilon_{yd} = \epsilon_{yd}^{s} - \epsilon_{yd}^{ns}$, where ϵ_{yd}^{s} is the energy of the molecule Y being on a specific site and ϵ_{yd}^{ns} the energy of the molecule being on a non specific site and ϵ_{xp} the interaction energy between the transcription factor and the polymerase. X and P are respectively the copy number of transcription factor and polymerase. N is the number of nonspecific binding sites. We can define the weights of the different nodes at thermodynamic equilibrium (36),

$$\begin{cases} \rho_S = Z(P,X) \\ \rho_P = Z(P-1,X)e^{-\beta\epsilon_{pd}^{s}} \\ \rho_{XP} = Z(P-1,X-1)e^{-\beta(\epsilon_{pd}^{s} + \epsilon_{xd}^{s} + \epsilon_{px})} \\ \rho_X = Z(P,X-1)e^{-\beta\epsilon_{xd}^{s}}. \end{cases} \quad [66]$$

Using the statistical mechanics approximation ($N \gg P, X$), we compute the effective equilibrium dissociation constants,

$$\begin{cases} K_{SP} = [P]\dfrac{N}{P}e^{\beta\Delta\epsilon_{pd}} \\ K_{XP} = [X]\dfrac{N}{X}e^{\beta(\Delta\epsilon_{xd} + \epsilon_{xp})} \\ K_{XP,X} = [P]\dfrac{N}{P}e^{\beta(\Delta\epsilon_{pd} + \epsilon_{xp})} \\ K_{SX} = [X]\dfrac{N}{X}e^{\beta\Delta\epsilon_{xd}}. \end{cases} \quad [67]$$

We can note that $[X] = \frac{X}{N_A V_{cell}}$ and $[P] = \frac{P}{N_A V_{cell}}$. This then simplifies to Eq. [68], which give the expression of this dissociation constants in both the kinetic and thermodynamic viewpoints as,

$$\begin{cases} K_{SP} = \dfrac{k_{PS}}{k_{SP}} = C_N e^{\beta\Delta\epsilon_{pd}} \\ K_{XP} = \dfrac{k_{XP,P}}{k_{XP,P}} = C_N e^{\beta(\Delta\epsilon_{xd}+\epsilon_{xp})} \\ K_{XP} = \dfrac{k_{XP,X}}{k_{XP,X}} = C_N e^{\beta(\Delta\epsilon_{pd}+\epsilon_{xp})} \\ K_{SX} = \dfrac{k_{XS}}{k_{SX}} = C_N e^{\beta\Delta\epsilon_{xd}}, \end{cases} \quad [68]$$

where $C_N = \frac{N}{N_A V_{cell}}$ is the molar concentration of non-specific sites in the cell.

Let us express the probability of the polymerase being bound to the DNA. First, we may write $p_P = \frac{\rho_P}{\rho_P+\rho_S+\rho_{XP}+\rho_X}$ and $p_{XP} = \frac{\rho_{XP}}{\rho_P+\rho_S+\rho_{XP}+\rho_X}$. Then, we may write,

$$p_{bound} = p_P + p_{XP} = \frac{1+\frac{\rho_{XP}}{\rho_P}}{1+\frac{\rho_{XP}}{\rho_P}+\frac{\rho_X}{\rho_P}+\frac{\rho_S}{\rho_P}} = \frac{1+\frac{[X]}{K_{XP}}}{1+\frac{[X]}{K_{XP}}+\frac{[X]K_{SP}}{[P]K_{SX}}+\frac{K_{SP}}{[P]}}, \text{ and,}$$

$$p_{bound} = \frac{1+\frac{[X]}{K_{XP}}}{1+\frac{K_{SP}}{[P]}+[X](\frac{1}{K_{XP}}+\frac{K_{SP}}{[P]K_{SX}})}. \quad [69]$$

We can express this probability in terms of kinetic rate constants and concentrations as,

$$p_{bound} = \frac{1+\frac{[X]k_{XP,P}}{k_{XP,P}}}{1+\frac{k_{PS}}{k_{SP}[P]}+[X](\frac{k_{XP,P}}{k_{XP,P}}+\frac{k_{PS}k_{XS}}{[P]k_{SP}k_{SX}})}. \quad [70]$$

Alternatively, we can also write this probability in term of energies and number of sites as:

$$p_{bound} = \frac{1+Xe^{-\beta(\Delta\epsilon_{xd}+\epsilon_{xp})}}{1+\frac{e^{\beta\Delta\epsilon_{pd}}}{P}+Xe^{-\beta\Delta\epsilon_{xd}}(e^{\beta\epsilon_{xp}}+\frac{e^{\beta\Delta\epsilon_{pd}}}{P})}. \quad [71]$$

We note that $X = \frac{[X]}{C_N}$ and $P = \frac{[P]}{C_N}$. These two expressions are equivalent.

G.4. Detailed balance is implied by $\gamma = 1$ and steady-state. To give concreteness to the general cycle condition we discussed in §C, we return to illustrate this result using the specific parameters of the square graph and a different, perhaps more transparent, algebraic tact.

Why is detailed balance—as expressed in Eq. [63]—equivalent to having a graph at steady state (where the Matrix Tree Theorem applies) and enforcing the cycle condition that the ratio of products of rate constants γ is unity? In the square graph, this cycle condition of unity is

$$\gamma \equiv \frac{\gamma_+}{\gamma_-} = \frac{k_{SX}k_{X,XP}k_{XP,P}k_{PS}[X][P]}{k_{XS}k_{XP,X}k_{P,XP}k_{SP}[X][P]} = \frac{k_{SX}k_{X,XP}k_{XP,P}k_{PS}}{k_{XS}k_{XP,X}k_{P,XP}k_{SP}} := 1. \quad [72]$$

First, define $\gamma_+ \equiv k_{SX}k_{X,XP}k_{XP,P}k_{PS}[X][P]$ and $\gamma_- \equiv k_{XS}k_{XP,X}k_{P,XP}k_{SP}[X][P]$, respectively, as the products of rate constant in the + (clockwise) and - (counterclockwise) directions.

We will now prove that at steady state, we can write:

$$\begin{cases} \rho_S k_{SX}[X] - \gamma_+ = \rho_X k_{XS} - \gamma_- \\ \rho_X k_{X,XP}[P] - \gamma_+ = \rho_{XP}k_{XP,P} - \gamma_- \\ \rho_{XP}k_{XP,P} - \gamma_+ = \rho_P k_{P,XP}[X] - \gamma_- \\ \rho_P k_{PS} - \gamma_+ = \rho_S k_{SP}[P] - \gamma_-. \end{cases} \quad [73]$$

This Eq. 73 suffices to show that when $\gamma_+ = \gamma_-$—which guarantees $\gamma = 1$, the cycle condition that ensures equilibrium—the gamma terms cancel, and we recover the equations Eq. [63] that define detailed balance.

To demonstrate the system of equations Eq. [73], we invoke the Matrix Tree Theorem. To illustrate the proof, we discuss just the first equation; the rest follow analogously. Specifically, we can write the statistical weights for the states X and S by applying the Matrix Tree Theorem, seeing that

$$\begin{cases} \rho_S = [X]k_{XS}k_{XP,X}k_{P,XP} + k_{XS}k_{XP,X}k_{PS} + k_{XS}k_{XP,P}k_{PS} + k_{X,XP}k_{XP,P}k_{PS}[P] \\ \rho_X = [X]^2k_{XP,X}k_{SX}k_{P,XP} + [X]k_{XP,X}k_{SX}k_{PS} + [X]k_{XP,P}k_{SX}k_{PS} + [X]k_{XP,X}k_{SP}k_{P,XP}[P] \end{cases} \quad [74]$$

Then, we multiply by $k_{SX}[X]$ the first line of Eq. [74] and by k_{XS} the second line of Eq. [74],

$$\begin{cases} \rho_S k_{SX}[X] = k_{XS}k_{XP,X}[X]k_{P,XP}k_{SX}[X] + k_{XS}k_{XP,X}k_{PS}k_{SX}[X] + k_{XS}k_{XP,P}k_{PS}k_{SX}[X] + k_{X,XP}k_{XP,P}k_{PS}[P]k_{SX}[X] \\ \rho_X k_{XS} = k_{XS}k_{XP,X}[X]k_{P,XP}k_{SX}[X] + k_{XS}k_{XP,X}k_{PS}k_{SX}[X] + k_{XS}k_{XP,P}k_{PS}k_{SX}[X] + [X]k_{XP,X}k_{SP}k_{P,XP}[P]k_{XS}. \end{cases} \quad [75]$$

In red, we recognize γ_+ and in orange γ_-; the rest of the expressions in blue are equal; and we recover the first equation of Eq. [73], as desired.

G.5. The cycle condition implies that changing transcription factor or polymerase concentrations does not affect the extent of disequilibrium in the square graph. Note that Eq. [72] demonstrates that because $[X]$ and $[P]$ appear in both the products of rates in the clockwise and counterclockwise directions, their influence on the value of γ cancels out. This means that adjusting the concentration of transcription factor or polymerase maintains the extent of disequilibrium or equilibrium exhibited by the system.

G.6. Connection and contrast with earlier results on kinetic cycles or square-graph analogs. Here, we briefly comment on some insightful, earlier work in different contexts that identified how some analogous architectures to our transcriptional square graph can behave differently in or out of equilibrium, drawing specific connections and contrasts with our results and analyses.

This aims to provide the interested reader an entry into the vast literature with some important and fundamental examples. Of course, this short list leaves off many important contributions. Our survey of the literature below attempts to explain the ways in which our work differs from that of other interesting and important advances. Naturally, the language we use sometimes includes phrases such as other works "did not do X." It is important to realize that often the reason these others did not do X, is because that is not what they set out to do. As a result, we do not say these words as a point of critique at all. Rather, it is just to try and clarify how these efforts and our own might differ.

- Writing in 1976, **J. Schnakenberg** (50) established how systems described by linear master equations can be treated by network representations amenable to graph theory, giving powerful and concise calculations of generalized fluxes and forces. Among Schnakenberg's primary examples is a kinetic cycle of N states. Being concerned with other important aims, Schnakenberg's expressions are generic and do not specify the order of rates in control variables such as concentrations, nor analyze transcriptional settings like the aim of our work.

- Originally published in 1989, **Terrell Hill**'s *Free Energy Transduction and Biochemical Kinetic Cycle Kinetics* (24) is a foundational, classic monograph, full of both pedagogical and pioneering insight. Hill does not explicitly use the graph theoretic terminology "spanning tree" or connect to matrix algebra, but accessibly introduces and illustrates the same precisely equivalent diagrammatic procedure on key biochemical examples. Two explicit examples for these techniques were computing steady-state probabilities and driving forces for a triangular cycle of three states (plus reasoning about a square cycle of four states for other calculational purposes about fluxes). Hill calculates the algebraic solution of each of the three states in the triangular cycle in terms of the six abstract arbitrary transition rate constants of the triangular graph (see (24, p. 45-46, Eqs. 6.10-6.12, Fig. 2.5)). Since his discussion was broadly illustrative of the general technique, Hill's example did not examine how the nature of these solutions changes in or out of equilibrium with respect to a specific biological control parameter, nor consider any transcriptional analogs with their attendant biochemical details like we aimed to in this present work.

- **Hong Qian**'s body of work is profound and merits broad awareness. Qian has carefully applied kinetic treatments to nonequilibrium steady-state behaviors across biological contexts, focusing on biochemical driving forces and the structure of resulting fluxes.

  - One excellent representative study is (51), which discusses a variety of case studies including kinetic cycles accomplished by single enzymes. Among the case studies in this work, similarly to Hill's example, are triangular kinetic schemes (e.g. (51, Fig. 1b, Fig. 5)) where some of the six transition rates among the three nodes are given linear dependencies on chemical concentrations. Qian finds nonequilibrium steady-state fluxes (e.g., reproducing Michaelis-Menten kinetic dependencies from a cycle scheme) and finds kinetic expressions for proofreading ratios. This work had other principal goals and thus did not consider the nature of curve shapes in or out of equilibrium; the behavior of a square graph; or transcriptional regulation.

  - Another of Qian's insightful works is (5), which focuses on how free energy couples to phosphorylation and dephosphorylation crcuits in the cell, and proofreading. Here Qian maps interesting performance measures such as (ultra)sensitivity of switches and proofreading ratios under drive. A specfic square graph architecture, with particular GTP- and GDP- dependent transition rates, enters Qian's discussion, but this is not analyzed algebraically as an input-output curve with respect to a control parameter; regarded with other choices of edges along which detailed balance could be broken; or connected to transcription.

- **Jeremy Gunawardena** and coworkers are truly foundational modern investigators and earn responsibility for demonstrating the formidable power that the diagrammatic framework of the Matrix Tree Theorem can deploy on real concrete problems across contemporary systems biology. Their insights appear across many beautiful papers, but especially relevant are the following references:

  - Mirzaev and Gunawardena (52) illustrate the "linear framework" in practice, where parts of a system that have rapidly operating internal degrees of freedom can be eliminated to yield a concise network whose Laplacian can be treated by the Matrix Tree Theorem. Their illuminating examples include kinetic cycles, analyzed largely with transition rates that lack specified dependencies on governing chemical concentrations (with the exception of their demonstration of Michaelis Menten kinetics).

– Estrada, Wong, DePace and Gunawardena (53) creatively map out how nonequilibrium escapes from the constraints of a "Hopfield barrier" in the sensitivity of a regulatory setting. Close in spirit to our study of gene regulation, this setting specifically considers individual transcription factor binding to sites, and focuses on mapping the set of maximal effective Hill coefficients/normalized slopes attainable by some architectures with and without energy expenditure. However, our work focuses on a plethora of different questions and highlights an alternative, complementary view: in addition to considering a pointwise slope, mapping the global *shape* of a response curve can itself be a revealing signature of nonequilibrium operation of a network. In addition, our work systematically considers the effect of breaking detailed balance edge-by-edge near plausible rate constants; derives new required rate imbalances to reach specific curve shapes; and other questions beyond.

– Nam, Martinez-Coral, and Gunawardena (54) survey implications of the "linear framework" for a number of case studies, especially enzyme kinetics. In one illustration, they consider an architecture explicitly very similar to our square graph (54, Fig. 3b), representing a biomolecule capable of being in two states orthogonal to whether it is bound to a ligand present at concentration x (ultimately creating four states). This work further derives algebraic expressions ((54, Eqs. 4.7 & 4.8)) for the steady-state probability of some states in or out of equilibrium in terms of x, which are near analogs to our Eq. 1 and Eq. 2 in the main text. The authors remark, "Even for the very simple example in figure 3b, the difference between equations (4.7) and (4.8) is striking in both rational structure and parametric complexity." Yet this work did not focus on or conduct analysis on the functional or biological implications of these algebraic differences in or out of equilibrium, nor do they specialize to details of transcription that would lead to our subsequent associated analyses.

- In an interesting work (12), **Hartich, Barato, and Seifert** carefully study a model of a receptor with four states (two bound with a ligand), structurally similar to our square graph. They monitor how maximal pointwise sensitivity varies with the ligand concentration; they also map how energy investment along one specific edge changes sensitivity. However, given its other rich goals, this work does not consider explicit enlargement of the space of mathematical behavior in or out of equilibrium in terms of curve shapes, nor does the work confront how breaking detailed balance along different edges changes the character of the response. In addition, the paper does not make contact with transcriptional questions.

H. Driving different arrows in the square graph can still yield a ratio of quadratic polynomials. Throughout this article, we study the response observable relative to the concentration of transcription factor $[X]$, tuning the edges in green in our square graph as visualized in Figure 1 of the main text. However, depending on the observable and the graph's architecture, the parameter controlling the observable could be different than this transcription factor. For instance, in different biological settings, two rate constants could be adjusted simultaneously by the same scalar control parameter if they are driven by the concentration of a different external (like ATP) or internal (like the polymerase P) molecule governing the system. Therefore, we can ask: for what classes of control parameter will the observable $\langle r \rangle$ exhibit the same functional form of a ratio of quadratic polynomials?

The Matrix Tree Theorem gives a precise structural answer to this question: when the graph has at least one rooted spanning tree with each of zero, one, and two edges that depend on the control parameter, the observable will inherit such a familiar quadratic dependence. This is a broad class of graphs. We now show some of the diversity of these graphs, whose response shapes and sensitivity bounds are necessarily mathematically identical to those we establish in the first half of the paper, by giving a few concrete examples of related graphs. (Further explicit examples of biological relevance (e.g. subsets of hypercubic state spaces created by mutually-exclusive transcription factor binding) are also discussed in this SI, §3.)

Figure S10A illustrates various graphs whose responses are mappable to that of our original square graph (itself illustrated in S10A(i)). The response's form is unchanged when we create a new graph by vertically reflecting the original graph (as in Fig. S10A(ii)), or merely rotating it (not displayed).

Another structurally-distinct but mathematically-equivalent type of graph is shown in Fig. S10A(iii) (also representing any other graph with two controlled edges that may be mapped by reflection or rotation onto the indicated red edges in Fig. S10A(iii)). To understand why this graph has the same quadratic dependence, we can refer to the spanning trees of the square graph using our original rate labels; these spanning trees include $k_{SX}[X]k_{XP,P}k_{X,XP}$ and $k_{SX}[X]k_{XP,P}k_{PS}$, which are both proportional to $k_{SX}k_{XP,P}$, namely both transitions in red imagined to be controlled by the common control variable in Fig. S10A(iii).

Figure S10A(iv) gives another graph where the red indicated arrows both participate in a common spanning tree, assuring the same quadratic dependence of interest. To see this fact, take the two indicated edges and add either the edge $k_{XP,P}$ or the edge $k_{XP,X}$; the results are both valid spanning trees rooted in S. Rotating this set of edges also generates three other equivalent graphs with the same behavior (not shown). (One minor difference between the observable produced by this type of graph is that when $[X] \to \infty$, the limit of this graph's observable is now constrained to 1, since the leading order spanning trees in the control parameter are rooted in the same node.)

Last, Fig. S10A(v) acknowledges that many other graphs with a larger set of nodes than four can exhibit the same quadratic form. As just one example, when there are only two controlled (red) transitions localized among some states in a suitable subgraph, all spanning trees of the larger graph can inherit the structural requirements imposed by the subgraph.

Of course, many graphs will not necessarily exhibit this quadratic dependence. Fig. S10B depicts examples of graphs whose outputs will instead display a response behavior mathematically evocative of detailed balance, a ratio of linear polynomials. We can see this contrasting behavior by recalling that a valid spanning tree cannot have more than one outgoing edge per node,

nor can it form a complete cycle, meaning that the illustrated graphs will give spanning trees with at most one edge dependent on the control parameter.

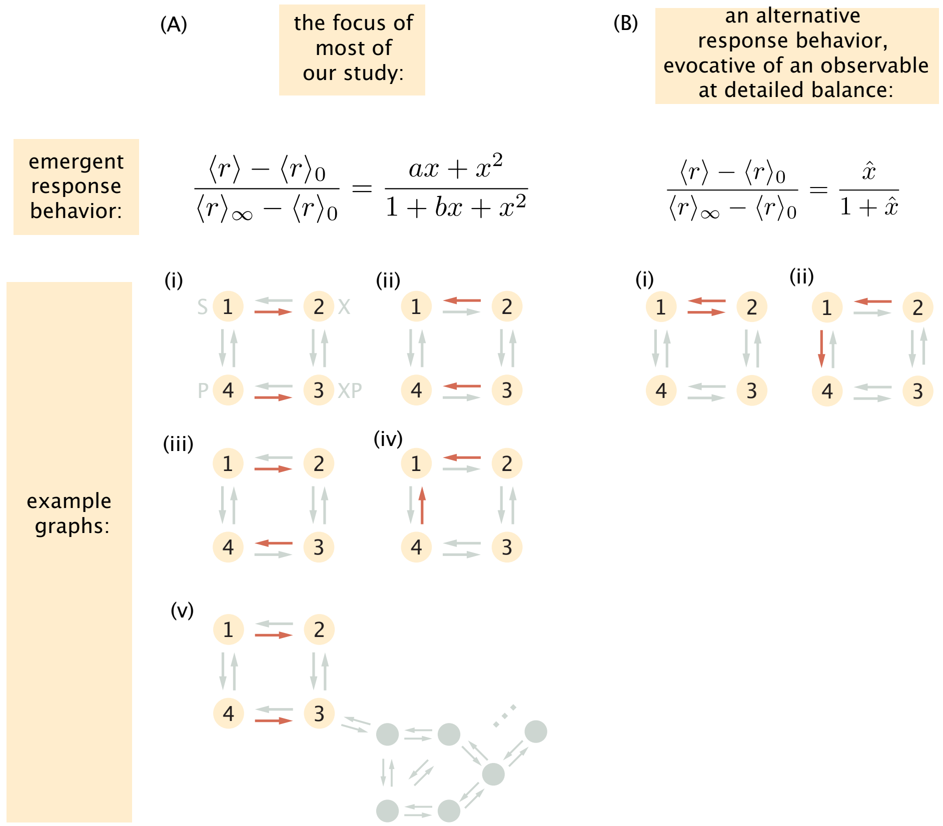

Fig. S10. Examples of alternative graph architectures that display (A) the same ratio-of-quadratic-polynomial dependence of the observable (and hence simplified two-parameter emergent shape behavior) in the control parameter, or (B) an observable behavior that evokes a detailed-balance response instead. The red arrows represent transitions whose rates are simultaneously scaled by the control parameter (such as a given transcription factor's concentration); these graphs differ largely by the location of these labeled edges in the underlying structure of the graph.

I. Any averaged observable $\langle r\rangle$ has zero, one, two, or three inflection points, with varying monotonicity.

I.1. Descartes' rule of signs on second-derivative-polynomial with (a, b) reveals precise restrictions on numbers of inflections. Descartes' rule of signs states that a polynomial $a_0 + a_1x + a_2x^2 + \cdots + a_nx^n$ with real coefficients $\{a_i\}$ has at most as many positive roots P as the number of changes in sign S in the sequence $a_0, a_1, \ldots, a_n$ (ignoring coefficients that are zero). Further, this count of the coefficients' sign changes S and the number of positive roots P differ by an even number (55).

Combined with the convenience of the reduced (a, b) shape parameterization, this rule gives transparent and straightforward information about how many inflection points the observable $\langle r\rangle$ may exhibit with respect to the (log) control variable. These inflection points satisfy $\frac{d^2\langle r\rangle}{d(\ln x)^2} = 0$. Since the (changes in) concavity are unchanged by scaling or shifting the function, we can evaluate this equation with respect to the normalized response in terms of the two (a, b) parameters—as in Eq. [31]—instead of the six parameters of the raw quadratic response. Computing the derivative gives

We first compute the first and second derivative of $\langle\tilde{r}\rangle$ with respect to x.

$$\frac{d\langle\tilde{r}\rangle}{dx} = \frac{(a+2x)(1+bx+x^2) - (ax+x^2)(b+2x)}{(1+bx+x^2)^2} = \frac{(b-a)x^2 + 2x + a}{(1+bx+x^2)^2} \quad [76]$$

$$\frac{d^2\langle\tilde{r}\rangle}{d^2x} = \frac{2(1+(b-a)x)(1+bx+x^2)^2 - 2((b-a)x^2+2x+a)(b+2x)(1+bx+x^2)}{(1+bx+x^2)^4} \quad [77]$$

$$= 2\frac{(1+(b-a)x)(1+bx+x^2) - ((b-a)x^2+2x+a)(b+2x)}{(1+bx+x^2)^3} \quad [78]$$

$$= 2\frac{x^3(a-b) - 3x^2 - 3ax - ab + 1}{(1+bx+x^2)^3} \quad [79]$$

Using these expressions, we compute the second derivative of the normalized rate with respect to x in a logarithmic scale.

$$\frac{d^2\tilde{\langle r\rangle}}{d^2 \ln x} = x\left(\frac{d\tilde{\langle r\rangle}}{dx} + x\frac{d^2\tilde{\langle r\rangle}}{d^2 x}\right) \quad [80]$$

$$= x\frac{((b-a)x^2+2x+a)(1+bx+x^2)+2x(x^3(a-b)-3x^2-3ax-ab+1)}{(1+bx+x^2)^3} \quad [81]$$

$$= x\frac{(a-b)x^4+\left(-ab+b^2-4\right)x^3+(3b-6a)x^2+(4-ab)x+a}{(1+bx+x^2)^3}, \quad [82]$$

where $\tilde{\langle r\rangle} \equiv \frac{\langle r\rangle - \langle r\rangle_0}{\langle r\rangle_\infty - \langle r\rangle_0}$.

This vanishes when the polynomial in the numerator vanishes; so we focus on

$$q(x) \equiv (a-b)x^4 + \left(-ab+b^2-4\right)x^3 + (3b-6a)x^2 + (4-ab)x + a. \quad [83]$$

Recalling that b is strictly positive, consider the possible changes in sign in this sequence of coefficients, rewritten suggestively as

$$\{a, 4-ab, 3(-(a-b)-a), -b(a-b)-4, a-b\}.$$

These coefficients' signs are constrained differently depending on when a is respectively positive, negative, or zero:

- $a < 0$**:** When all coefficients are nonzero, the signs are $\{\ominus, \oplus, \oplus, \ominus \text{ OR } \oplus, \ominus\}$. This means the sign sequence is either $\{\ominus, \oplus, \oplus, \ominus, \ominus\}$ (giving $S = 2$ sign changes) or $\{\ominus, \oplus, \oplus, \oplus, \ominus\}$ (still giving $S = 2$ sign changes). (While some of these coefficients can go to zero at certain (a, b), shortening the sign sequence, these happen to leave the number of sign changes unchanged from $S = 2$.) Hence when $a < 0$ there are exactly $\boxed{\text{zero or two}}$ (positive) inflection points: in other words, every nontrivial input-output curve with $a < 0$ has two inflection points.

- $a = 0$**:** Now the signs (of nonzero coefficients) are $\{\oplus, \oplus, \oplus \text{ OR } \ominus, \ominus\}$. Observe that there is exactly $S = 1$ sign change. (This is unchanged even if the third coefficient vanishes). So input-output curves with $a = 0$ must have exactly $\boxed{\text{one}}$ inflection point (they are "equilibrium-like").

- $a > 0$**:** Here the sign of $a - b$ critically affects how many positive roots exist:
  - If $a > b$, the signs are $\{\oplus, \oplus \text{ OR } \ominus, \ominus, \ominus, \oplus\}$; hence $S = 2$ sign changes permit exactly $\boxed{\text{zero or two}}$ positive inflection points.
  - If $a < b$, the signs are $\{\oplus, \ominus \text{ OR } \oplus, \ominus \text{ OR } \oplus, \ominus \text{ OR } \oplus, \ominus\}$. Hence there are up to $S = 3$ sign changes, permitting $\boxed{\text{one or three}}$ positive inflection points.

In general, this analysis has often benefited from the fact that if the signs of two or more coefficients are fixed at key positions in the coefficient sequence, then ambiguity in the signs of the coefficients in between has no effect on the number of possible changes of sign. For instance, the fact that the zeroth and fifth coefficients are respectively positive $\oplus$ and negative $\ominus$ in the last $0 < a < b$ case just examined immediately ensures that $S < 4$, so there are not four inflection points possible here (despite initial impressions from the fact that the underlying polynomial is a quartic).

The general conclusions we have reached from this elementary application of Descartes' rules are wholly consistent with a more precise, and algebraically-elaborate, inspection of the inflection points in the (a, b) space, as now follows. (We give both analyses because the former may add some transparency.)

I.2. Monotonicity of response via (a, b) ***parameterization.*** Here, we find the conditions on the emergent shape parameters (a, b) participating in the normalized response of Eq. [31] that assure nonmonotonicity. Since the logarithm is itself a monotonic transformation, the (non)monotonicity of responses remains unchanged whether we regard them with respect to the input variable on a linear scale or logarithmic scale. So for algebraic convenience, we inspect the first derivative of the response Eq. [31] with respect to the input on a linear scale, finding

$$\frac{d\langle r\rangle}{dx} = (\langle r\rangle_\infty - \langle r\rangle_0)\frac{(b-a)x^2+2x+a}{(x(b+x)+1)^2}. \quad [84]$$

The response $\langle r\rangle(x)$ is nonmonotonic if this derivative changes sign. Since x must be positive on physical grounds (as when it represents a concentration), we further demand that the derivative change sign for some $x > 0$. The polynomial in the derivative's numerator, $p(x) \equiv (b-a)x^2 + 2x + a$, behaves according to its discriminant

$$\Delta \equiv 4(1 - a(b-a)), \quad [85]$$

and the roots

$$x_\pm = \pm\sqrt{\frac{a^2-ab+1}{(a-b)^2}} + \frac{1}{a-b} = \frac{1}{a-b}\left(1 \pm \sqrt{1+a(a-b)}\right). \quad [86]$$

This polynomial has real solutions when the discriminant is nonnegative, $\Delta \geq 0$, namely, $1 - a(b-a) \geq 0$. Recalling that $b > 0$ by construction, one way for this to happen is when $a < 0$. Another way for the discriminant to be positive is when $a > 0$ while still ensuring that $a(b-a) < 1$, or equivalently $0 < b < a + \frac{1}{a}$.

The requirement that at least one root be positive further refines these conditions on (a, b). We proceed by inspecting the positivity of roots under each possible condition that ensures they are real

- $a < 0$**:** Only the root $x_- = \frac{1}{a-b} - \frac{1}{a-b}\sqrt{1 + a(a-b)}$ could be positive, since sign $\left(\frac{1}{a-b}\right) = \ominus$. In this case, we still need to verify that this root $x_- > 0$; this is true when $1 - \sqrt{1 + a(a-b)} < 0$. Happily this must be true, since $a(a-b)$ is a positive number, meaning the term in the square root is greater than one and so the square root is also greater than one. Hence, the case of $a < 0$ automatically ensures there is a real and positive solution to the inflection point changing sign (and thus nonmonotonicity).

- $0 < b < a + \frac{1}{a}$**, but** $b > a > 0$: Since a is now positive but still smaller than b, we still have sign $\left(\frac{1}{a-b}\right) = \ominus$, still suggesting x_+ cannot be positive. However, in this case, we further see that $1 + a(a-b) < 1$, so the other root x_- is also negative. Therefore, this condition does not guarantee nonmonotonicity.

- $a > b > 0$: Now, sign $\left(\frac{1}{a-b}\right) = \oplus$, and the term under the square root in the discriminant is greater than one. This means that only the root x_+ can be positive, which is automatically the case. Hence $a > b$ suffices to ensure nonmonotonicity.

(We also note that the discriminant cannot vanish and also produce a positive $x > 0$, ensuring these are the only conditions enabling nonmonotonicity.) Altogether, we summarize the necessary and sufficient conditions for nonmonotonicity, where a, b are defined, as

$$\boxed{\text{nonmonotonicity} \equiv \begin{cases} a > 0 \text{ and } a > b, \text{or} \\ a < 0 \text{ and } b > 0 \end{cases}}. \qquad [87]$$

When we return shortly to consider the number of inflection points possible for a response curve, we will see that these conditions for nonmonotonicity only intersect the conditions for having two inflection points, establishing that singly or triply inflected responses must be monotonic.

I.3. Bounds on the absolute magnitudes of response extrema. If a response is monotonic, then for any $[X]$, it must always be bounded above and below by the leakiness and saturation values $\langle r\rangle_0$ or $\langle r\rangle_\infty$. So finding an upper or lower bound on the response only becomes more subtle and interesting in the case of nonmonotonic responses.

To make progress, we translate the nonmonotonicity conditions Eq. [87] more concretely in term of the values $\frac{B}{E}$, $\langle r\rangle_0$ and $\langle r\rangle_\infty$. This process shows that a response is nonmonotonic if any of the following conditions are true:

$$\begin{cases} \text{condition 1:} & \langle r\rangle_\infty > \langle r\rangle_0 > \frac{B}{E}, \text{ or} \\ \text{condition 2:} & \frac{B}{E} > \langle r\rangle_\infty > \langle r\rangle_0, \text{ or} \\ \text{condition 3:} & \frac{B}{E} > \langle r\rangle_0 > \langle r\rangle_\infty, \text{ or} \\ \text{condition 4:} & \langle r\rangle_0 > \langle r\rangle_\infty > \frac{B}{E}. \end{cases} \qquad [88]$$

In general, this reasoning establishes that for any type of response (nonmonotonic or monotonic),

$$\min\left\{\langle r\rangle_0, \langle r\rangle_\infty, \frac{B}{E}\right\} \leq \langle r\rangle \leq \max\left\{\langle r\rangle_0, \langle r\rangle_\infty, \frac{B}{E}\right\}. \qquad [89]$$

Returning to the individual conditions for nonomonotonicity, we see they each give separate bounds for the extremal values of the observable:

$$\begin{cases} \text{condition 1:} & \frac{B}{E} \leq \langle r\rangle \leq \langle r\rangle_\infty \\ \text{condition 2:} & \langle r\rangle_0 \leq \langle r\rangle \leq \frac{B}{E} \\ \text{condition 3:} & \langle r\rangle_\infty \leq \langle r\rangle \leq \frac{B}{E} \\ \text{condition 4:} & \frac{B}{E} \leq \langle r\rangle \leq \langle r\rangle_0. \end{cases} \qquad [90]$$

Therefore the quantity $\frac{B}{E}$ bounds the extremum of any nonmonotonic response function.

The upper and lower bounds on any observable, Eq. [89], follow from a simple elementary result bounding ratios of sums. We quickly digress to establish the elementary result:

Simple bound on ratios of non-negative sums. For nonnegative a_i, b_i,

$$\min_i \left(\frac{a_i}{b_i}\right) \leq \frac{\sum_{i=1}^{N} a_i}{\sum_{i=1}^{N} b_i} \leq \max_i \left(\frac{a_i}{b_i}\right). \quad [91]$$

Consider the lower bound/left inequality. By definition, we know

$$\min_i \left(\frac{a_i}{b_i}\right) \leq \frac{a_j}{b_j}, \text{ for all } j \in [1, N] \quad [92]$$

Multiplying by b_j on both sides,

$$\min_i \left(\frac{a_i}{b_i}\right) b_j \leq a_j, \text{ for all } j \in [1, N] \quad [93]$$

and summing over all j gives

$$\min_i \left(\frac{a_i}{b_i}\right) \times \sum_{j=1}^{N} b_j \leq \sum_{j=1}^{N} a_j. \quad [94]$$

Hence indeed, $\min_i \left(\frac{a_i}{b_i}\right) \leq \frac{\sum_{j=1}^{N} a_j}{\sum_{j=1}^{N} b_j}$ as desired. The right (upper bound) inequality follows identically.

Returning to the ratio of polynomials form $\langle r \rangle = \frac{A+B[X]+C[X]^2}{D+E[X]+F[X]^2}$, this means that

$$\min\left\{\frac{A}{D} = \langle r \rangle_0, \frac{B}{E}, \frac{C}{F} = \langle r \rangle_\infty\right\} \leq \langle r \rangle \leq \max\left\{\frac{A}{D} = \langle r \rangle_0, \frac{B}{E}, \frac{C}{F} = \langle r \rangle_\infty\right\}, \quad [95]$$

which supports the claim of Eq. [89] and Eq. [90].

I.4. Number of inflection points via the (a, b) parameterization. Now we study the number of inflection points of the observable with respect to the control parameter on a logarithmic scale. To do this, we study the polynomial that appears in the numerator of the second derivative with respect to log control variable, Eq. [82],

$$q(x) \equiv x^4(a-b) + x^3\left(-ab + b^2 - 4\right) + x^2(3b - 6a) + x(4 - ab) + a. \quad [96]$$

In what follows, we examine how many roots of this polynomial can simultaneously be real and positive. As a preview of this logic, we do this by solving for each of the roots of the quartic; finding independent conditions on the parameters a, b that ensures each of these roots would be positive and real; then consider all the possible logical unions of these conditions, testing whether zero up to four inflections are simultaneously defined. We largely perform this tedious procedure using the symbolic capabilities of *Mathematica*—see our Github code repository for more details—and do not suggest that the intermediate conditions on individual roots are themselves enlightening or transparent. Yet their collective implications are meaningful and so we summarize them below.

The polynomial Eq. [96] can have up to four roots; denote them (x_1, x_2, x_3, x_4). These roots have a closed-form solution given by the famously grotesque quartic formula or returnable by *Mathematica*. Asking each of them to be positive and real gives individual conditions on (a, b); denote these conditions C_1, C_2, C_3, C_4, where C_i is the set of conditions where root x_i is real and positive. Then the condition of finding zero inflection points is the setting where none of C_1, C_2, C_3, or C_4 are true; the condition of finding one inflection point is where exactly one of them is true; and so on.

This analysis reveals two trivial cases. First, when there are no inflection points, the response transpires to be constant everywhere for all positive x, namely $\langle r \rangle = \langle r \rangle_0 = \langle r \rangle_\infty$. Second, we find that since not all of C_1, C_2, C_3, C_4 can be simultaneously true, it is impossible for the function to have four inflection points.

In contrast, it is readily possible to reach one, two, or three inflection points under specific parametric conditions. The borders between these conditions have somewhat complicated structure, particularly between the one and three inflection point cases. To assist us in expressing them as concisely as feasible, define the polynomial

$$H_a(b) \equiv -1024 - 1024a^2 + 1024ab + (-64 - 64a^2)b^2 + 64ab^3 + (-28 - a^2)b^4 + ab^5, \quad [97]$$

and in particular define its three real and positive roots when solving it with respect to the shape parameter b given a: denote them $b_1(a), b_2(a), b_3(a)$. (These roots turn out to form independent branches of an implicit representation of the border between

one and three inflection point regimes, each valid for different restricted values of a.) The final ingredient needed to define the borders between logical conditions turns out to be a numerical constant cutoff value of a, approximately $a_{\rm lim} \approx 2.35$ (see Mathematica code on Github and figure S11). Armed with these ingredients, the conditions to reach one, two, and three inflection point curves are expressed as follows, and plotted explicitly in Figure S11.

Output curves are "equilibrium-like," presenting only one inflection point, when

$$\boxed{\textbf{one inflection, monotonic} \equiv (b \le b_1(a) \text{ or } (b_3(a) \ge b \ge b_2(a), a \in [2, a_{\rm lim}])) \text{ and } a \ge b \text{ or } a = 0}. \qquad [98]$$

It transpires that output curves have two inflection points exactly under the same conditions on a, b as we found assured nonmonotonicity in Eq. [87]: namely,

$$\boxed{\textbf{two inflections, nonmonotonic} \equiv \begin{cases} a > 0 \text{ and } a > b, \text{or} \\ a < 0 \text{ and } b > 0 \end{cases}}. \qquad [99]$$

(Note that this condition also subsumes the case $a = \pm\infty$, where the observable is also nonmonotonic.)

Output curves show three inflection points if,

$$\boxed{\textbf{three inflections, monotonic} \equiv b > b_1(a) \text{ and } (b > b_3(a) \text{ or } b_2(a) > b, a \in [2, a_{lim}])}. \qquad [100]$$

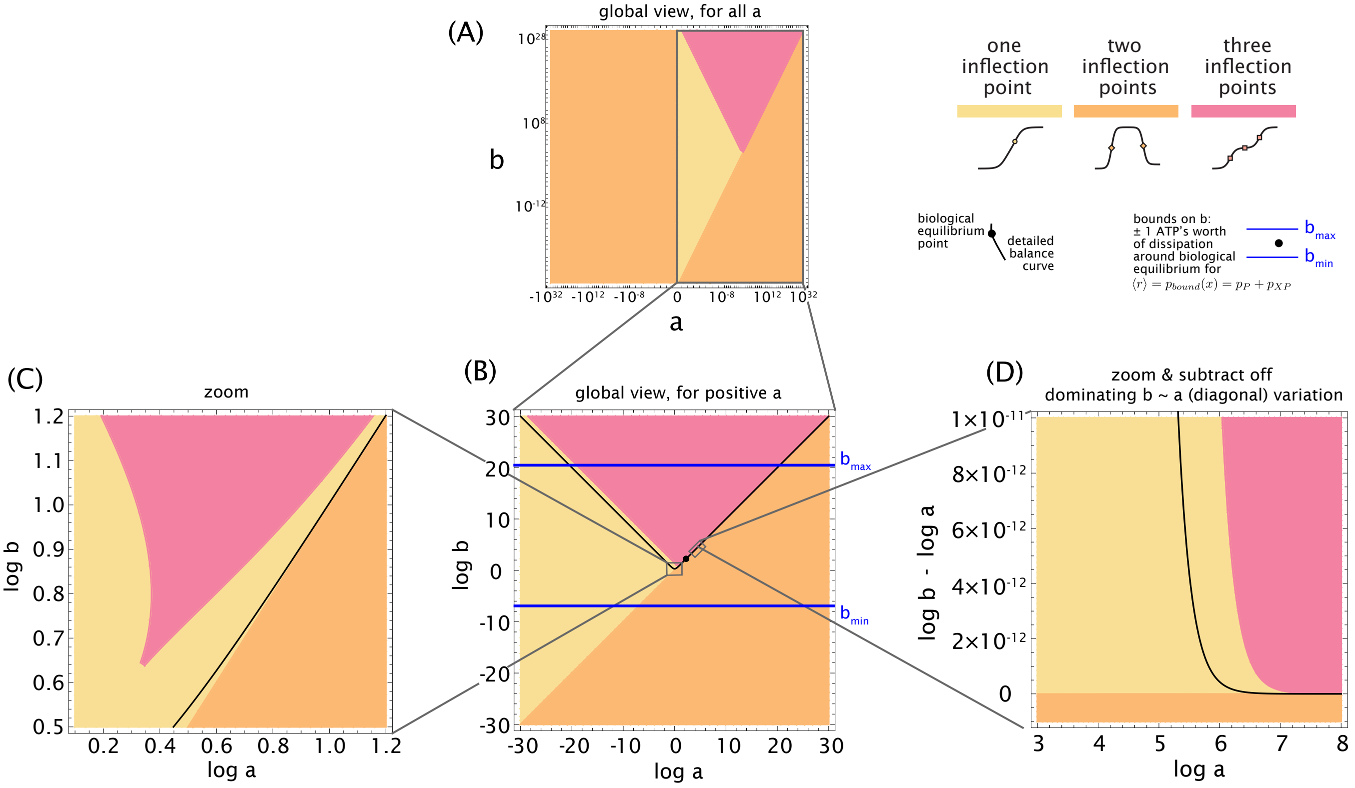

Fig. S11. The values of natural parameters (a, b) completely determine the shape of each response curve. Quantitative criteria partition the space into regions with either one inflection (pale yellow), two inflections (orange), or three inflections (pink). The central panels (A) and (B) give global views of (a, b) phase space centered around biological equilibrium, either for both positive and negative a (panel A) or for the subset $a > 0$ (panel B). Blue lines indicate the minimum and maximum values of b reachable by driving any single edge at a time by $\Delta\mu \le 20 k_B T$. When $a < 0$, response curves are always nonmonotonic (with two inflection points). Overall, the two-inflection-point phenotype is the most common in this space (for all a; the subspace where a is positive; or in the region where $a > 0; b \in [b_{min}, b_{max}]$). Systems satisfy detailed balance on the black line. The black dot denotes the default equilibrium starting rates reported in Fig. 1A of the main text, or Fig. S16. At left in (C) is a zoom of the same space near biological equilibrium, validating that the detailed balance curve always lies within the one-inflection thinly-shaped region that bridges the two-inflection point and three-inflection point regions. At right is another zoom of the ribbon region, but where the major diagonal covariation of b with a has been subtracted away (by plotting $\log b - \log a$ versus a instead of $\log b$ versus $\log a$). This visualizes how the detailed balance curve becomes asymptoptically closer to the border with the two-inflection-point regime (lower boundary/orange) versus the (upper boundary/pink) three-inflection-point regime as a grows larger.

We can summarize the border between one and three inflection point responses by considering the shape of this overall implicit function, $b_{\rm cutoff}(a)$, defined as

$$b_{\rm cutoff}(a) = \begin{cases} \max(b_1(a), b_2(a), b_3(a)) & \text{if } 2 \le a < a_{lim} \\ b_1(a) & \text{else} \end{cases} \qquad [101]$$

We visualize this cutoff function in Fig. S12.

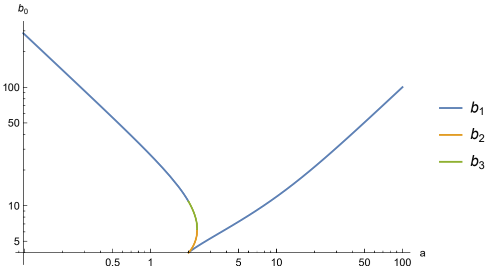

Fig. S12. The value of the cutoff $b_{\text{cutoff}}(a)$, defined in Eq. [101] with respect to a, delimits the first and third inflection points regimes.

At equilibrium, the collapse of an observable to a ratio of linear polynomials (Eq. [29]) allows us to rewrite the normalized response as

$$\langle r\rangle_{eq} = \langle r\rangle_0 + (\langle r\rangle_\infty - \langle r\rangle_0)\frac{dx}{1+dx}.$$

The constant d is the same in the numerator and denominator, so that the limit at infinity of the observable is $\langle r\rangle_\infty$. For the detailed balance case, we can identify $\langle r\rangle(x) = \langle r\rangle_{eq}(x)\forall x \in \mathbb{R}^{*+}$. This is equivalent to seeing the polynomial $R(X) = X(d(b-a)-1)+d-a$ have each of its coefficients vanish. This situation implies that the coefficients are related to one another according to,

$$\begin{cases} d = a \\ b = a + \frac{1}{a}. \end{cases} \qquad [102]$$

Note that the detailed balance curve always lies within the one-inflection (pale yellow) region: this region forms is a thin ribbon between the three and two inflection points region along the diagonal $a = b$. The detailed balance curve becomes asymptoptically closer to the border with the two-inflection-point regime (lower boundary/orange) versus the (upper boundary/pink) three-inflection-point regime as a grows larger (see Figure S11).

J. New bounds on nonequilibrium sensitivity.

J.1. Motivation of the the definition of the normalized sensitivity. Sensitivity—how steeply output changes with input—is one of the most fundamental quantitative traits that energy expenditure can modulate in biological systems, as celebrated by a plethora of famous biological models (e.g. the Goldbeter-Koshland ultrasensitivity mechanism (13), *inter alia*). Nonetheless, network architecture imposes strong constraints on the maximal sensitivities systems can achieve (1), even under arbitrarily large drive. We investigate sensitivity (and bounds thereof) for our setting in this spirit, but strive to use mathematical quantities that align closely with experimental conventions.

One common measure of sensitivity in conversation with experimental measurements and existing performance bounds is simply the (raw) *sharpness* (with respect to an input x),

$$\text{sharpness} \equiv \frac{d\langle r\rangle}{d\ln x} \qquad [103]$$

$$= x\frac{d\langle r\rangle}{dx}. \qquad [104]$$

Reference (28) is an example of a recent study which assesses sensitivity using this sharpness. The convention of considering changes in the raw response output with respect to a logarithmic input is also natural and coherent with the plotting convention of a logarithmic input, as discussed in §E. (If the response were exactly a Hill function with a Hill coefficient H, itself a common measure of sensitivity, then this sharpness would reach a maximal value of $H/4$ at the vertical midpoint of the response curve (1).) (When x is viewed as a concentration, we should recall that we render it unitless before taking the logarithm by viewing it as a normalized concentration relative to some reference $[X]_0$, say $[X]_0 \equiv$1 nanomolar, just as discussed in §E.)

To establish bounds on the sensitivity agnostic to specific parameter values or energetic dissipations, we normalize the raw sharpness, defining as our principal measure of *normalized sensitivity*,

$$\text{normalized sensitivity } s([X]) \equiv \left| \frac{d\langle r\rangle}{d\ln\left({[X]}/{[X]_0}\right)} \frac{1}{\langle r\rangle_{\max} - \langle r\rangle_{\min}} \right|. \qquad [105]$$

where we defined $\langle r\rangle_{\min} \equiv \min_{[X]} \langle r\rangle$ and $\langle r\rangle_{\max} \equiv \max_{[X]} \langle r\rangle$.

This definition of normalized sensitivity is related to the separately-normalized output $\tilde{r} \equiv \frac{\langle r\rangle - \langle r\rangle_0}{\langle r\rangle_\infty - \langle r\rangle_0}$ in ways that vary depending on the curve's shape. We review these relationships in each possible curve shape now. When the response remains monotonic (namely when it has one or three inflection points), the normalized sensitivity is equal to

$$\text{monotonic:} \quad s([X]) = \frac{d\langle r\rangle}{d\ln[X]}\frac{1}{\langle r\rangle_\infty - \langle r\rangle_0} = \frac{d\tilde{r}}{d\ln x}, \qquad [106]$$

since $\langle r\rangle_\infty - \langle r\rangle_0$ is the range of variation of the output curve.

When the output is nonmonotonic, if $a < 0$, then the output is first decreasing up to $\langle r\rangle_*$ and then increasing, since a is the value of the slope at zero concentration of the normalized rate. In this regime the maximum of slope is reached at second inflection. Hence, the corresponding range of variation of the rate is $\langle r\rangle_\infty - \langle r\rangle_*$, and the normalized sensitivity assumes the meaning

$$\text{nonmonotonic, } a<0\text{: } s([x]) = \frac{d\langle r\rangle}{d\ln[X]}\frac{1}{\langle r\rangle_\infty - \langle r\rangle_*} = \frac{d\tilde{r}}{d\ln x}\frac{\langle r\rangle_\infty - \langle r\rangle_0}{\langle r\rangle_\infty - \langle r\rangle_*} = \frac{d\tilde{r}}{dx}\frac{1}{1-\tilde{r_*}} = \frac{d\tilde{r}}{dx}\frac{1}{\tilde{r_\infty}-\tilde{r_*}} \qquad [107]$$

When the output is nonmonotonic but $\frac{a}{b} < 1$, the response is first increasing up to $\langle r\rangle_*$ and then decreasing to the value $\langle r\rangle_\infty$. The maximum of slope is reached at first inflection and the range of variation of the output values is $\langle r\rangle_* - \langle r\rangle_0$. Therefore the normalized slope becomes:

$$\text{nonmonotonic, } a/b<1\text{: } s([x]) = \frac{d\langle r\rangle}{d\ln[X]}\frac{1}{\langle r\rangle_* - \langle r\rangle_0} = \frac{d\tilde{r}}{d\ln x}\frac{\langle r\rangle_\infty - \langle r\rangle_0}{\langle r\rangle_* - \langle r\rangle_0} = \frac{d\tilde{r}}{d\ln x}\frac{1}{\tilde{r_*}} = \frac{d\tilde{r}}{d\ln x}\frac{1}{\tilde{r_*}-\tilde{r_0}}. \qquad [108]$$

J.2. Connection to other measures of sensitivity and the effective Hill coefficient. Here we clarify a few distinct but related notions of sensitivity. First, the *logarithmic sensitivity* of a response, measuring how inputs change a fold-change in response, is the response's logarithmic derivative with respect to its input,

$$\text{log. sensitivity} \equiv \frac{d\ln\langle r\rangle}{d\ln x} \qquad [109]$$

$$= \frac{1}{\langle r\rangle}\,\underbracket{\frac{d\langle r\rangle}{d\ln x}} \qquad [110]$$

$$= \frac{x}{\langle r\rangle}\frac{d\langle r\rangle}{dx}. \qquad [111]$$

The derivative of the raw response with respect to the log control variable, $\frac{d\langle r\rangle}{d\ln x}$ as emphasized with an underbracket in Eq. [111], is the raw sharpness we focus on throughout our analysis. It differs from logarithmic sensitivity only by a factor $\frac{1}{\langle r\rangle}$, whose own magnitude is bounded.

As discussed superbly and pedagogically by Owen and Horowitz (1), the logarithmic sensitivity is directly related to various notions of effective Hill coefficients. One definition of an effective Hill coefficient H_{eff} is explicitly proportional to the logarithmic sensitivity at a midpoint of the response (1), as used for example by references (56, 57):

$$H_{\text{eff}} \equiv 2\left.\frac{d\ln\langle r\rangle}{d\ln x}\right|_{x=x*} = 2\frac{1}{\langle r\rangle(x^*)}\left.\frac{d\langle r\rangle}{d\ln x}\right|_{x=x*} \qquad [112]$$

Hence the sharpness or normalized sensitivity we consider thus enjoys a close, though not identical, connection with these other measures of sensitivity such as effective Hill coefficients.

J.3. Summary of our results; contrast with existing bounds. As we report and illustrate in Figure 2 of the main text, we find that the normalized sensitivity is bounded by finite values,

$$\text{1 inflection:} \quad 0.158045 \le s\left([X]\right) \le \frac{1}{2}, \qquad [113]$$

$$\text{2 inflections:} \quad \frac{1}{4} \le s\left([X]\right) \le \frac{1}{2}, \qquad [114]$$

$$\text{3 inflections:} \quad \frac{1}{8} \le s\left([X]\right) \le \frac{1}{4}. \qquad [115]$$

Our main foundation for bounding response sensitivity is a dense numerical sampling of response curves facilitated by our two-dimensional representation of all responses: see Fig. S13. Specifically, we compute the normalized sensitivity on a fine grid of (a, b) values, observing the bounds above; we also symbolically simplify analogous logical conditions using *Mathematica*, finding concordance with these numbers. For instance, the curious number 0.158045 as a lower-bound on singly-inflected responses is reported with six decimals of precision because this was verified by explicit symbolic simplifications in *Mathematica*.

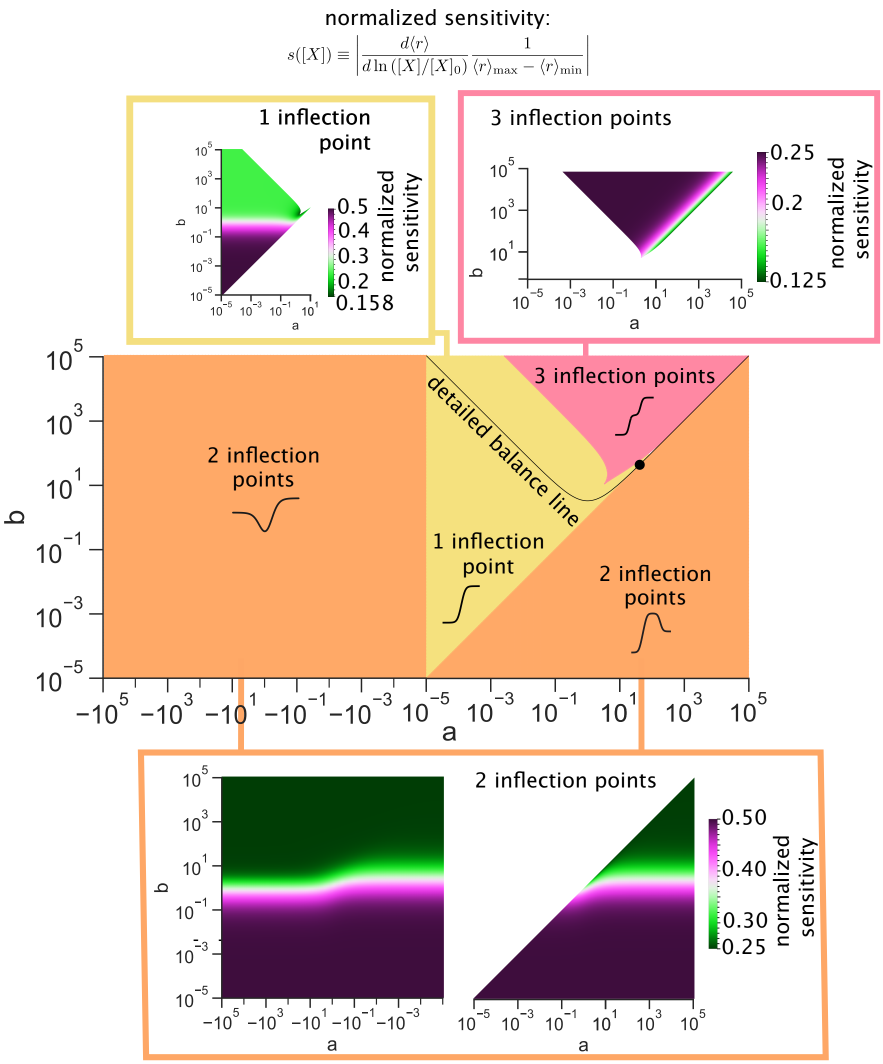

Fig. S13. Numerical validation of bounds on normalized maximal sensitivities over all curve phenotypes. Outset heatmaps depict the normalized sensitivities for curves of each region in (a, b) curve shape parameter space. Bounds are visible as the minimum and maximum sensitivities observed in each shape category.

To augment these numerical results, we provide some—albeit incomplete—analytical results; these follow in the next three subsections. First, we establish a looser global analytic upper bound on sensitivity, using a straightforward extension of recently-established upper bound arguments (1) on a related, differently-normalized slope. Second, we establish symbolically a slightly tighter global upper bound for monotonic outputs, that $s([X]) \leq \frac{1}{2}$. Last, for triply-inflected curves, we demonstrate symbolically both of our lower and upper bounds, $\frac{1}{8} \leq s([X]) \leq \frac{1}{4}$.

In conclusion, however, we continue to lack elegant or insightful analytical justifications for all of the lower bounds across regulatory shape phenotypes, or the upper bound on nonmonotonic responses, that we discover in numeric sampling. Interpretably demonstrating these behaviors will be a natural, fruitful subject of analytical work in the future.

J.4. General upper bound on a related, differently-normalized slope. Here we prove a (weaker) upper bound on a different sensitivity, closely connected with the fertile results of Owen and Horowitz (1). We will show that

$$\left|\frac{d\langle r\rangle}{d\ln x}\frac{1}{r_{\max}-r_{\min}}\right| \leq \frac{1}{2}, \qquad [116]$$

where we define the (unbracketed) quantities $r_{\min} \equiv \min_{\text{states } i} r_i$ and $r_{\max} \equiv \max_{\text{states } i} r_i$. We will call these quantities "theoretical" extrema because they are the ultimate extrema of observable weights over all microscopic states. Importantly these theoretical extrema are **not** the same as the (bracketed) quantities $\langle r\rangle_{\min} \equiv \min_{[X]} \langle r\rangle$ and $\langle r\rangle_{\max} \equiv \max_{[X]} \langle r\rangle$, the "observed extrema," that our actual normalized sensitivity transacts in. (We will return to contrast the implications of these extrema shortly, after we have established this weaker result.)

To proceed, we invoke a useful result from Owen and Horowitz (1), who establish that

$$\left|\frac{d\ln\langle O_1\rangle/\langle O_2\rangle}{d\ln x}\right| \leq m, \quad [117]$$

where $\langle O_1\rangle \equiv \sum_{\text{states } i} O_{1i}p_i$ and $\langle O_2\rangle \equiv \sum_{\text{states } i} O_{2i}p_i$ are observables defined by (positive) coefficients O_{1i}, O_{2i}; and m is the "size of the support," namely the number of states possessing at least one outgoing transition that is scaled by the control variable. Here in our square graph, $m = 2$.

Next, to invoke the normalization by extrema we desire, we choose the observable weights $O_{1i} \equiv r_i - r_{\min}$ and $O_{2i} \equiv r_{\max} - r_i$. These weights are clearly nonnegative, and so Eq. [117] applies. As a consequence, observe that $\langle O_1\rangle = \sum_i (r_i - r_{\min})p_i = \sum_i r_i p_i - r_{\min}\sum_i p_i = \langle r\rangle - r_{\min}$, and similarly $\langle O_2\rangle = r_{\max} - \langle r\rangle$. The bound Eq. 117 then becomes,

$$\frac{d\ln(\langle r\rangle - r_{\min})}{d\ln x} - \frac{d\ln(r_{\max} - \langle r\rangle)}{d\ln x} \leq m \quad [118]$$

$$\to \frac{1}{\langle r\rangle - r_{\min}}\frac{d\langle r\rangle}{d\ln x} - \frac{1}{r_{\max} - \langle r\rangle}\frac{-d\langle r\rangle}{d\ln x} \leq m \quad [119]$$

$$\to \frac{d\langle r\rangle}{d\ln x}\left(\frac{1}{\langle r\rangle - r_{\min}} + \frac{1}{r_{\max} - \langle r\rangle}\right) \leq m \quad [120]$$

$$\to \frac{d\langle r\rangle}{d\ln x}(r_{\max} - r_{\min}) \leq m(\langle r\rangle - r_{\min})(r_{\max} - \langle r\rangle). \quad [121]$$

On the right side, note that $\langle r\rangle - r_{\min}$ can be at most halfway between the minimum and maximum values of r, namely $(\langle r\rangle - r_{\min}) \leq \frac{r_{\max} - r_{\min}}{2}$. The same is true for $r_{\max} - \langle r\rangle$, e.g. $(r_{\max} - \langle r\rangle) \leq \frac{r_{\max} - r_{\min}}{2}$. So their product in the right-hand side is at most $\frac{(r_{\max} - r_{\min})^2}{4}$. This gives

$$\to \frac{d\langle r\rangle}{d\ln x}(r_{\max} - r_{\min}) \leq m\frac{(r_{\max} - r_{\min})^2}{4}, \quad [122]$$

or

$$\boxed{\frac{d\langle r\rangle}{d\ln x} \leq \frac{m}{4}(r_{\max} - r_{\min})}. \quad [123]$$

Substituting $m = 2$, as appropriate for the square graph, yields the desired result Eq. [116]. □

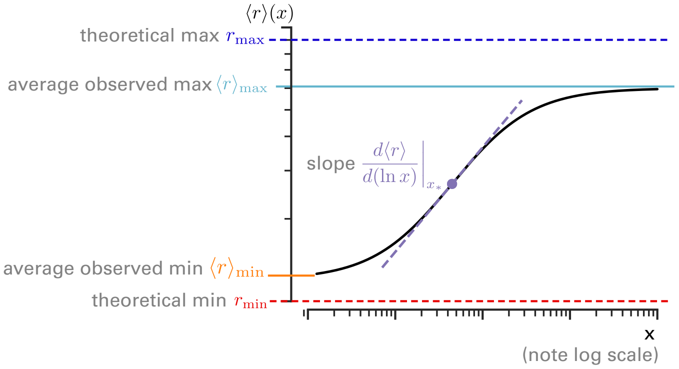

Fig. S14. Comparison of response extrema entering different bounds. In general, the observed minima of responses give tighter bounds on a particular response curve than theoretical minima of responses over microstates.

Now we contrast this result Eq. [116], defined in terms of the theoretical extrema $r_{\min}, r_{\max}$ over microstates, with our observed bounds on sensitivity defined in terms of the average *observed* extrema, $\langle r\rangle_{\min}, \langle r\rangle_{\max}$. In general, the theoretical response extrema themselves more conservatively bound the response than the observed response extrema. That is, in general the extrema of the *average* observable response curve over all $[X]$ are usually more restricted than the most extreme potencies over microstates (namely, $r_{\max} \equiv \max_i\{r_i\} \geq \langle r\rangle_{\max}$ and $r_{\min} \equiv \min_i\{r_i\} \leq \langle r\rangle_{\min}$. This property is visualized in Fig. S14.

Hence, for a *generic* response curve, the bounds Eq. [115] we discover and focus on in the main text of the paper are in fact tighter than that reported by Eq. 116.

One reason we study that normalized sensitivity $s([X]) \equiv \left|\frac{d\langle r\rangle}{d\ln x}\frac{1}{\langle r\rangle_{\max} - \langle r\rangle_{\min}}\right|$ is to try to connect more directly with measurements of biological curves that do not necessarily represent architectural optima. Indeed, for instance, the observable

weights (e.g. here, microscopic transcription rates) r_i of every microstate i are sometimes less easily known or convenient to measure (and so too their extremal values $r_{\max} \equiv \max_i\{r_i\}$ and $r_{\min} \equiv \min_i\{r_i\}$) than the average observable itself. Conversely, the observed extrema $\langle r\rangle_{\max}, \langle r\rangle_{\min}$ can often be directly "read off" from an averaged observable curve $\langle r\rangle([X])$.

We remark that when one is instead asking questions about optimal sensitivities realizable over all architectures, it is is plausible that these two styles of bound become equivalently informative. Specifically, as Jordon Horowitz suggests in personal communication, it is plausible that the response architectures which in fact saturate the bounds are also exactly those where $\langle r\rangle_{\min} \to r_{\min}$ and $\langle r\rangle_{\max} \to r_{\max}$.

J.5. General upper bound on our normalized sensitivity. Now, returning to our normalized slope $s([X]) = \left|\frac{d\langle r\rangle}{d\ln x}\frac{1}{\langle r\rangle_{max} - \langle r\rangle_{min}}\right|$ that is defined in terms of the observed (not theoretical) extrema, we show $s([X]) \leq \frac{1}{2}$ for all outputs.

For monotonic cases, we use the main result stated earlier from Reference (1), Eq. [117]. For simplicity, we note

$$\hat{r} = \frac{\langle r\rangle - \langle r\rangle_{min}}{\langle r\rangle_{max} - \langle r\rangle_{min}}, \quad [124]$$

where $\langle r\rangle_{min/max}$ is the minimum (maximum) value of the average observable $\langle r\rangle$ over all positive values of concentration $[X]$. Both $\langle O_1\rangle = \hat{r}$ and $\langle O_2\rangle = 1 - \hat{r}$ are rational functions with positive coefficients. Now, using the general expression of the output rate Eq. [27], we re-express the form of $\hat{r}$ as,

$$\hat{r} = \frac{(A - \langle r\rangle_{min}D) + (B - \langle r\rangle_{min}E)[X] + (C - \langle r\rangle_{min}F)[X]^2}{(D + E[X] + F[X]^2)(\langle r\rangle_{max} - \langle r\rangle_{min})}, \quad [125]$$

We note that D, E, F are by definition positive, because they are sums of positive weighted spanning trees. We recall that $\langle r\rangle_0 = \frac{A}{D}$, $\langle r\rangle_\infty = \frac{C}{F}$ so by definition of $\langle r\rangle_{min}$, $(A - \langle r\rangle_{min}D)$ and $(C - \langle r\rangle_{min}F)$ are positive coefficients. Furthermore, $(B - \langle r\rangle_{min}E)$ is positive for monotonic outputs, using the negation of non monotonicty condition Eq. [90]. Indeed the conditions for monotonicity can be expressed as,

$$\begin{cases} \text{condition 1:} & \langle r\rangle_\infty > \frac{B}{E} \text{ and } \langle r\rangle_0 < \frac{B}{E}, \text{ or} \\ \text{condition 2:} & \langle r\rangle_\infty < \frac{B}{E} \text{ and } \langle r\rangle_0 > \frac{B}{E}. \end{cases} \quad [126]$$

This conditions enforce the fact that $(B - \langle r\rangle_{min}E) > 0$, because since the function is monotonic $\langle r\rangle_{min} = \min(\langle r\rangle_\infty, \langle r\rangle_0)$.

Similarly, the observable $1 - \hat{r}$ is also a rational function with positive coefficients, with the following expression:

$$1 - \hat{r} = \frac{(\langle r\rangle_{max}D - A) + (\langle r\rangle_{max}E - B)[X] + (\langle r\rangle_{max}F - C)[X]^2}{(D + E[X] + F[X]^2)(\langle r\rangle_{max} - \langle r\rangle_{min})}. \quad [127]$$

With the same arguments as for the previous case, we show that all the coefficients of this rational function in $[X]$ are positive. Last, since $|s(x)| = \left|\frac{d\hat{r}}{d\ln x}\right|$, we recover $|s(x)| \leq \frac{1}{2}$ for monotonic outputs.□

Next, we consider nonmotonic responses. Here, we do *not* use the equality Eq. [117] because we can't define observables, which have the form of a positive rational function. Instead, we use the formalism of the coefficients a and b. Let us first settle to the case where $a > b > 0$. The extremum of the normalized function $\frac{\langle r\rangle - \langle r\rangle_0}{\langle r\rangle_\infty - \langle r\rangle_0}$ is then a maximum because $a = \frac{dr}{dx}|_{x=0}\frac{1}{\langle r\rangle_\infty - \langle r\rangle_0} > 0$, which implies that the output function first increases and then decreases and therefore reaches a maximum. The minimum of the normalized output is 0 because any increase or decrease of the concentration departing from the value that maximizes the output reduces the output value, by definition. So the minimum is reached at vanishing or infinite concentration. As these values for the normalized output are 0 or 1, we conclude that the minimum is 0. We call $\hat{r} = \frac{\langle r\rangle - \langle r\rangle_0}{\langle r\rangle_\infty - \langle r\rangle_0}$ and show that $\frac{d\hat{r}}{d\ln x} < \frac{1}{2\hat{r}_{max}}$, in order to prove that $s([X]) < \frac{1}{2}$. This is equivalent to showing that $\frac{\hat{r}_{max}}{2}x^4 + (a - b + b\hat{r}_{max})x^3 + (-2 + \hat{r}_{max} + \frac{b^2\hat{r}_{max}}{2})x^2 + (b\hat{r}_{max} - a)x + \frac{\hat{r}_{max}}{2} > 0$, with $\hat{r}_{max} = \frac{ab - 2(1+\sqrt{1+a^2-ab})}{-4+b^2}$. This is demonstrable by a direct appeal to Mathematica FullSimplify. The case $a < 0$ can be derived similarly.

J.6. Symbolic derivation of bounds for triply-inflected outputs. When the curve has three inflections, the normalized slope has 1/8 for its lower bound and 1/4 for its upper bound. We now demonstrate this behavior analytically.

For the upper bound, we aim to show that $s([X]) < \frac{1}{4}$ for all concentration $[X]$. First we notice that sensitivity with respect to the raw concentration is the same as the sensivity with respect to a renormalized concentration, $s([X]) = s(x)$. This is clear since sensitivity s is a derivative with respect to a logarithmic variable. Substituting our normalized response function in terms of (a, b), the desired upper sensitivity bound is equivalent to the following condition:

$$f(x) = 1 + 2(b - 2a)x + (b^2 - 6)x^2 - 2(b - 2a)x^3 + x^4 > 0. \quad [128]$$

We note that $f(0) = 1 > 0$ and that $\lim_{x\to\infty} f(x) = +\infty$, so if the function f remains positive on positive values of x the condition Eq. [128] is satisfied. The algebraic conditions assuring three inflection points, as discussed in §I.4, implies $1 + a^2 > ab$, which implies that the function f has no roots.

Indeed, we can prove this quick lemma. Specialize to the case where $b < 2a$. In this case, we study the sign of the polynomial $x(2(b-2a)+(b^2-6)x-2(b-2a)x^2)$. This polynomial vanishes at $x=0$ and at $x_+ = \frac{b^2-6-\sqrt{(b^2-6)^2+16(b-2a)^2}}{4(b-2a)}$. Therefore, this polynomial takes negative values between 0 and x_+ and positive for $x > x_+$. The minimal value is taken at $x_{min} = \frac{6-b^2+\sqrt{36+48a^2-48ab+b^4}}{6(2a-b)}$ and lies between 0 and x_+. The value at x_{min} of the function $f(x_{min})$ is positive if $1+a^2 > ab$. So in this case $f(x) > 0$.

For the case where $b > 2a$, we study the sign of the polynomial $x^2(b^2-6-2(b-2a)x+x^2)$, which is strictly positive because the associated discriminant of $b^2-6-2(b-2a)x+x^2$ is $\Delta = 16(a^2+\frac{3}{2}-ab)$ is negative if $1+a^2 > ab$.

Now we focus on the lower bound. We note that the maximum of slope is reached either at the 2nd of the 4th inflection, that we called x_2 and x_4. For we need to prove that it is impossible to have $s(x_2) < \frac{1}{8}$ and $s(x_4) < \frac{1}{8}$ for the same couple (a,b), while satisfying the algebraic condition for three inflection points. Indeed, this condition cannot be satisfied. Therefore, we conclude that a lower bound for the maximum of slope of the output over the whole (a,b) space is $\frac{1}{8}$. This is demonstrable by a direct appeal to Mathematica FullSimplify.

K. Systematic census of effects of driving one or two edges.

K.1. Scaling a single rate constant at a time is identified with a proportional drive. The cycle condition relating the ratio of rate constants to the net nonequilibrium driving force $\Delta\mu$ affords us concise expressions for how modifying individual rate parameters induces a net drive. In the main text (or more extensively shortly here in §K), we investigate breaking detailed balance edge-by-edge (while keeping seven rate constants fixed at their default equilibrium values). Say that we are modifying a rate constant k_{ij} away from its default equilibrium value $k_{ij}^{\text{eq.}}$. The cycle condition Eq. [21] implies that

$$\Delta\mu/k_BT = \ln\gamma = \ln\left(\frac{\prod_{i=1}^{N} k_{i,i+1}}{\prod_{i=1}^{N} k_{i+1,i}}\right) \quad [129]$$

$$= \ln\frac{k_{ij}}{k_{ij}^{\text{eq.}}}, \quad [130]$$

since $\gamma = 1$ at equilibrium.

By similar logic, when we adjust two rate constants at once, if they are oriented in the same clockwise or counterclockwise direction in the cycle, then the product of their multiplicative adjustments sets γ. If the rates are instead oriented in opposite directions around the cycle, the ratio of their multiplicative adjustments sets γ. In both cases, this new value of γ, which is non unitary set $\Delta\mu$.

K.2. Quantitative comparisons of response curves with and without rate symmetries enforced. To demonstrate the kinetic conditions (summarized in Eq. 5 of the main text, and derived in the subsequent subsection §L) governing how feasibly nonmonotonic response curves can be accessed, we broke detailed balance edge-by-edge departing from two different sets of default starting rate constants. These sets of rate constants differ only slightly; specifically, these rates differ only in whether critical imbalances among key rates are initially satisfied or not. The systematic censuses of how response curves change under driving all individual edges are reported in Figure S15 and Figure S16, respectively. Starting from a set of rates where crucial rate imbalances are present (Figure S15) allows dramatically more facile access to nonmonotonic and triply-inflected response curves compared to rates satisfying kinetic symmetries (Figure S16), although the raw values of the respective sets of starting rates (visualized in Figure S15A and S16A) differ only mildly.

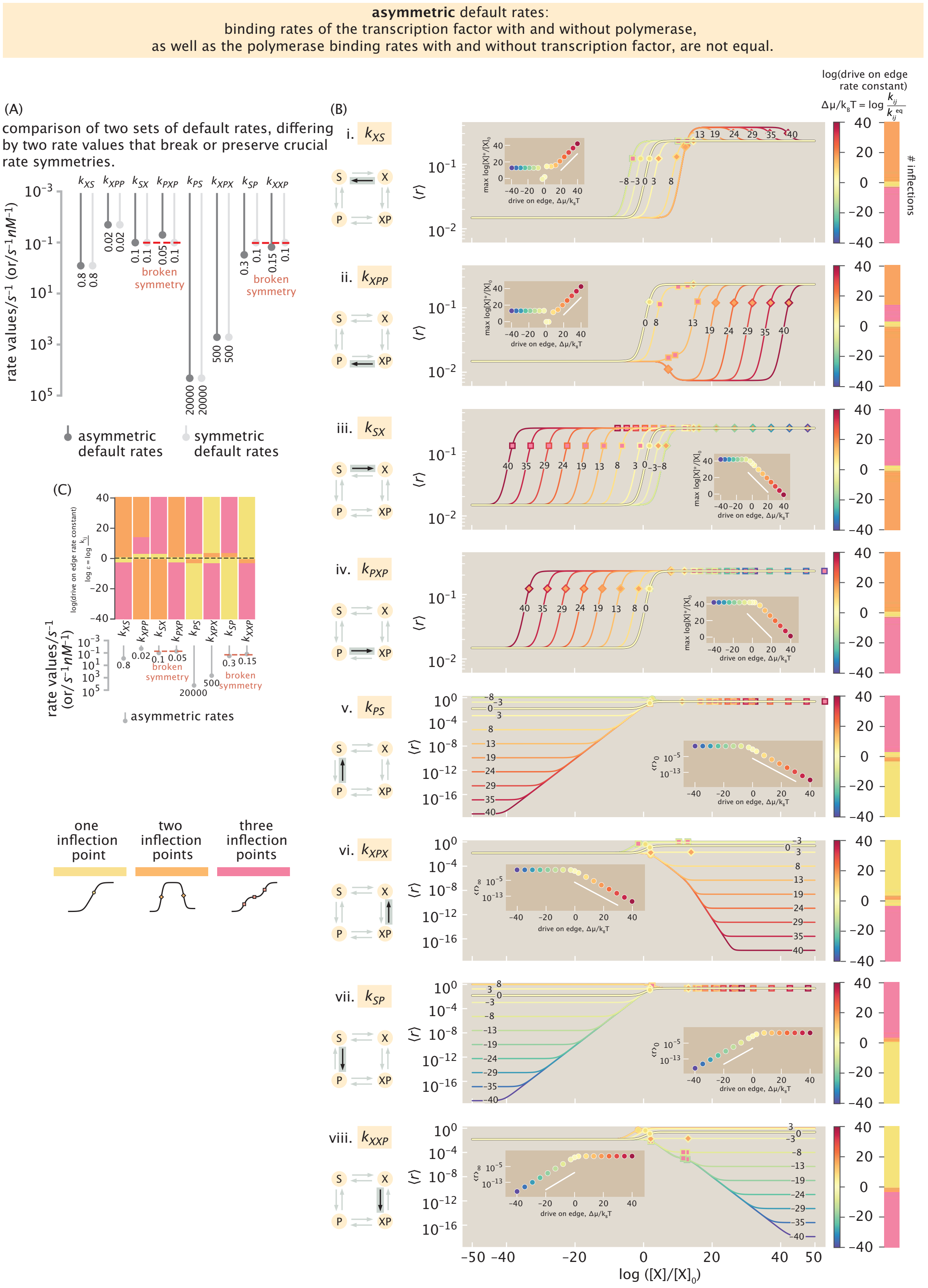

Fig. S15. Systematic census of breaking detailed balance, one edge at a time, departing from (slightly) asymmetric default values. These are the parameters used for Figures 3 and 4 of the main text; main text Figure 3 contains two panels of this set. Contrast, panel-by-panel, with the effects of pushing on the same rates, but at different starting values where some symmetries are preserved among the rates, shown in Fig. S16. In particular, notice that nonmonotonic responses (orange in phase space plots) are significantly less common than in Fig. S16. (A) Comparison of two sets of starting rates; the sets are the same for four rates, but vary by a factor of less than a few in the other rates, differing in whether critical symmetries are preserved or broken among the rates. (B) The effect of increasing or decreasing each individual rate on the input-output curve, while keeping seven other rates constant. Responses from rate values larger than (or smaller than) at equilibrium are shown in increasingly red (or blue) colors, respectively; curves are also labeled with the numerical values of the net drive that generated them in $k_B T$ units (positive for an increase; negative for a decrease). Each curve's resulting inflection points are marked by yellow, orange, or pink markers, denoting one to three inflection points (respectively), and summarized in the associated one-dimensional (shape phenotypic) phase-diagram with the same colors on the right. (C) Summary of how all eight rates respond to energy expenditure to realize different regulatory shape phenotypes.

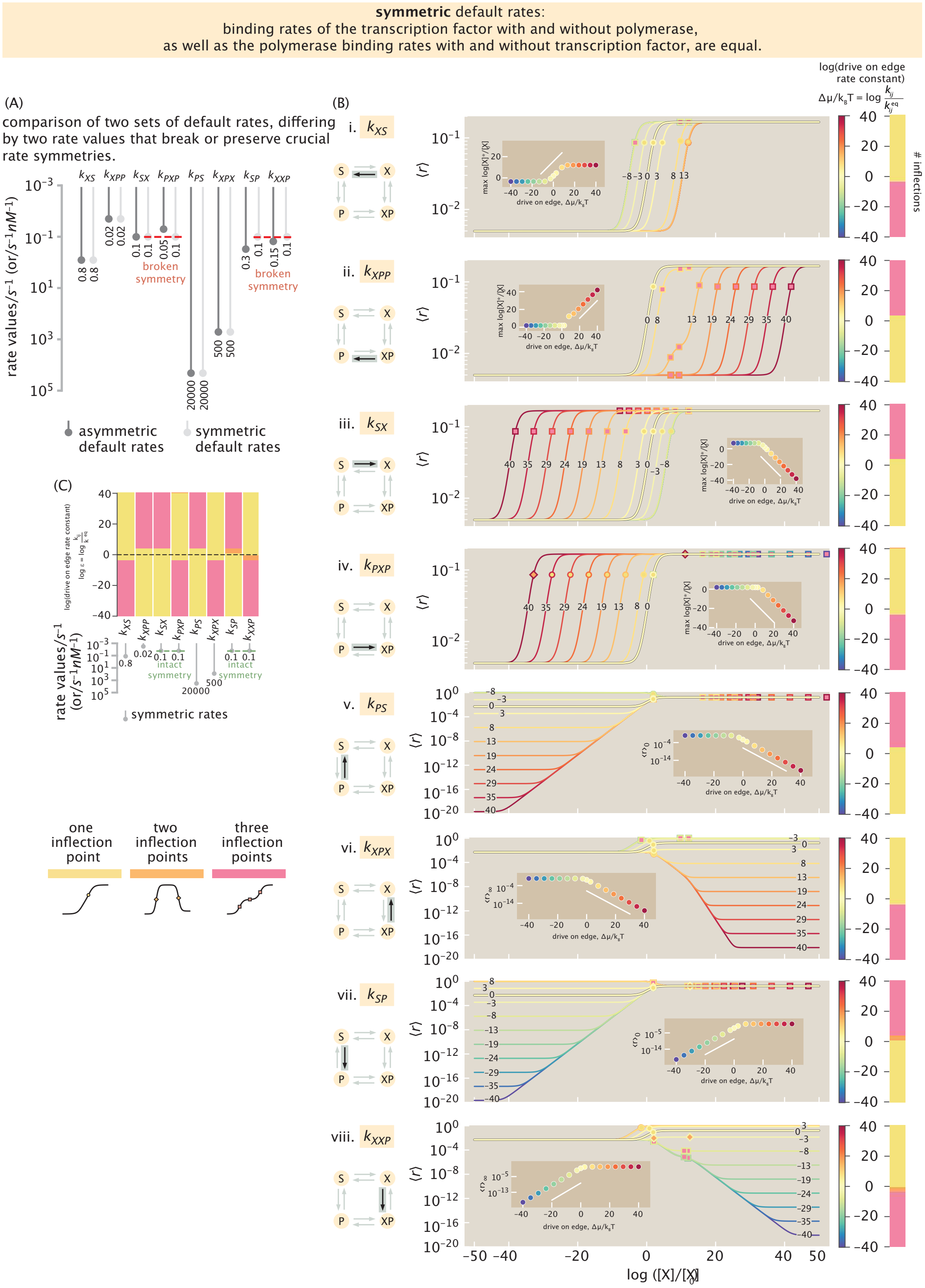

Fig. S16. Systematic census of breaking detailed balance, one edge at a time, departing from *symmetric* default values, meaning that the binding rates of the transcription factor with and without polymerase presence, as well as the polymerase binding rates with and without transcription factor, are equal. These are only slightly different than the default parameters used for Figures 3 and 4 of the main text, yet yield richly different behaviors in accessing nonmonotonicity and other phenotypes and illustrate different effects of control. Contrast, panel-by-panel, with Fig. S15. (A) Comparison of two sets of starting rates; the sets are the same for four rates, but vary by a factor of less than a few in the other rates, differing in whether critical symmetries are preserved or broken among the rates. (B) The effect of increasing or decreasing each individual rate on the input-output curve, while keeping seven other rates constant. Responses from rate values larger than (or smaller than) at equilibrium are shown in increasingly red (or blue) colors, respectively; curves are also labeled with the numerical values of the net drive that generated them in $k_B T$ units (positive for an increase; negative for a decrease). Each curve's resulting inflection points are marked by yellow, orange, or pink markers, denoting one to three inflection points (respectively), and summarized in the associated one-dimensional (shape phenotypic) phase-diagram with the same colors on the right. (C) Summary of how all eight rates respond to energy expenditure to realize different regulatory shape phenotypes.

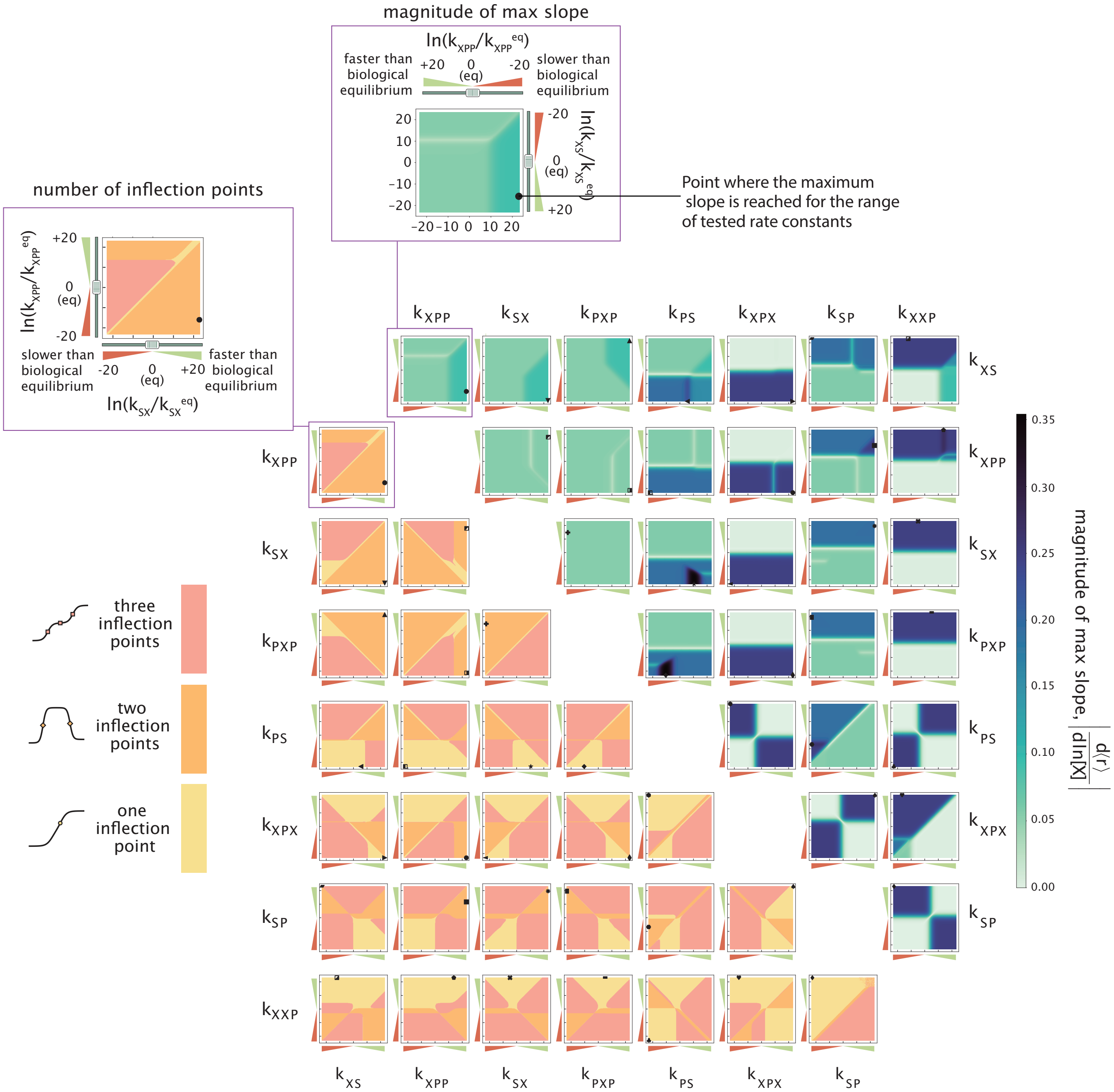

Fig. S17. Breaking detailed balance two-edges-at-a-time unlocks wide regions of rate-space where nonmonotonic and triply-inflected phenotypes are observed (lower left triangular matrix). The quantitative properties of the resulting output-curves, such as the slope at first inflection, are also modulated flexibly by these drives (upper right triangular matrix).

L. Crucial imbalances in rate-constants are required for nonmonotonic responses. In this section, we derive conditions on the values of rate constant that enable or forbid access to nonmonotonicity. In additional, we find the minimal (nonzero) net drive needed to access nonmonotonicity when kinetic conditions permit. By "kinetic conditions", we mean inequality on rate constants, which cannot be interpreted by Boltzmann ratios and therefore energies at equilibrium. We preview our strategy as follows. First, we translate each of the two conditions guaranteeing nonmonotonicity we found in Eq. [87] from the space of shape parameters (a, b) back into expressions purely in terms of the eight rate constants governing the system. Next, we compel γ—the product of rate constants in one direction around the cycle divided by the product taken in the opposite direction, whose logarithm gives the net drive, as discussed in §C and §D—to appear in these conditions, by substituting out one of the eight rate constants. We simplify the resulting expressions to surprisingly concise forms that yield minimal drives required to access nonmonotonicity. However, these critical drive values are only defined when precise imbalances among the rates are satisfied, thus establishing sufficient conditions to forbid nonmonotonicity.

1. **We start with the first way to reach nonmonotonicity according to Eq. [87], namely $0 < b < a$:**
For algebraic convenience, since this condition specifies the relative value of a and b, define $\alpha \equiv 1 - \frac{a}{b}$; this first nonmonotonicity condition is then expressed as $\alpha < 0$. Substituting the definitions of the shape parameters a, b (Eq. [38]) and the definitions of the coefficients A, B, C, D, E, F appropriate for the square graph (Eq. [27]) casts this condition back into the language of rate constants: nonmonotonicity is guaranteed when,

$$\alpha \equiv \frac{([P]k_{SP}+k_{PS})(k_{XXP}(-k_{XS}k_{XPX}k_{PXP}+k_{XPX}k_{XPP}k_{SX}-k_{XPP}k_{SX}k_{PS})+k_{XS}k_{XPX}k_{SP}k_{PXP})}{(k_{XPX}k_{SP}-k_{XXP}k_{PS})([P](k_{SP}k_{PXP}([P]k_{XXP}+k_{XS}+k_{XPX})+k_{XXP}k_{XPP}k_{SX})+k_{SX}k_{PS}([P]k_{XXP}+k_{XPX}+k_{XPP})+k_{XS}k_{XPX}k_{PXP})} < 0. \quad [131]$$

Next, we simplify by positive factors, and use fact that $\frac{1}{k_{XPX}k_{SP}-k_{XXP}k_{PS}}$ and $k_{XPX}k_{SP} - k_{XXP}k_{PS}$ have the same sign. Since we want to force $\gamma \equiv \frac{k_{XXP}k_{XPP}k_{PS}}{k_{SX}k_{XS}k_{XPX}k_{SP}k_{PXP}}$ to appear to comment on energetic drive, we choose a rate constant to express in terms of γ and the other seven rates. Without loss of generality, we choose to replace k_{SX} by $k_{SX} = \gamma \frac{k_{XS}k_{XPX}k_{SP}k_{PXP}}{k_{XXP}k_{XPP}k_{PS}}$. These manipulations convert Eq. 131 into the much more succinct and revealing form,

$$\left(\frac{k_{SP}}{k_{PS}} - \frac{k_{XXP}}{k_{XPX}}\right)\left(1 - \frac{k_{XXP}}{k_{SP}} - \gamma\left(1 - \frac{k_{XPX}}{k_{PS}}\right)\right) < 0. \quad [132]$$

Now, we solve for possible values of γ, under the mathematical constraints that γ must itself remain positive (that is, nonnegative because it is a ratio of positive rate constants, and greater than zero because we know nonmonotonic outputs cannot occur at detailed balance). We could solve this condition Eq. [132] by hand, case-by-case; but for ease we use a call to *Mathematica*'s `Reduce` command over γ on the `PositiveReals`, while enforcing assumptions that all rates are positive. This analysis generates all the specific possible conditions where γ is defined and satisfies this nonmonotonicity criterion; these transpire to be,

$$\begin{cases} 0 < \gamma < \frac{k_{PS}(k_{SP}-k_{XXP})}{k_{SP}(k_{PS}-k_{XPX})} \quad \text{and} \quad \begin{cases} k_{SP} < k_{XXP} & \text{and } k_{PS}k_{XXP} < k_{SP}k_{XPX} \text{ or,} \\ k_{SP} > k_{XXP} & \text{and } k_{PS}k_{XXP} < k_{SP}k_{XPX}. \end{cases} & \text{or ,} \\ \gamma > \frac{k_{PS}(k_{SP}-k_{XXP})}{k_{SP}(k_{PS}-k_{XPX})} \quad \text{and} \quad \begin{cases} k_{XPX} < k_{PS} < \frac{k_{SP}k_{XPX}}{k_{XXP}} \text{ or,} \\ \frac{k_{SP}k_{XPX}}{k_{XXP}} < k_{PS} < k_{XPX} \text{ and } k_{SP} < k_{XXP}. \end{cases} & . \end{cases} \quad [133]$$

Clearly this panoply of logical conditions is intricate. To interpret and summarize these conditions, we define some notation for the constituent kinetic conditions, which often have physical interpretations:

- First, recall that the conditions for the transcription factor to be an overall repressor or activator are simply given by,

$$\begin{cases} \text{activation, } A \equiv \frac{k_{SP}}{k_{PS}} < \frac{k_{XXP}}{k_{XPX}} \\ \text{repression, } R \equiv \frac{k_{SP}}{k_{PS}} > \frac{k_{XXP}}{k_{XPX}} \end{cases}. \quad [134]$$

- Next, for concision, denote the following pairwise conditions among rates as,

$$\begin{cases} c_1 \equiv \frac{k_{XPX}}{k_{PS}} > 1 \\ c_2 \equiv \frac{k_{XPX}}{k_{PS}} < 1 \\ c_3 \equiv \frac{k_{XXP}}{k_{SP}} > 1 \\ c_4 \equiv \frac{k_{XXP}}{k_{SP}} < 1 \end{cases}. \quad [135]$$

  (Note that c_1 and A imply c_3; c_2 and R imply c_4; c_4 and A imply c_2; and last, c_3 and R imply c_1.)

- Recalling that the net drive present in the cycle is given by $\Delta\mu = k_B T \ln \gamma$ (see §D), we now identify two constituent requirements for nonmonotonicity from those of Eq. [133], expressed in terms of $\Delta\mu$. We denote them c_+ and c_-, because satisfying them respectively reflects a clockwise stationary flux and a counterclockwise flux while allowing nonmonotonicity; denote their logical union the condition c. These are defined as,

$$c \equiv \begin{cases} c_+(k_{XXP}, k_{SP}, k_{XPX}, k_{PS}) \equiv (\Delta\mu > 0) \text{ and } ((c_1 \text{ and } A) \text{ or } (c_2 \text{ and } R)), \text{ or,} \\ c_-(k_{XXP}, k_{SP}, k_{XPX}, k_{PS}) \equiv (\Delta\mu < 0) \text{ and } ((c_4 \text{ and } A) \text{ or } (c_3 \text{ and } R)) \end{cases}. \quad [136]$$

Finally, we use all this notation to interpret Eq. [133] as saying that when rate constants satisfy the necessary conditions $c(k_{XXP}, k_{SP}, k_{XPX}, k_{PS})$ (Eq. [136]), a minimum critical drive $\Delta\mu_1$ exists, defined by

$$\boxed{\Delta\mu_1 = k_B T \left| \ln \frac{\frac{k_{XXP}}{k_{SP}} - 1}{\frac{k_{XPX}}{k_{PS}} - 1} \right|}. \qquad [137]$$

That is, when the drive $\Delta\mu$ exceeds this $\Delta\mu_1$ in magnitude under the right preexisting rate conditions,

$$|\Delta\mu| > \Delta\mu_1, \qquad [138]$$

responses are nonmonotonic.

2. **Next, we turn to the second way to reach nonmonotonicity according to Eq. [87], namely $a < 0$:** Analogously to how we treated the first nonmonotonicity condition, we translate $a < 0$ to $1 - \alpha < 0$ and substitute rate constants into the definitions, expressing the present nonmonotonicity condition as

$$\frac{([P]k_{XXP}+k_{XPX})(-k_{XPX}k_{SP}(k_{XS}+[P]k_{SP})k_{PXP}+(k_{XXP}k_{XPP}k_{SX}-(k_{XPX}+k_{XPP})k_{SX}k_{SP}+([P]k_{XXP}+k_{XS})k_{SP}k_{PXP})k_{PS}+k_{XXP}k_{SX}k_{PS}^2)}{(k_{XPX}k_{SP}-k_{XXP}k_{PS})(k_{XS}k_{XPX}k_{PXP}+[P](k_{XXP}k_{XPP}k_{SX}+([P]k_{XXP}+k_{XS}+k_{XPX})k_{SP}k_{PXP})+([P]k_{XXP}+k_{XPX}+k_{XPP})k_{SX}k_{PS})} < 0 \qquad [139]$$

Simplifying by the positive terms; noticing that $\frac{1}{k_{XPX}k_{SP}-k_{XXP}k_{PS}}$ and $k_{XPX}k_{SP} - k_{XXP}k_{PS}$ have the same sign; and replacing k_{SX} by $\gamma\frac{k_{XS}k_{XPX}k_{SP}k_{PXP}}{k_{XXP}k_{XPP}k_{PS}}$ recasts this condition as,

$$\left(\frac{k_{SP}}{k_{PS}} - \frac{k_{XXP}}{k_{XPX}}\right)\left(1 - \left(\frac{k_{PS}}{k_{XPX}} + \frac{[P]k_{XXP}k_{PS}}{k_{XS}k_{XPX}} - \frac{[P]k_{SP}}{k_{XS}}\right) - \gamma\left(1 - \left(\frac{k_{XPX}k_{SP}}{k_{XXP}k_{XPP}} + \frac{k_{SP}}{k_{XXP}} - \frac{k_{PS}}{k_{XPP}}\right)\right) < 0\ . \qquad [140]$$

Now, as before, we solve for the values of γ that are positive, real, and compatible with this condition. Since the resulting specific conditions are most intrepretable when expressed directly in terms of $\Delta\mu = k_B T \ln\gamma$, we report them directly in this variable. To do so, we again define some notation for governing subconditions that materialize as follows.

- Denote the following logical conditions with the shorthand d_i,

$$\begin{cases} d_1 \equiv (k_{XPX} + k_{XPP})k_{SP} < k_{XXP}(k_{XPP} + k_{PS}) \\ d_2 \equiv (k_{XPX} + k_{XPP})k_{SP} > k_{XXP}(k_{XPP} + k_{PS}) \\ d_3 \equiv ([P]k_{XXP} + k_{XS})k_{PS} < k_{XPX}(k_{XS} + [P]k_{SP}) \\ d_4 \equiv ([P]k_{XXP} + k_{XS})k_{PS} > k_{XPX}(k_{XS} + [P]k_{SP}) \end{cases}. \qquad [141]$$

- Then nonmonotonicity is possible, and rates induce clockwise (+) and counterclockwise (-) steady-state fluxes, respectively, when either of the conditions d_+ and d_- are satisfied,

$$d \equiv \begin{cases} d_+(k_{XXP}, k_{SP}, k_{XPX}, k_{PS}, k_{XPP}, k_{XS}) \equiv (\Delta\mu > 0 \text{ and } k_{XPP} > k_{XPX}k_{PS}\left|\frac{\frac{k_{SP}}{k_{PS}} - \frac{k_{XXP}}{k_{XPX}}}{k_{XXP} - k_{SP}}\right| \text{ and } ((c_3 \text{ and } R) \text{ or } (c_4 \text{ and } A)) \\ d_-(k_{XXP}, k_{SP}, k_{XPX}, k_{PS}, k_{XPP}, k_{XS}) \equiv (\Delta\mu < 0 \text{ and } k_{XS} > k_{XPX}k_{PS}\left|\frac{\frac{k_{SP}}{k_{PS}} - \frac{k_{XXP}}{k_{XPX}}}{[P](k_{XPX} - k_{PS})}\right| \text{ and } ((c_1 \text{ and } A) \text{ or } (c_2 \text{ and } R)), \end{cases} \qquad [142]$$

where we have denoted their logical union d.
We also remark that an alternative, equivalent way of expressing Eq. [142] is as follows,

$$d \equiv \begin{cases} d_+(k_{XXP}, k_{SP}, k_{XPX}, k_{PS}, k_{XPP}, k_{XS}) = (\Delta\mu > 0) \text{ and } ((d_1 \text{ and } c_3 \text{ and } R) \text{ or } (d_2 \text{ and } c_4 \text{ and } A)) \\ d_-(k_{XXP}, k_{SP}, k_{XPX}, k_{PS}, k_{XPP}, k_{XS}) = (\Delta\mu < 0) \text{ and } ((d_3 \text{ and } c_1 \text{ and } A) \text{ or } (d_4 \text{ and } c_2 \text{ and } R)). \end{cases} \qquad [143]$$

This notation allows us to interpret Eq. [140] as saying that when rates satisfy the conditions $d(k_{XXP}, k_{SP}, k_{XPX}, k_{PS}, k_{XPP}, k_{XS})$, there is a minimal drive $\Delta\mu_2$ past which nonmonotonicity is activated,

$$|\Delta\mu| > \Delta\mu_2, \qquad [144]$$

where

$$\boxed{\Delta\mu_2 = k_B T \left| \ln \frac{\frac{k_{XXP}}{k_{XPX}} - \frac{k_{SP}}{k_{PS}} + \frac{k_{XS}}{k_{XPX}P} - \frac{k_{XS}}{k_{PS}P}}{\frac{k_{XPX}}{k_{XXP}} + \frac{k_{XPP}}{k_{XXP}} - \frac{k_{XPP}}{k_{SP}} - \frac{k_{PS}}{k_{SP}}} \frac{k_{XPP}}{k_{XS}} \frac{k_{SP}}{k_{PS}} \right|}. \qquad [145]$$

L.1. Minimum drive to reach nonmonotonic phenotypes. In this section, we investigate analytical lessons from our preceding analysis that comment on the behaviors we encountered in our numerical analyses driving two edges in Fig. S17 and Fig. 4 of the main text.

When they are mathematically defined, the critical drive values $\Delta\mu_1$ and $\Delta\mu_2$ are the minimum inputs of drive required to convert a monotonic output to a nonmonotonic output. It is worth remarking that once those critical values are exceeded, nonmonotonicity can persist only for a finite range of drive, because the underlying kinetic conditions—namely, c (Eq. [136]) or d (Eq. [142])—that enable the critical drives to exist are not always satisfied. However, so long as at least one of c or d is always satisfied, $\Delta\mu_1$ and/or $\Delta\mu_2$ are rigorous values for the critical drive the system must maintain to create nonmonotonicity.

Now, we specialize to the case where we may control just one of the four rate constants $(k_{XXP}, k_{SP}, k_{XPX}, k_{PS})$, in addition to some other arbitrarily chosen one. To be concise, denote $x_1 = \frac{k_{XXP}}{k_{SP}}$ and $x_2 = \frac{k_{XPX}}{k_{PS}}$. The first way to access nonmonotonicity is when condition (Eq. [136]) is satisfied, allowing $\Delta\mu_1$ to exist. As long as $x_1 \neq x_2$, this condition c may also be expressed as,

$$c(k_{XXP}, k_{SP}, k_{XPX}, k_{PS}) = \begin{cases} x_1 > 1 \text{ and } x_2 > 1 & \text{or,} \\ x_1 < 1 \text{ and } x_2 < 1. \end{cases} \quad [146]$$

Under this condition, if $x_1 \to x_2$, $\Delta\mu_1 \to 0$ non-monotonicity is reached for any finite drive. When at detailed balance using our estimated biological starting rates, the default values of these governing ratios are $x_{1eq} < 1$ and $x_{2eq} < 1$. Accordingly, if we tune one of the four rate constants that define x_1 or x_2, we can approach the limit where $x_1 \to x_{2eq} < 1$ or $x_2 \to x_{1eq} < 1$, while preserving the necessary conditions for $\Delta\mu_1$ to exist and the response to be nonmonotonic. To compensate, the additional rate constant being tuned can then be adjusted to ensure that asymptotically-little energy is spent, $\gamma \to 1$. This protocol would ensure that an asymptotically-small adjustment of rate constants from such default values would unlock a nonmonotonic output at any nonzero drive. This special starting point is unique for a given pair of rate constants that satisfy this condition, because there are two unknowns (the two rate constants) and two asymptotic equations, namely,

$$\begin{cases} x_1 = \frac{k_{XXP}}{k_{SP}} \to \frac{k_{XPX}}{k_{PS}} = x_2 \\ \gamma \equiv \frac{k_{SX}k_{X,XP}k_{XP,P}k_{PS}}{k_{XS}k_{XP,X}k_{P,XP}k_{SP}} \to 1 \end{cases}. \quad [147]$$

For the remaining six pairs of rate constants that do not include the four rates that define x_1 and x_2, the limit of the minimal drive needed to reach nonmonotonicity is a finite value. In fact, this value is the same minimum drive needed when tuning only one of the two edges among a pair. We call this value $\Delta\mu_0$. Indeed, with the rates at equilibrium we chose, the minimal drive for the output to be non monotonic when energy is injected along one of the four rate constants $(k_{PXP}, k_{XPP}, k_{XS}, k_{SX})$ is the same (also valued at $\Delta\mu_0$).

L.2. Conditions that suffice to forbid nonmonotonicity. Now, consider the cases where neither $\Delta\mu_1$ nor $\Delta\mu_2$ is defined. That is to say, when non-monotonicity cannot be achieved for any input of drive on the system. From the converse of the condition c (Eq. [136]), we can deduce that as soon as one of the following conditions is not satisfied, $\Delta\mu_1$ is not defined,

$$\begin{cases} k_{XPX} = k_{PS} & \text{or,} \\ k_{XXP} = k_{SP} & \text{or,} \\ c_1 \text{ and } c_4 & \text{or,} \\ c_2 \text{ and } c_3. \end{cases} \quad [148]$$

Substituting the meanings of the subconditions c_1 through c_4 expresses these conditions guaranteeing monotonicity as,

$$\boxed{\begin{cases} k_{XPX} = k_{PS}, \text{ or} \\ k_{XXP} = k_{SP}, \text{ or} \\ k_{XXP} > k_{SP} \text{ and } k_{XPX} < k_{PS}, \text{ or} \\ k_{XPX} > k_{PS} \text{ and } k_{XXP} < k_{SP} \end{cases}}. \quad [149]$$

For instance, some of the conditions in Eq. [148] immediately suffice to forbid nonmonotonicity via $\Delta\mu_1$ because the argument of the logarithm in $\Delta\mu_1$'s definition becomes negative. Evaluating the second possible route to reach nonmonotonicity, via $\Delta\mu_2$ and its prerequisite condition d (Eq. [142]), we see the same conditions above suffice to forbid its mathematical definition. In summary, if any of the conditions in Eq. [149] are satisfied, the response function must remain monotonic, even for any nonequilibrium driving on the system.

Notice that these conditions Eq. [149] depend only on four rate constants: the binding and and unbinding rates of the polymerase. These are the same four rate constants that fix both the leakiness and saturation. We illustrate these impacts of tuning ratios of these four rate constants in Fig. S18.

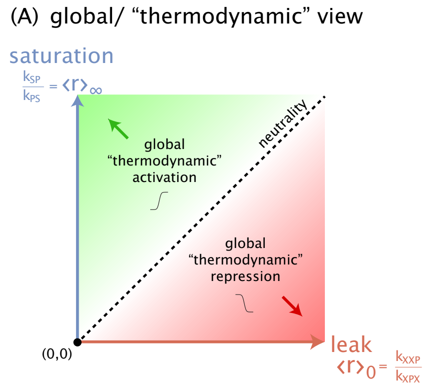

Fig. S18. Nonmonotonic input-output curves are impossible even under any dissipation when certain relationships are obeyed by rate constants. (A): The ratios k_{SP}/k_{PS} and k_{XXP}/k_{kXPX} set whether the transcription factor is globally an activator or repressor (showing a saturation larger (smaller) than the leak, respectively). (B): The ratios k_{XPX}/k_{PS} and k_{XXP}/k_{SP} set whether the curve can ever be nonmonotonic.

As discussed briefly in the main text, some biophysical contexts may, by default, satisfy some of the conditions Eq. [149] guaranteeing nonmonotonic responses. For instance, under the classical assumption that the binding rate of the polymerase is purely diffusion-limited, its on rate would not depend on whether the transcription factor is already bound to the genome or not, enforcing $k_{XXP} = k_{SP}$ and hence forbidding nonmonotonicity by default, even for any drive or modulation of the other rate constants. Manifesting nonmonotonicity departing from these default rates would then require energy investment to break this rate symmetry. This pivotal constraint is plausibly relievable by diverse modes of transcriptional regulation, but emphasizes the privileged roles that some ratios of rate constants have in determining the flexibility of output responses. We illustrate two such symmetries, with different default biological plausibility, in Fig. S19.

For biological reasons, other pairs of rate constants of the system could be equal. Indeed, if the binding of any molecules is only limited by diffusion, the on rates of the transcription factor should also be equal. We observe, and Eq. 5 of the main text reports, that the only equalities between pairs of rate constants that forbid non-monotonicity are the on- or off- rates of the polymerase. For instance, the equality between rates of the transcription factor does not forbid the access to non-monotonicity.

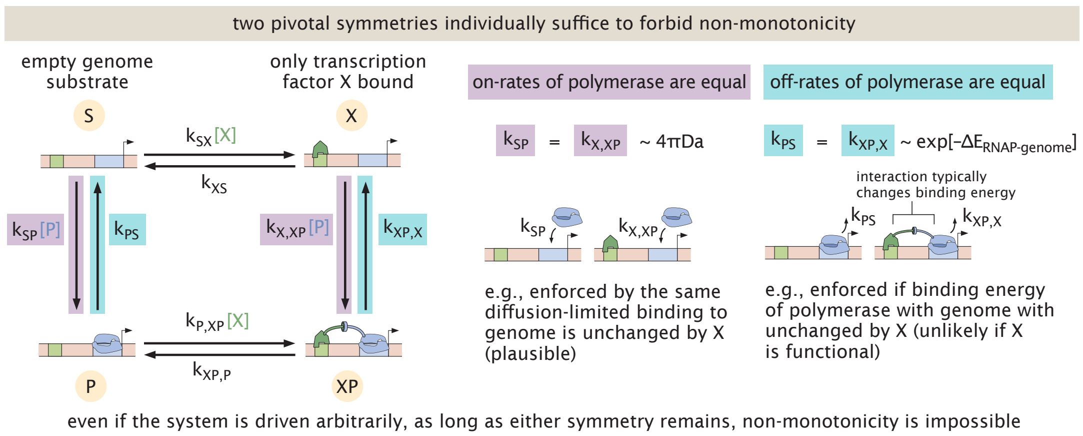

Fig. S19. Physical examples of critical symmetries among rate constants that suffice to forbid nonmontonic responses. One such symmetry occurs when the on-rate of polymerase is unchanged by the presence of the transcription factor, $k_{SP} - k_{X,XP}$ as emphasized in purple shading. Alternatively, when the unbinding-rate of the polymerase is unchanged by the presence of the transcription factor, $k_{PS} = k_{XP,X}$ (highlighted in teal shading), the response must also remain monotonic.

M. Implications of critical symmetry conditions for widespread numerical screens. A common, *a priori* reasonable, tactic to confront the explosion in the number of parameters of kinetic models that can accommodate nonequilibrium (relative to the fewer energetic paramaters of equilibrium) is to restrict parameters within certain ranges or under simplifying functional constraints. These constraints help grapple with the reality that each additional parameter implies an exponential increase in the number of, for example, combinatorially-investigated samples of a model. However, our analytical results highlight how imposing such constraints among parameters can unexpectedly collapse the complexity achievable by a kinetic model into a restricted set of output behaviors.

M.1. Enforced monotonocity of transcriptional responses in a decision theory model of nonequilibrium gene regulation. As a first example, Lammers, Flamholz and Garcia (28) recently performed a study of how energetic and kinetic parameters affect the rate at which information is transferred from inputs to transcriptional outputs in a generic model of transcriptional activation inspired by the Monod-Wyman-Changeux model. This study imposed an apparently-benign constraint of parameters intuitively motivated by assuming that a transcription factor accomplishes activation. Specifically, Lammers *et al.* reasoned that if the transcription factor increases the rate at which the system switches between transcriptionally OFF and ON states (relative to this rate without the transcription factor), as encoded by an interaction term they call $\eta_{ab} > 1$, but also *decreases* the complementary switching rate from OFF to ON (encoded by another interaction term $\eta_{ib} < 1$), then the presence of the transcription factor activates transcription (namely, increases the probability of being in a transcriptionally ON state) ((28) and personal communication). In fact, however, this ($\eta_{ab} > 1$ and $\eta_{ib} < 1$) constraint is sufficient, but *not* necessary, for activation. Instead, a looser constraint—merely that the transcription factor makes the ON to OFF rate slower overall than the OFF to ON rate ($\eta_{ib} < \eta_{ab}$)—is the minimal condition adequate for activation. (Thus, a transcription factor can still ultimately activate transcription even when it increases or decreases both transcriptionally OFF-to-ON and ON-to-OFF rates, as long the former still exceeds the latter.) Further, surprisingly, our analytic reasoning establishes the stricter ($\eta_{ab} > 1$ and $\eta_{ib} < 1$) constraints previously assumed by Lammers and colleagues are precisely among those that suffice to *forbid* nonmonotonic output responses, even for any energy expenditure (see Eq. 5 of the main text, and also Fig. S20).

More specifically, a transcription factor is a net activator when the "leak" transcriptional output $\langle r \rangle_0$ without any transcription factor is less than the "saturation" output $\langle r \rangle_\infty$ at a saturating (say infinite) concentration of transcription factor, as show in Eq. [52]. As discussed earlier in §G.2, when the transcription factor is completely absent, the system cannot be found in any microstate that invokes it, collapsing four states into just the two states devoid of transcription factor. Similarly, when the transcription factor concentration is infinite, the system is never found in the two microstates without the transcription factor, again admitting an (orthogonal) two-state description. In the language of the model of Lammers, Flamholz and Garcia, this implies that the leak $\langle r \rangle_0$ is set by a competition between an ON state with probability $p_{\textcircled{3}}$ and an OFF state with probability $p_{\textcircled{0}}$ (see Fig. S20A, right), where the former transitions to the latter at rate k_i (inhibition rate) and the latter transitions to the former at rate k_a (activation rate), just as in §G.2. Hence,

$$\langle r \rangle_0 = r p_{\textcircled{3}} = r \frac{k_a}{k_a + k_i} = r \frac{1}{1 + \frac{k_i}{k_a}}. \quad [150]$$

Conversely, at saturating transcription factor, the output is set by a competition between an ON state with probability $p_{\textcircled{2}}$ and an OFF state with probability $p_{\textcircled{2}}$ that respectively transition between each other at rates $\eta_{ib} k_i$ and $\eta_{ab} k_a$. So the saturation is

$$\langle r \rangle_\infty = r p_{\textcircled{2}} = r \frac{\eta_{ab} k_a}{\eta_{ab} k_a + \eta_{ib} k_i} = r \frac{1}{1 + \frac{\eta_{ib}}{\eta_{ab}} \frac{k_i}{k_a}}. \quad [151]$$

Overall, these expressions indicate that the transcription factor is a net activator, $\langle r \rangle_0 < \langle r \rangle_\infty$, exactly when $\frac{\eta_{ib}}{\eta_{ab}} \frac{k_i}{k_a} < \frac{k_i}{k_a}$, or namely

$$\text{net activation: } \boxed{\frac{\eta_{ib}}{\eta_{ab}} < 1}. \quad [152]$$

Importantly, this is a *looser* condition than that simultaneously ($\eta_{ib} < 1$ and $\eta_{ab} > 1$), as assumed by Lammers, Flamholz and Garcia (28).

Furthermore, let's define a condition on rate constants for which the regulation unit is an activator at any concentration of transcription factor $[X]$. This is equivalent to $\langle r \rangle([X]) > \langle r \rangle_0, \forall [X] > 0$. We use the expression Eq. [28] for the the rate out of equilibrium. Therefore we want to solve the following inequality $\frac{A+BX+CX^2}{D+EX+FX^2} > \frac{A}{D}$, which simplifies to $(C - \frac{AF}{D})[X] + (B - \frac{EA}{D}) > 0 \forall [X] > 0$. When $[X] \to \infty$, we need $\frac{B}{E} > \frac{A}{D}$ and when $[X] \to \infty$, we need $\frac{C}{F} > \frac{A}{D}$. As the function $f : [X] \to (C - \frac{AF}{D})[X] + (B - \frac{EA}{D})$ is increasing under these assumptions, the condition remains true for all $[X]$. These condition xan be rewritten as $\langle r \rangle_\infty > \langle r \rangle_0$ so $\frac{\eta_{ib}}{\eta_{ab}} < 1$ and $\frac{B}{E} > \langle r \rangle_0$. These condition are still looser than the condition $\eta_{ib} < 1$ and $\eta_{ab} > 1$, assumed by Lammers, Flamholz and Garcia (28) ensures that the system is activating for all concentration $[X]$ but still allows non-monotonicity when we compare them to the conditions of non monotonicity derived in Eq. [90].

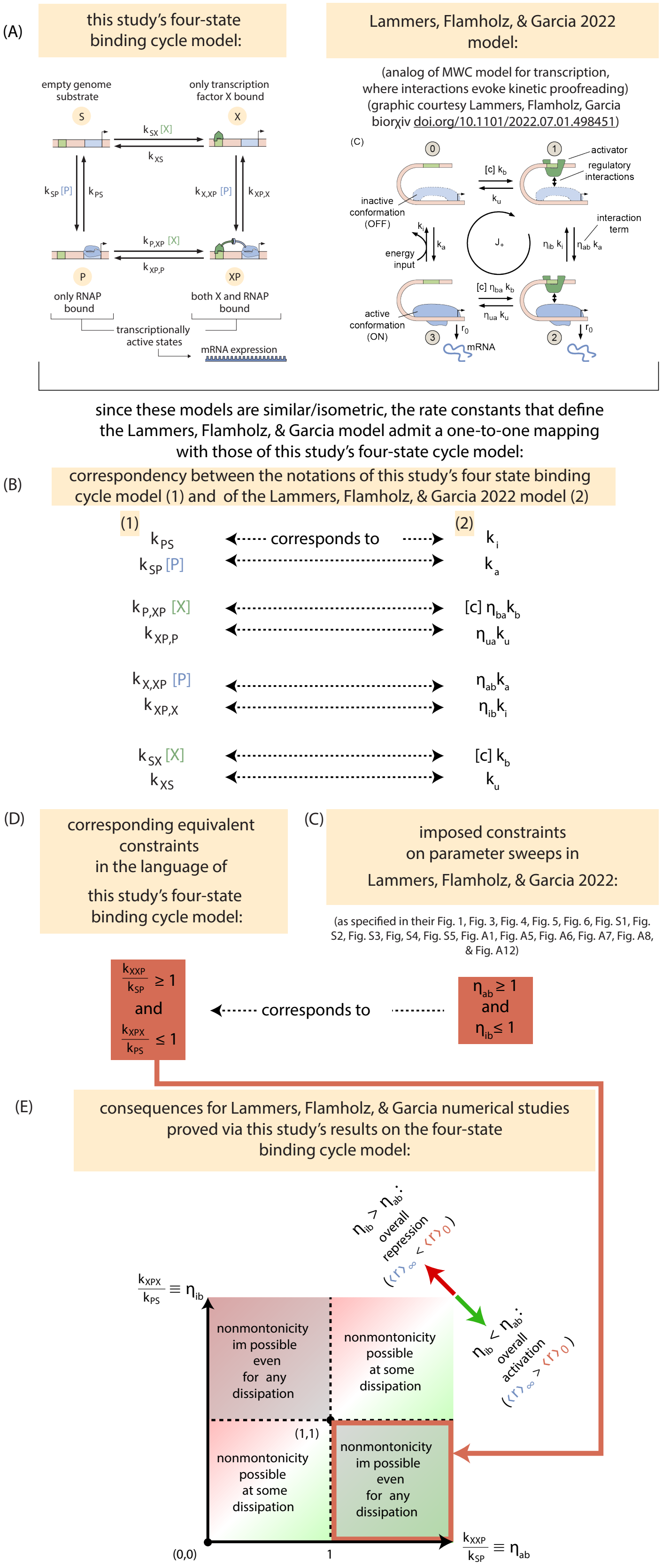

Fig. S20. The simple, analytic conditions we found among rate constants that permit or forbid nonmonotonicity in this four-state system imply strong consequences for the result of large numerical studies by other investigators. Specifically, our conditions imply that a recent study on the relationship between dissipative parameters and the accumulation of transcriptional information (28) admits a hidden/nonobvious restriction implying that all of their input-output curves must be monotonic, for any dissipation. (A): Making the link between our system of interest and the regulation unit studied in reference (28). (B): Establishing the explicit correspondence between the notations used in our setting the ones used in reference (28). (C): Imposed constraints in reference (28) on their parameter sweep, to sample the different output curves. (D): Corresponding equivalent of the constraints summarized in (C) with the notations used in this study. (E): Consequences of the imposed constraints in reference (28) recapitulated in (C) or (D).

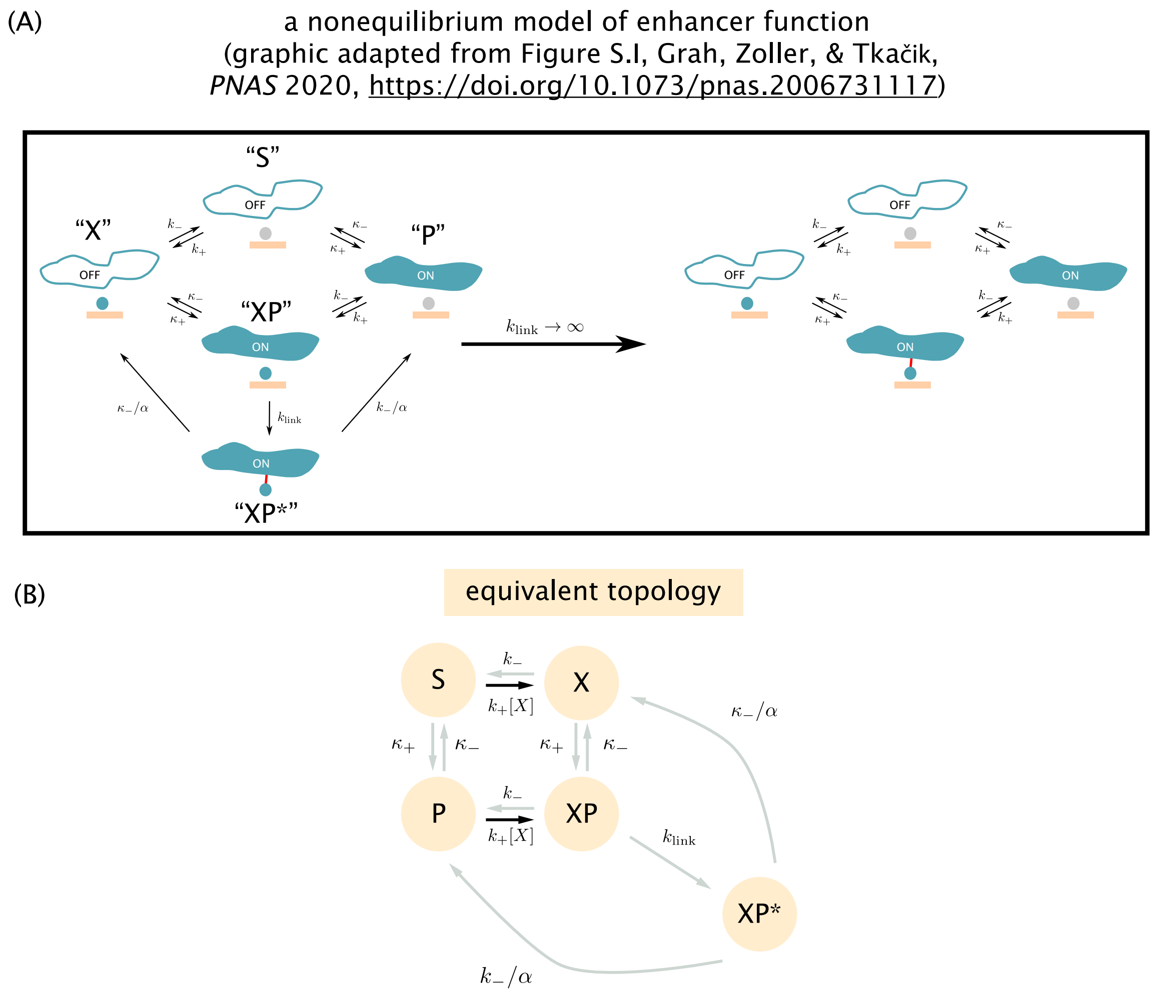

Fig. S21. A nonequilibrium model explored by Grah, Zoller, and Tkačik, (2) contains our square graph as a subgraph, but has extra transitions and states. (A): Figure S.I from reference (2), depicting a model of enhancer, including the four-state graph of this study. The left graph is the most general nonequilibrium model; the right graph (B): Equivalent topology of the graph examined in reference (2). Identifying the equivalents of response coefficients from this topology (beginning in Eq. 154) permit us to formulate analogous kinetic conditions that forbid nonmonotonicity in responses from this model.

M.2. Another recent nonequilibrium transcription model often remains monotonic in enhancer concentration. In Reference (2), Grah, Zoller, and Tkačik assess quantitative features of a nonequilibrium generalization of a MWC model for transcription factors. Their creative work breaks ground on navigating the parametric complexity of the nonequilibrium system by exploring more finely numerical regimes that accomplish desirable quantitative features of transcriptional regulation. Here, by connecting to the nonmonotonicity condition we established while regarding the square graph, we show that kinetic assumptions underlying a number of these numerical parameter sweeps subtly enforce monotonicity in their model's input-output functions, no matter the allowed dissipation. This finding hardly detracts from Reference (2)'s engaging separate purposes exploring quantitative tradeoffs among traits out of equilibrium. However, this setting gives another revealing, simple example of the subtlety of kinetic conditions that permit or forbid nonmonotonicity.

The Grah/Zoller/Tkačik model, pictured in Figure S21, is not a one-to-one mapping to our square graph since their model involves an additional state and extra allowed transitions (creating two extra independent cycles, and four extra coupled cycles). However, by finding the spanning trees of the graph underlying their model, we unsurprisingly find all transcriptional response functions admit up to a quadratic dependence on the enhancer concentration. This observation puts this system under the mathematical purview of our analysis on the mathematically-analogous square graph response.

Specifically, the response observable of interest (the net probability of being in any polymerase-bound states) is here $\langle r \rangle = p_X + p_{XP} + p_{XP^*}$. By finding the coefficients $A, B, C; D, E, F$ in our response function (Eq. 1 of the main text), $\langle r \rangle = \frac{A+B[X]+C[X]^2}{D+E[X]+F[X]^2}$, we evaluate our nonmonotonicity condition on these coefficients we established separately for the square graph. Specifically, we find that

$$A \equiv \sum(\text{trees with } [X]^0\text{-dependence rooted in } X,\ XP,\ \text{or } XP^*) \quad [153]$$

$$= \frac{\kappa_-\kappa_+\left(2k_-^2 + k_{\text{link}}\alpha\kappa_+ + 2k_-(k_{\text{link}}\alpha + \kappa_- + \kappa_+)\right)^2}{\alpha}, \quad [154]$$

and analogously,

$$B \equiv \sum (\text{trees with } [X]^1\text{-dependence rooted in } X,\ XP, \text{ or } XP^*) \quad [155]$$

$$= \frac{(k_+\kappa_+(2\kappa_-(k_-(1+\alpha)+\alpha(\kappa_- + \ \kappa_+)) + k_{\text{link}}\alpha(\kappa_- + \alpha(k_- + \kappa_- + \ \kappa_+))))}{\alpha^2}, \quad [156]$$

$$C \equiv \sum (\text{trees with } [X]^2\text{-dependence rooted in } X,\ XP, \text{ or } XP^*) \quad [157]$$

$$= \frac{k_+^2(k_{\text{link}}\alpha + 2\kappa_-)\kappa_+}{\alpha}, \quad [158]$$

$$D \equiv \sum (\text{all trees with } [X]^0\text{-dependence}) \quad [159]$$

$$= \frac{\kappa_-(\kappa_- + \kappa_+)(2k_-^2 + k_{\text{link}}\alpha\kappa_+ + 2k_-(k_{\text{link}}\alpha + \kappa_- + \kappa_+))}{\alpha^2}, \quad [160]$$

$$E \equiv \sum (\text{all trees with } [X]^1\text{-dependence}) \quad [161]$$

$$= \frac{k_+(k_{\text{link}}\alpha(k_-(\kappa_- + \alpha\kappa_+) + (\kappa_- + \kappa_+)(2\kappa_- + \alpha\kappa_+)) + 2\kappa_-((\kappa_- + \kappa_+)(\kappa_- + \alpha\kappa_+) + k_-(2\kappa_- + \kappa_+ + \alpha\kappa_+)))}{\alpha^2}, \quad [162]$$

$$F \equiv \sum (\text{all trees with } [X]^2\text{-dependence}) \quad [163]$$

$$= \frac{k_+^2(k_{\text{link}}\alpha + 2\kappa_-)(\kappa_- + \alpha\kappa_+)}{\alpha^2}. \quad [164]$$

Now, we deploy the algebraic condition we developed for nonmonotonicity. Previously, we derived this necessary condition for nonmonotonicity in the space of emergent shape parameters, as $a > b$ or $a < 0$. Now, to transact in the $A, B, \ldots, F$ coefficients directly, we express this criterion as $a/b > 1$ or $a < 0$, and substitute

$$a/b \equiv \frac{\frac{B}{E} - \frac{A}{D}}{\frac{C}{F} - \frac{A}{D}}.$$

This gives necessary and sufficient conditions for nonmonotonicity in the Grah/Zoller/Tkačik model, in terms of their rate parameters $\kappa_\pm, k_\pm, \alpha$. For many of their revealing numerical screens, the investigators choose $\alpha > 1, k_{\text{link}} > 0, \kappa_- > 0, \kappa_+ > 0, k_- > 0, k_+ > 0$ (see (2), SI). We find, for instance using `Mathematica`'s Reduce, that these kinetic constraints can *never* satisfy the necessary mathematical condition for nonmonotonicity, no matter the dissipation.

This finding joins the other example we provide from Lammers, Flamholz and Garcia where simplifying kinetic assumptions underlying parameter sweeps subtly limits the quantitative behaviors sampled, inviting the idea that some new quantitative features might be discovered under more relaxed kinetic conditions that permit nonmonotonicity.

3. Discovering generalizations of response behaviors for more complex networks

Here we use the Matrix Tree Theorem to quantify the behaviors of a panoply of important regulatory scenarios beyond the simple square graph, in or out of equilibrium. The same approaches deployed on the square graph yield insights into the output behaviors of larger graphs. We summarize key new network calculations in the Table S2 that follows.

A. Summary of nonequilibrium generalization of important transcriptional motifs.

regulatory architecture	control parameters	network topology	equilibrium behavior, $\langle r\rangle^{\text{eq}}$	general nonequilibrium behavior, $\langle r\rangle$	attained powers in $\langle r\rangle$
simple (strict) repression [*cf.* Bintu (36) case 1]	one: $[X]$		$\frac{A+B[X]}{C+D[X]}$	$\frac{A+B[X]}{C+D[X]}$	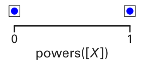
**simple "activation"** (this study's emphasis) [*cf.* Bintu (36) case 2]	one: $[X]$	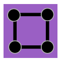	$\frac{A+B[X]}{C+D[X]}$	$\frac{A+B[X]+C[X]^2}{D+E[X]+F[X]^2}$	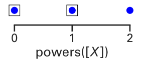
activator recruited by a helper (symmetric) [*cf.* Bintu (36) cases 3 and 7]	two: $[X_1]$, $[X_2]$	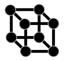	$\frac{A+B[X_1]+C[X_2]+D[X_1][X_2]}{E+F[X_1]+G[X_2]+H[X_1][X_2]}$	$\frac{\sum_{i\le 4, j\le 4, i+j\le 6} A_{ij}[X_1]^i[X_2]^j}{\sum_{i\le 4, j\le 4, i+j\le 6} B_{ij}[X_1]^i[X_2]^j}$	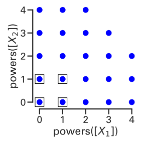
strict repressor recruited by a helper [*cf.* Bintu (36) case 4]	two: $[R]$, $[H]$	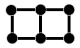	$\frac{A+B[H]+C[R]+D[H][R]}{E+F[H]+G[R]+J[H][R]}$	$\frac{\sum_{i\le 3, j\le 2} A_{ij}[H]^i[R]^j}{\sum_{i\le 3, j\le 2} B_{ij}[H]^i[R]^j}$	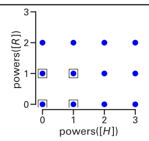
dual strict repressors [*cf.* Bintu (36) cases 5 and 6]	two: $[X_1]$, $[X_2]$	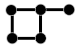	$\frac{A+B[X_1]+C[X_2]+D[X_1][X_2]}{E+F[X_1]+G[X_2]+H[X_1][X_2]}$	$\frac{\sum_{i\le 2, j\le 2, i+j\le 3} A_{ij}[X_1]^i[X_2]^j}{\sum_{i\le 2, j\le 2, i+j\le 3} B_{ij}[X_1]^i[X_2]^j}$	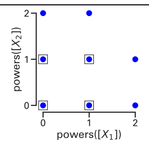
dual activators cooperating via looping[*cf.* Bintu (36) case 8]	two: $[X_1]$, $[X_2]$	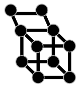	$\frac{A+B[X_1]+C[X_2]+D[X_1][X_2]}{E+F[X_1]+G[X_2]+H[X_1][X_2]}$	$\frac{\sum_{i\le 4, j\le 5, i+j\le 7} A_{ij}[X_1]^i[X_2]^j}{\sum_{i\le 4, j\le 5, i+j\le 7} B_{ij}[X_1]^i[X_2]^j}$	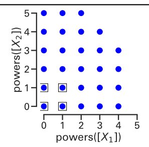
repressor with two DNA binding units and DNA looping [*cf.* Bintu (36) case 9]	one: $[X]$	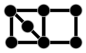	$\frac{A+B[X]+C[X]^2}{D+E[X]+F[X]^2}$	$\frac{A+B[X]+C[X]^2+D[X]^3+E[X]^4}{F+G[X]+H[X]^2+I[X]^3+J[X]^4}$	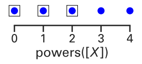
m independent transcription factors [*cf.* spirit of Bintu (36) case 10]	m, $\{[X_i]\}$	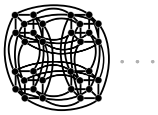 (full hypercube)	$\frac{\sum_{z_1,z_2,z_3,\ldots z_N\in\{0,1\}} \alpha_{\{z_i\}}\prod_{i=1}^{n}[X_i]^{z_i}}{\sum_{z_1,z_2,z_3,\ldots z_N\in\{0,1\}} \beta_{\{z_i\}}\prod_{i=1}^{n}[X_i]^{z_i}}$	$\frac{\sum_{\substack{\max\{i_l\}=i_{(n)}\le 2^{n-1}\\ i_{(n-1)}\le 2^{n-2}\\ \vdots \\ i_{(1)}=\min\{i_l\}\le 2^0}} A_{\{i_l\}}\prod_{i=1}^{m}[X_i]^{i_l}}{\sum_{\substack{\max\{i_l\}=i_{(n)}\le 2^{n-1}\\ i_{(n-1)}\le 2^{n-2}\\ \vdots \\ i_{(1)}=\min\{i_l\}\le 2^0}} B_{\{i_l\}}\prod_{i=1}^{m}[X_i]^{i_l}}$	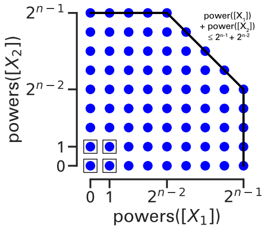

Table S2. Summary of response functions of a variety of important transcriptional regulatory scenarios, generalized here to apply in or out of equilibrium. These examples include generalizing those formerly analyzed by equilibrium statistical mechanics in Reference (36). The rightmost column visualizes the attained powers of the response function (namely, the domains of the sums in the middle two columns); square boxes denote the constrained equilibrium response.

As Table S2 makes clear, the denominator and the numerator of the response $\langle r\rangle$ have the same algebraic form; they differ only on the coefficients of each monomial term. By construction, the coefficients of the polynomial in the denominator (the sum of all rooted and directed spanning trees, behaving like a partition function) are all positive. However, some of the coefficients of the numerator can be zero for a particular observable.

B. The hypercubic graph: regulation by N transcription factors. The empirical distributions of known regulatory architectures for *E. coli* and *D. melanogaster* summarized in §A.1, §A.2, and the main text's Fig. 2A show that fully $\sim$ a quarter to a half of promoters or genes are reported to be regulated by the most common case of just one transcription factor. These scenarios are often plausibly described by a square graph of states and subject to the restrictions and behaviors we explore thoroughly in the main text. Yet clearly, promoters and genes can also be under the control of multiple transcription factors. The presence or absence of these transcription factors on the genome form hypercubic state spaces; some simple examples appear as annotations in Fig. 2A of the main text.

In this section, we derive the mathematical behavior of any response observable defined by these hypercubic networks, in or out of equilibrium. This derivation follows by understanding the strong structural constraints on the possible spanning trees in a hypercube. Following an illustration with small hypercubes and general arguments, Equations 176 and 178 report these generic nonequilibrium and equilibrium behaviors, respectively, thus fully generalizing Equations 1 and 3 of the main text to arbitrary combinatorial binding topologies. Obviously, such more complex network topologies can show more complex and higher-dimensional mathematical behaviors than simple regulatory cycles. However, their flexibility remains constrained, and later in §C we also discuss how biophysical constraints—such as the common scenario of overlapping binding sites—can significantly simplify responses (see Figures S44 and S45).

As new experimental measurements become available, these equations can be used for quantitative fits to gene expression input-output curves, accommodating promoters known to be under the control of many transcription factors. As the contrast between nonequilibrium and equilibrium behaviors grows in higher dimension, these results give tools to discern whether more complicated networks show signatures of operating out of equilibrium. These mathematical behaviors also prelude more specific investigations of attainable sensitivity, newly possible curve shapes, and specific access to interesting regulatory phenotypes, like those found for the square graph in later sections of the main text.

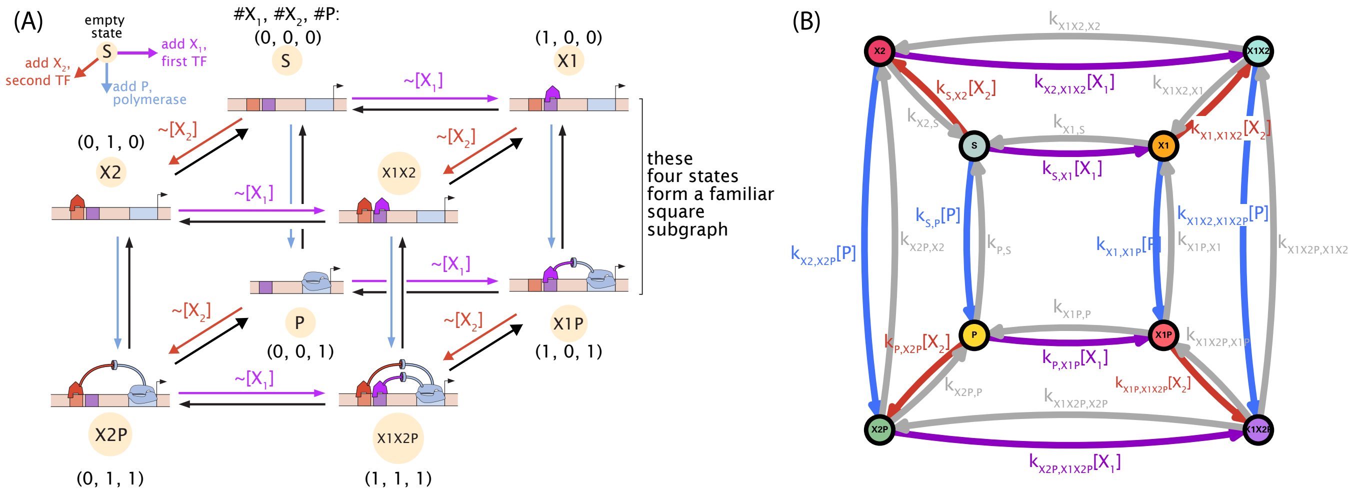

Fig. S22. Regulation with two transcription factor binding sites : a cubic graph. (A): Allowing another transcription factor extends the state space from a square to a cube. In general, for m transcription factors and one polymerase, the state space will become a $n = (m+1)$ dimensional hypercube, where the $2^n = 2^{m+1}$ vertices encode the presence or absence of each of the transcription factors or polymerase. This description assumes that every transcription factor is mutually compatible with the presence of other transcription factors; these transcription factors need not be distinct, if some proteins each have multiple binding sites, in which case some concentrations control multiple sets of orthogonal edges. Here we visualize the $m = 2$ transcription factors case, giving a $n = (2+1)$-dimensional cube. Purple rightwards arrows ↘ illustrate binding with transcription factor X_1; red leftwards arrows ↙ denote binding with transcription factor X_2; and downwards vertical ↓ arrows denote binding with polymerase P. Each state is annotated with the appropriate binary code (a vector of length $n = (m+1)$ whose ith entry is 1 if transcription factor i is present in that state, and 0 otherwise, with the convention that the final $(m+1)$th entry refers to the polymerase). We refer this code to help derive how algebraic responses collapse at detailed balance. (B): The same $n = 2+1$ hypercube-graph , visualized as a planar Tutte embedding, with rates associated labeled.

As a hint to the impending complexity, while we only had sixteen directed spanning trees on the square ($n = 2$-cube), many more trees confront us for hypercube graphs in higher dimensions. In general, the number of undirected spanning trees on the n-cube is,

$$T_u(n) = \frac{1}{2^n} \prod_{i=1}^{n} (2i)^{\binom{n}{i}}. \qquad [165]$$

This enumeration result follows from nontrivial combinatorial arguments (58) or from finding the eigenvalues of the Laplacian by analyzing the vertices of the hypercube as an Abelian group via representation theory (59, § 5.5, p. 62). (These counts of the number of undirected trees on the n-cube are reported in the Online Encyclopedia of Integer Sequences as terms of the sequence OEIS A006237.) Specifically, there are $T_u = 384$ undirected spanning trees on the 3-cube. Since any undirected spanning tree can be rooted at each vertex and generate a unique directed spanning tree, there are $N_{\text{vertices}} = 2^n$ rooted

spanning trees per undirected spanning tree, giving

$$T_d = N_{\text{vertices}} T_u(n) = 2^n T_u(n) = \prod_{i=1}^{n} (2i)^{\binom{n}{i}}$$

directed spanning trees. Or, for $n = 3$, there are $T_d = 2^n T_u = 8 \times 384 = 3{,}072$ directed spanning trees on the 3-cube. This complexity explodes rapidly: the next largest cube, the tesseract ($n = 4$), has a whopping 42,467,328 undirected spanning trees (so 679,477,248 directed spanning trees), and so on. We plot this explosion of rooted spanning trees in Fig. S23.

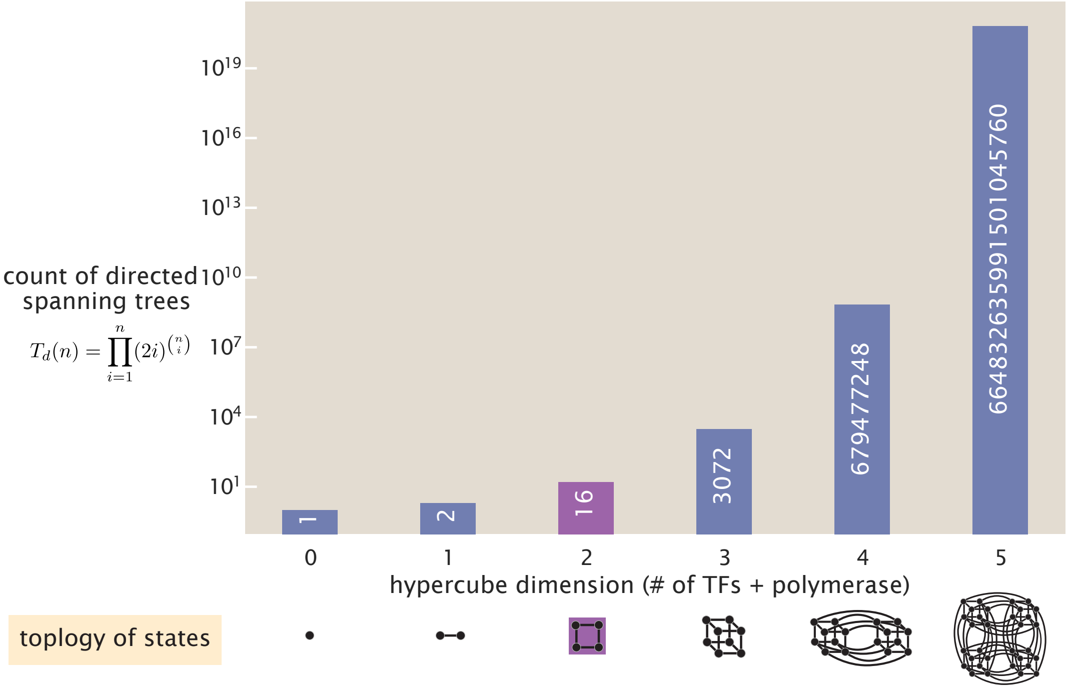

Fig. S23. Superexponential explosion of the number in rooted spanning trees with respect to the hypercube dimension.

Notice that by the time the network is governed by only four transcription factors (forming a hypercube in five dimensions), the number of rooted spanning trees has exploded to over 664 quintillion. This number is over a "millimole" of spanning trees, a supercosmological number: if we managed to speedily sketch 1 spanning tree per second, it would take us $\boxed{\sim 1500\times}$ the current age of the universe to find all these trees. Clearly, brute force must be replaced by the foresight of structural reasoning about these trees.

B.1. Illustration with the $3-$***cube case.*** We first illustrate the consequences of multiple transcription factors with the tractable case of the $n = 3$ dimensional cube, capturing the impact of two transcription factors (call them X_1 and X_2) which can be present at up to one copy each, in addition to the polymerase. This architecture, where appropriate transitions scale with each of these control variables, is depicted in Figures S22.

To enumerate the spanning trees, we exploit the Matrix Tree Theorem: removing row and column i in the Laplacian matrix and taking the determinant gives, up to a sign, the sum of all spanning trees rooted in state i, a procedure amenable to symbolic computation. Figure S24 illustrates the 384 trees rooted in state S, where the node positions are the same as those in Fig. S22. (Figures S34-S40 report the remainder of the 3,072 directed spanning trees comprehensively.)

all trees rooted in S

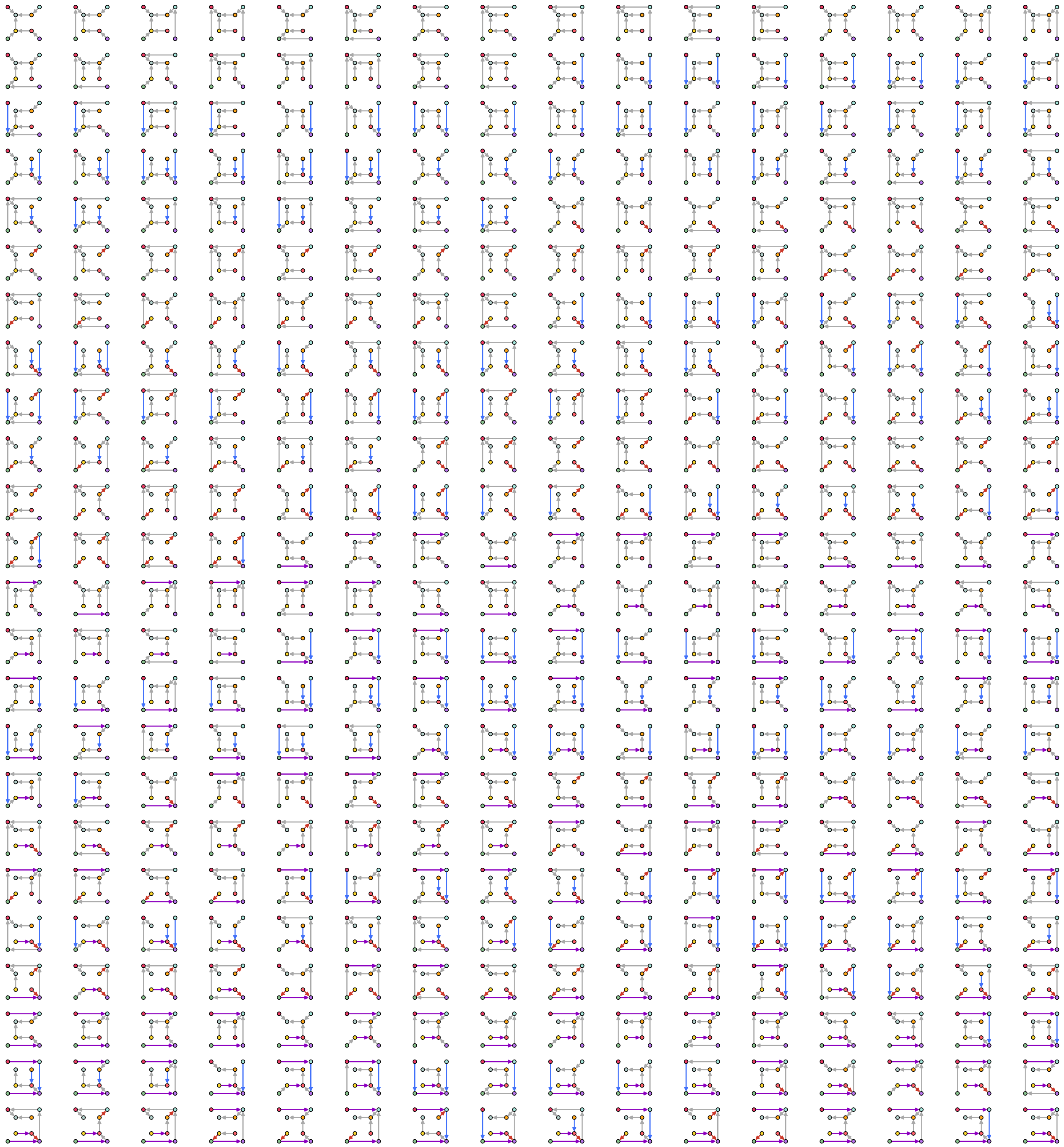

Fig. S24. All directed spanning trees that are rooted in state S, computed by symbolic determinants. The powers of transcription factors $[X_1]$ and $[X_2]$ found in each spanning tree increases from the top left to the bottom right in this grid.

A subset of these trees can be organized into a two-variable analog of our main text's tabular figure 3B, showing how different root states adopt varied dependencies on powers of the control variable(s). We map examples of all attained mixed powers of the transcription factor concentrations for representative states in Figures S24-S28. These dependencies give ρ_{tot}, and therefore any response $\langle r \rangle$, up to a quartic dependence on each control variable, since it is possible to form rooted trees that use all four $[X_i]$-controlled edges present in the graph. However, the graph's structure enforces that not all combinations of powers of the transcription factors are accessible, as we now discuss.

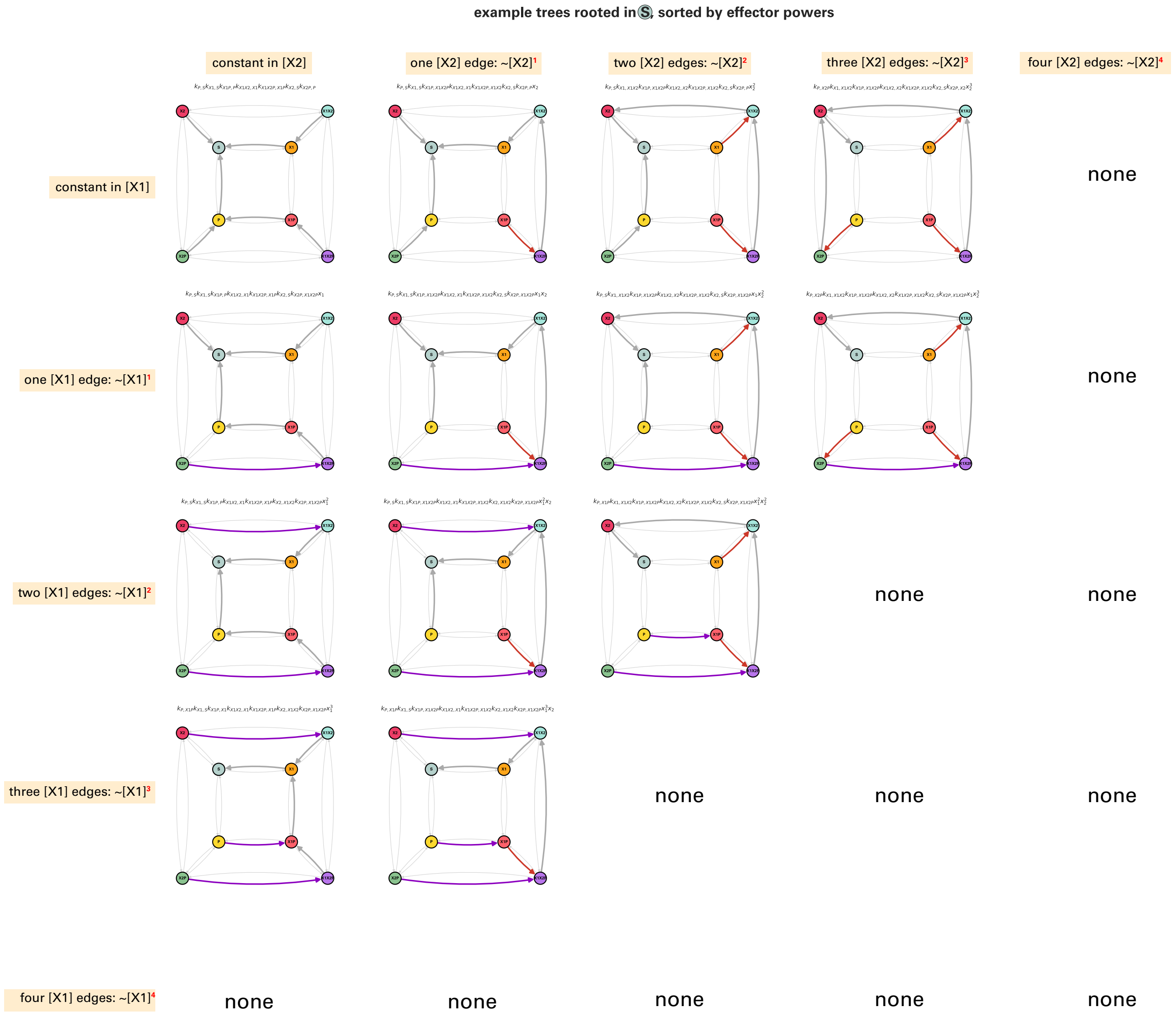

Fig. S25. Examples of spanning trees rooted in the (empty) state S that contribute different combined powers of each transcription factor. Specifically, the entry at row m and column l of the grid exemplifies a spanning tree (when it exists) that has m edges dependent on transcription factor $[X_1]$ and l edges dependent on transcription factor $[X_2]$, contributing a monomial term $\sim [X_1]^m[X_2]^l$ to any response function. This establishes that, for some constants A_{ij} entirely determined by rate constants,

$$\rho_S = A_{00} + \sum_{i=1}^{3} A_{i0}x_1^i + x_2\sum_{i=0}^{3} A_{i1}x_1^i + x_2^2\sum_{i=0}^{2} A_{i2}x_1^i + x_2^3\sum_{i=0}^{2} A_{i3}x_1^i. \qquad [167]$$

(State P attains the same possible powers of $[X_1]$ and $[X_2]$ shown here.)

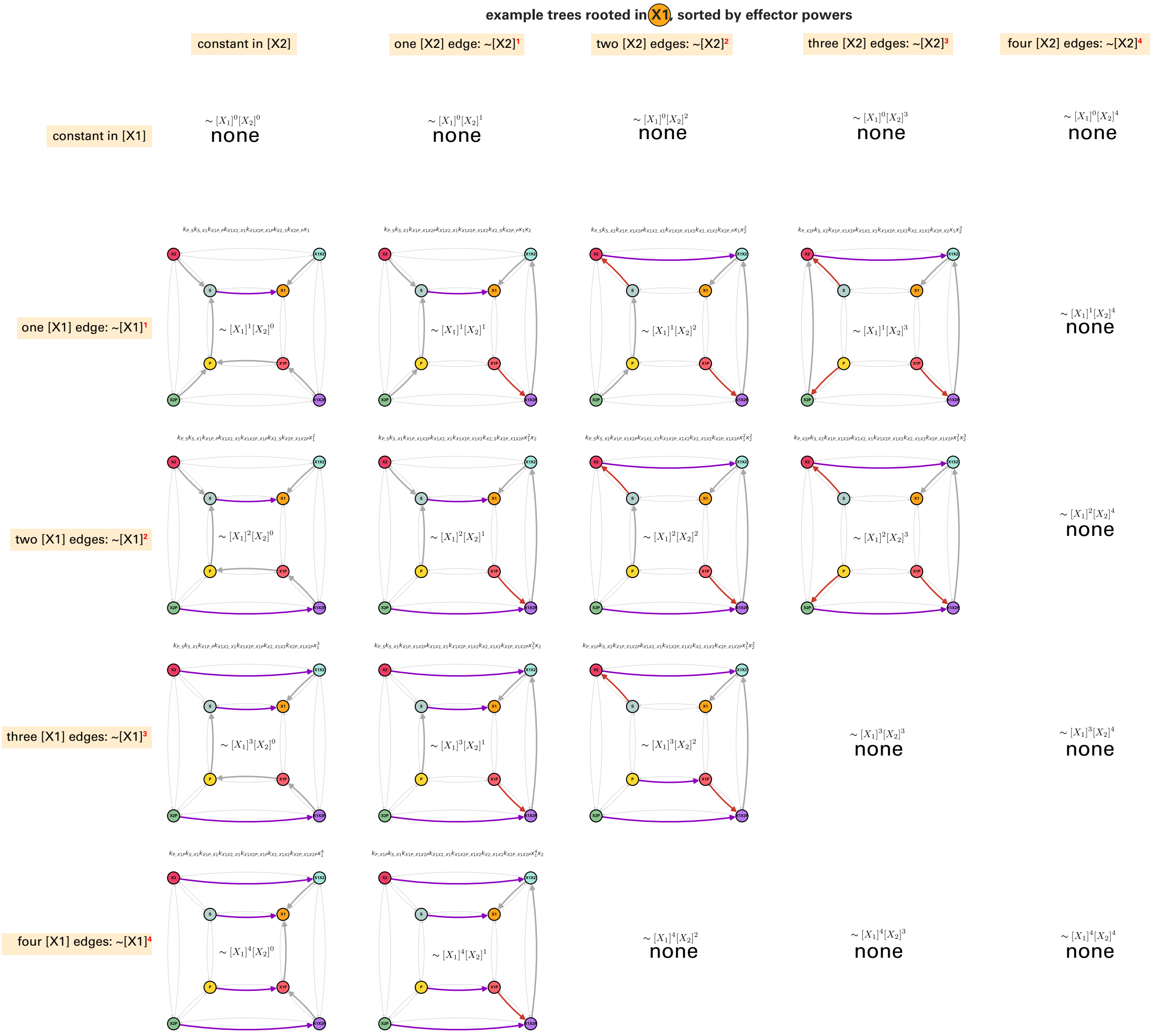

Fig. S26. Examples of spanning trees rooted in the state X_1 that contribute different combined powers of each transcription factor. This establishes that

$$\rho_{X_1} = \sum_{i=1}^{4} A_{i0} x_1^i + x_2 \sum_{i=1}^{4} A_{i1} x_1^i + x_2^2 \sum_{i=1^3} A_{i2} x_1^i + x_2^3 \sum_{i=1}^{2} A_{i3} x_1^i. \qquad [169]$$

(State $X_1 P$ attains the same possible powers of $[X_1]$ and $[X_2]$ shown here.)

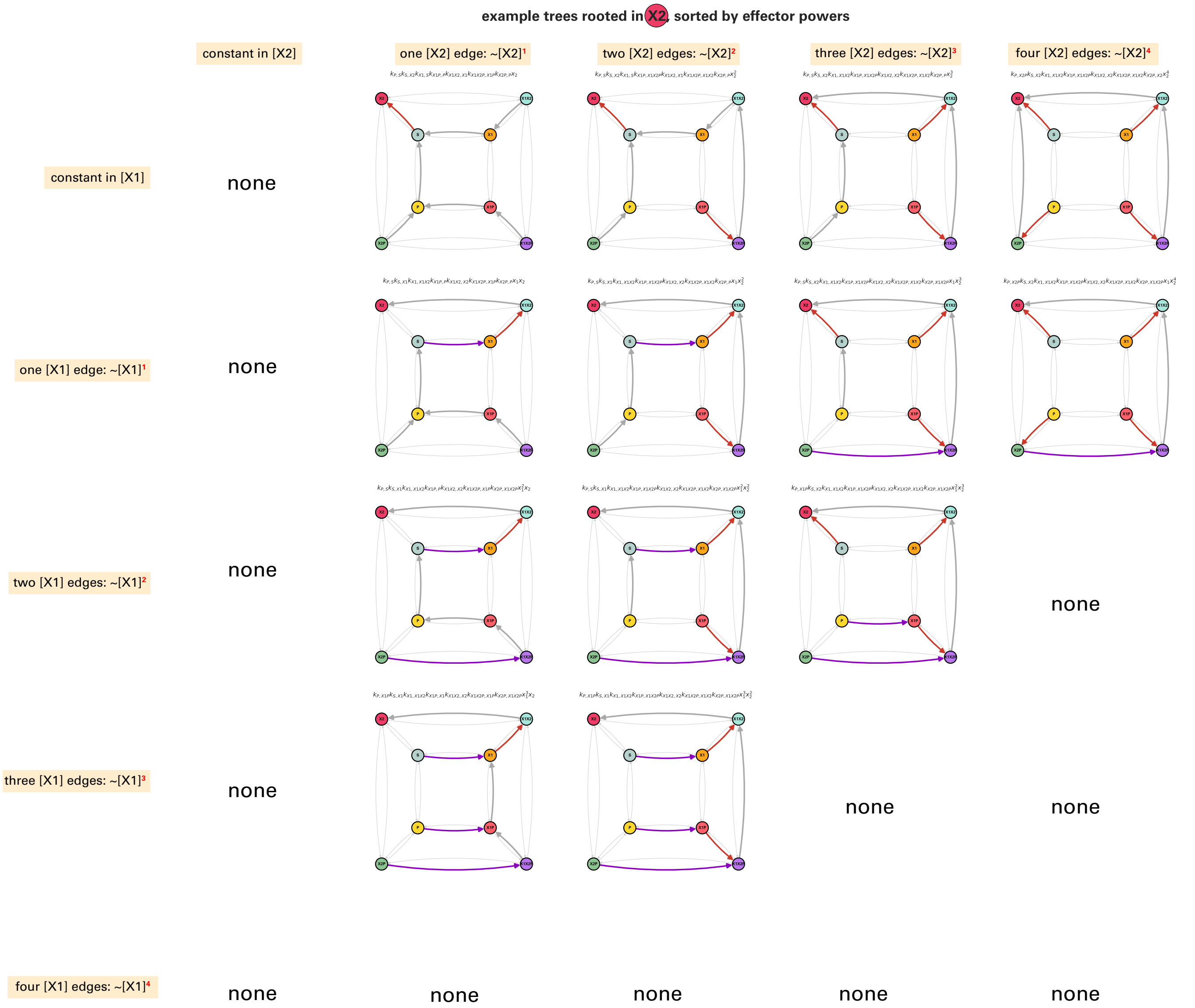

Fig. S27. Examples of spanning trees rooted in the state X_2 that contribute different combined powers of each transcription factor. This establishes that

$$\rho_{X_2} = x_2 \sum_{i=0}^{3} A_{i1} x_1^i + x_2^2 \sum_{i=0}^{3} A_{i2} x_1^i + x_2^3 \sum_{i=0}^{2} A_{i3} x_1^i + x_2^4 \sum_{i=0}^{1} A_{i4} x_1^i. \quad [171]$$

(State X_2P attains the same possible powers of $[X_1]$ and $[X_2]$ shown here.)

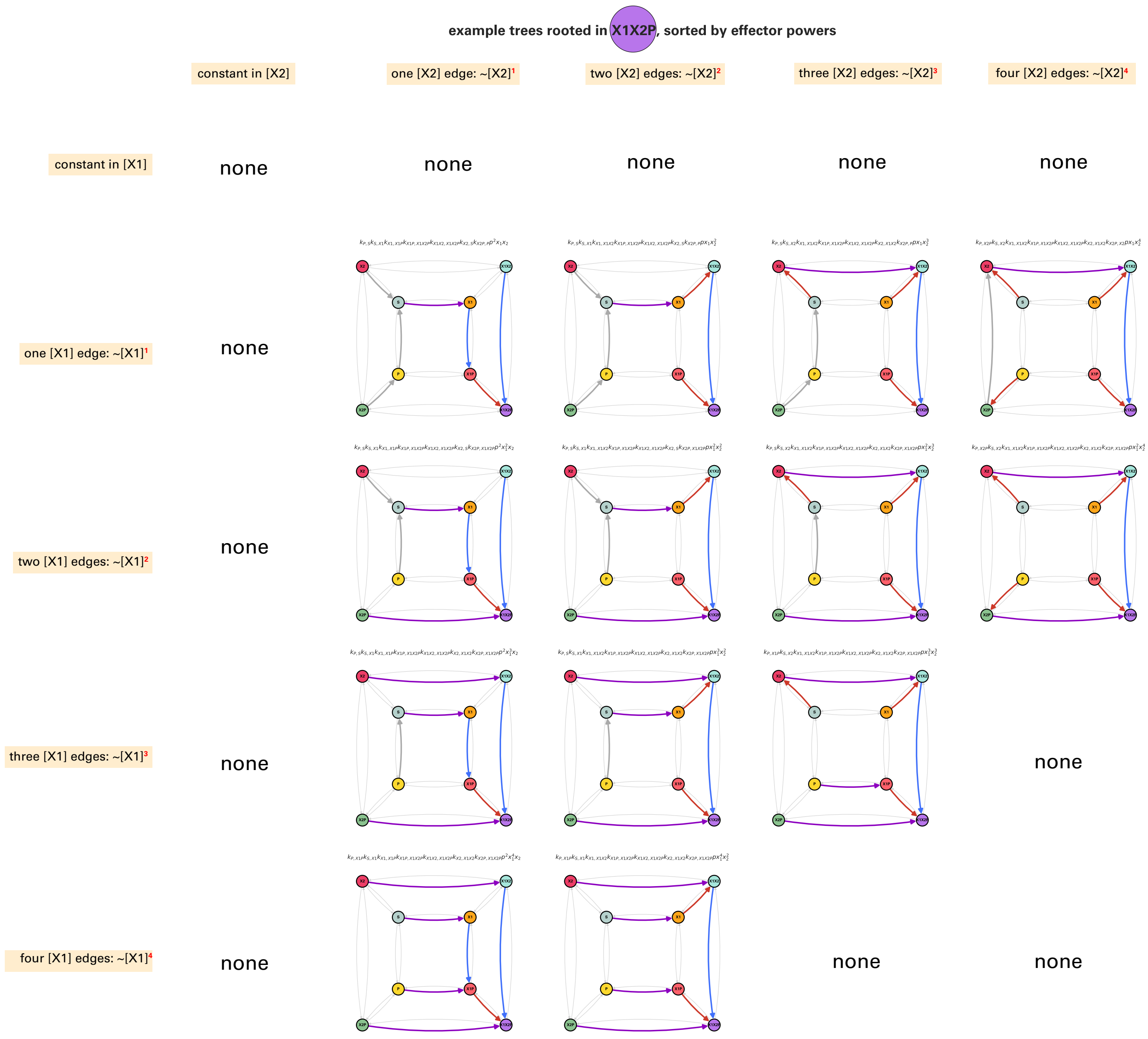

Fig. S28. Examples of spanning trees rooted in the state X_1X_2P that contribute different combined powers of each transcription factor. This establishes that

$$\rho_{X_1X_2P} = x_2 \sum_{i=1}^{4} A_{i1}x_1^i + x_2^2 \sum_{i=1}^{4} A_{i2}x_1^i + x_2^3 \sum_{i=1}^{3} A_{i3}x_1^i + x_2^4 \sum_{i=1}^{2} A_{i4}x_1^i. \qquad [173]$$

(State X_1X_2 attains the same possible powers of $[X_1]$ and $[X_2]$ shown here.)

In summary, these allowed spanning tree structures over all roots give any response (in or out of detailed balance) for the $m = 2$ transcription factors case (two control variables $[X_1], [X_2]$) of the form,

$$\langle r\rangle = \frac{A_{00} + \sum_{i=1}^{4} A_{i0}[X_1]^i + [X_2]\sum_{i=1}^{4} A_{i1}[X_1]^i + [X_2]^2\sum_{i=1}^{4} A_{i2}[X_1]^i + [X_2]^3\sum_{i=1}^{3} A_{i3}[X_1]^i + [X_2]^4\sum_{i=0}^{2} A_{i4}[X_1]^i}{B_{00} + \sum_{i=1}^{4} B_{i0}[X_1]^i + [X_2]\sum_{i=1}^{4} B_{i1}[X_1]^i + [X_2]^2\sum_{i=1}^{4} B_{i2}[X_1]^i + [X_2]^3\sum_{i=1}^{3} B_{i3}[X_1]^i + [X_2]^4\sum_{i=0}^{2} B_{i4}[X_1]^i}. \qquad [174]$$

(Note that while these terms are organized into groups by constant powers of $[X_2]$ for convenience, this ordering is arbitrary: this response is indeed symmetric under interchange of $[X_1]$ and $[X_2]$. The "democratic" structure of the hypercube graph enforces that no transcription factor is special.)

B.2. Slicing the response function when one transcription factor is fixed. Notice that if say the second transcription factor were held fixed at some concentration $[X_2] := [X_2]_0$, the response function is a ratio of quartic polynomials in the remaining transcription factor control parameter. Clearly this response, even with respect to just one control parameter, has acquired new flexibility. Whether a complex experimentally-observed response is attributable to a simple system operating out of equilibrium, or a more architecturally-complex system operating near equilibrium, will depend on specific biological details and separate experimental and biological context—namely, whether many transcription factors' binding sites are indeed found in a promoter and whether these proteins are actually expressed. Nevertheless, such details are often known—or knowable—for genes and promoters of interest.

B.3. Full generalization to the hypercube in n dimensions: cubes within cubes. What explains which transcription factor powers are attained (or not) in this cube graph, and how does this structure generalize to an arbitrary number of transcription factors? To answer this question, it is instructive to consider how a spanning tree could maximize both the numbers of edges controlled by one transcription factor x_1, and those controlled by another x_2, simultaneously.

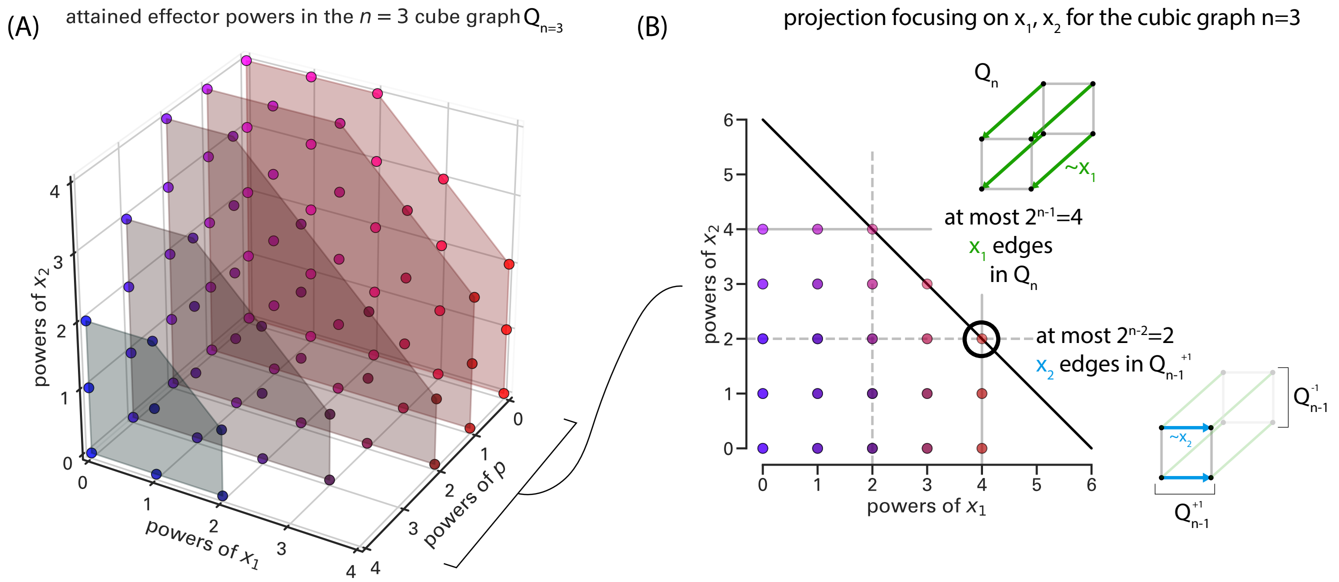

Fig. S29. Powers attained by the transcription factors x_1 and x_2, in addition to the polymerase p, over all spanning trees in the cubic $n = 3$ graph. (A) Powers of $x_1^i x_2^j p^k$ involved in the numerator and denominator of the output function Eq. [174]. If there is a dot at position (i, j, k) in space, then there is a term proportional to $x_1^i x_2^j p^k$ in both numerator and denominator of Eq. [174]. The color of a dot corresponds to a power of p. Pink dots are term with a contribution of order 0 in p, the blue ones of order 5 in p. The plotted planes with the corresponding colors are the planes are constant power of p. (B) Powers of $x_1^i x_2^j$ involved in the numerator and denominator of the output function Eq. [174], not taking into account the polymerase P. (Indeed, this concentration of polymerase is often taken to be constant in regulatory models.) Q_n represents the full hyper-cubic graph of dimension n; in this example, $n = 3$. The edges having an x_1 dependency are represented in green. In a graph representing the binding of two transcription factors, there are at most 2^{n-1} edges depending on the concentration of one of the transcription factors. (In dimension n the hypercube represents the binding of two transcription factors and of the polymerase to the DNA, 2^{n-1} states don't have x_1 bound to the DNA and could acquire it, creating 2^{n-1} edges with a x_1 dependency). In dimension 3 the number of this type of edges is 4. The circled dot represents a spanning tree in which, these 4 edges are participating. As there are 6 nodes in a cube, two edges are still needed to create a spanning tree. The two edges presented in blue, depending on x_2, complete the four green edges, depending on x_1, in a spanning tree. In fact, to complement the 4 green edges into a spanning tree, the two edges have to be in Q_{n-1}^{+1}. Q_{n-1}^{+1} and Q_{n-1}^{-1} are the $n-1$ dimensional cubes formed by states in which X_1 is respectively bound or unbound to the DNA and the edges connecting them. Indeed, the 2 edges have to depart from a node in which X_1 is bound to the DNA (included in Q_{n-1}^{+1} by definition), because the participation edges in Q_{n-1}^{-1} would create nodes with two outgoing edges. In Q_{n-1}^{+1}, there are up to 2^{n-2} edges depending on x_2. Therefore, complementing the 4 green edges, with a x_1 linear dependency, by the two blue edges, with a x_2 linear dependency, creates a spanning tree with a quartic dependency in x_1 and quadratic in x_2, corresponding to the circled dot.

Figure S29 summarizes the attainment of powers of x_1, x_2, and polymerase p, where the powers attained by x_1 and x_2 are emphasized by a projection over the attained polymerase powers at right. A tree on the full (hyper)cube can easily contain all of the possible edges driven by a particular transcription factor, say x_i: there are 2^{n-1} such x_i-driven edges in the whole graph (since half of the 2^n states in a full hypercube lack this particular transcription factor, and from each of these x_i-deprived states we count one x_i-driven edge), explaining how trees can attain powers of $2^{n-1} = 4$ in either x_1 or x_2 individually. (This boundary is visualized by the unbroken grey line in Fig. S29.) However, these trees that contain all of the possible x_i-driven edges cannot simultaneously contain all x_j-driven edges in the graph. This is because, by construction, all of the x_i-deprived states already have exactly one outgoing edge (precisely to assure each of the x_i-driven edges belong to the tree), so the remaining edges that build the spanning tree must be found wholly among the half of the graph's states where x_i is *present*. This restriction halves the number of possible x_j edges that can now participate in the same tree.

In other words, if Q_n represents the full n-dimensional hypercube, then it can always be partitioned into two sets of states, one set deprived of transcription factor x_i and the other enriched by x_i. (In these sets of states, we also consider the interconnecting edges among them.) These sets of states and edges each form an $(n-1)$-dimensional hypercube; call Q_{n-1}^{+i} the subcube where X_i is present, and Q_{n-1}^{-i} the subcube where X_i is absent. The full n-dimensional hypercube is formed by joining these smaller hypercubes together by the perfect matching formed by the set of edges describing the binding of transcription factor X_i. Inside each smaller hypercube Q_{n-1}, there are only 2^{n-2} possible edges driven by the other transcription factor X_j; and demanding that all of the X_i edges appear in a tree restricts the remaining edges to be taken from the Q_{n-1}^{+i} cube of states (where x_i is present). This scenario is illustrated by the example of the power combination circled in black in Fig. S29.

If we relax our demand that *all* 2^{n-1} edges of some transcription factor X_i participate in a tree by allowing one fewer x_i-driven edge to appear, we can find a tree that now includes up to one extra power of another transcription factor X_j; this extra power will come from one of the edges in the Q_{n-1} formed by the X_i-deprived states (formerly forbidden from contributing to the powers of x_j that could participate). Continuing to remove X_i edges continues to add new possible x_j edges in valid spanning trees, up to the 2^{n-1} allowed (by symmetric reasoning). This explains the diagonal boundary of attained powers marked by a black line with slope -1 in the right of Fig. S29; in general, this maximal boundary is a hyperplane assuring that the total powers of transcription factors (and polymerase) is at most

$$\#\text{ of total regulated edges in any tree (hence powers in response)} \leq \sum_{k=0}^{n} 2^{n-k} = 2^n - 1. \qquad [175]$$

For instance, Figure S30 visualizes this planar constraint that powers of $[X_1], [X_2]$, and P must ultimately sum to less than or equal to $2^3 - 1 =$ seven regulated edges in the $n = 3$-cube.

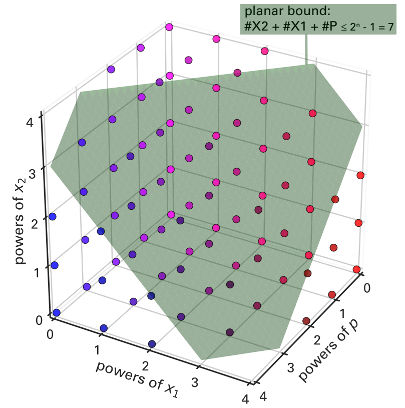

Fig. S30. Visualization of how the plane $\#X_2 + \#X_1 + \#P \leq 2^n - 1 = 7$ (plotted in green) bound the attainable powers in any tree (hence response), in the $n = 3$-cube case. $\#X_2$, $\#X_1$ and $\#P$ respectively correspond to exponent of x_2, x_1 and p. Powers of $x_1^i x_2^j p^k$ involved in the numerator and denominator of the output function Eq. [174]. If there is a dot in the (i, j, k) space, then there is a term proportional to $x_1^i x_2^j p^k$ in both numerator and denominator of Eq. [174]. The color of a dot corresponds to a power of p. Pink dots are term with a contribution of order 0 in p, the blue ones of order 5 in p.

Furthermore, this logic applies recursively to each *subsequent* transcription factor $x_k \notin \{x_i, x_j\}$—for instance, this structure gives trees of maximal power $x_i^{2^{n-1}} x_j^{2^{n-2}} x_k^{2^{n-3}} \cdots$. This reasoning is agnostic to the dimensionality of the state space (total number of transcription factors). Since the maximal power over all transcription factors is at most 2^{n-1}, the second highest power over transcription factors is 2^{n-2}, and so on, we use the notation of order statistics to write the general form. Recall that the ith *order statistic* $y_{(i)}$ of a set of values $\{Y_1, Y_2, \ldots, Y_n\}$ refers to the value that occupies the ith position when the set of values is arranged in ascending order. In other words, it represents the ith smallest value in the set, so the ith largest value of a set is $y_{(n-i)}$. Using this notation, the generic response of any observable (in or out of detailed balance) formed by the hypercubic graph from n transcription factors can be written as,

$$
\langle r \rangle = \frac{\displaystyle\sum_{\substack{\max\{i_l\}=i_{(n)}\le 2^{n-1},\ i_{(n-1)}\le 2^{n-2},\\ \dots\ i_{(1)}=\min\{i_l\}\le 2^0}} A_{\{i_l\}} \prod_{i=1}^{m} [X_i]^{i_l}}{\displaystyle\sum_{\substack{\max\{i_l\}=i_{(n)}\le 2^{n-1},\ i_{(n-1)}\le 2^{n-2},\\ \dots\ i_{(1)}=\min\{i_l\}\le 2^0}} B_{\{i_l\}} \prod_{i=1}^{m} [X_i]^{i_l}}, \qquad [176]
$$

where we have temporarily adopted the convention that one of the "transcription factor"s (say the last one $[X_n]$) represents the polymerase. (Note that this response form obeys the interchange symmetry required of all the transcription factors.)

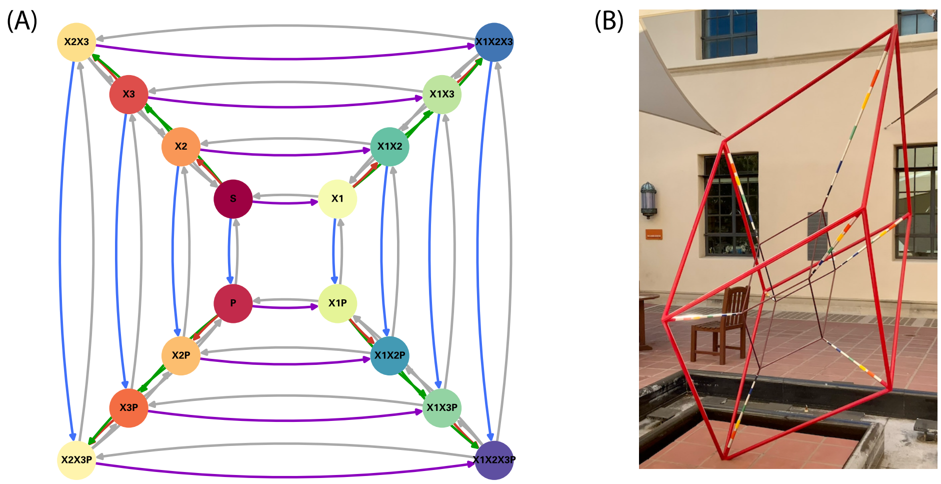

Fig. S31. Adding a third transcription factor X_3 forms a hypercubic state space in $n = 4$ dimensions, known as a tesseract, represented in a concentric layout in panel (A). Panel (B) is a sculpture showing another popular arrangement of nodes in the tesseract, from artist Ronald Linde, pictured at Caltech.

B.4. Further illustration with $n = 4$ ***tesseract graph.*** Explicit enumeration of all 679,477,248 *directed* spanning trees for the 4-cube (tesseract) graph (giving the state space of three transcription factors acting in concert with the polymerase) is not very computationally tractable while preserving all symbolic dependencies on the 32 rate constants associated with the graph. However, we restrict our ambition to discerning just the algebraic form of the x_i dependencies, by replacing all of the rate constant prefactors by 1 but preserving the x_i-dependencies on the rates. This simplifies the symbolic computation of the determinant substantially, enough that *Mathematica* (but still no longer *Python* via `sympy` on consumer computing hardware) can now perform the computation, and reveal the powers attained for all transcription factors x_i.

We visualize the powers attained in this graph in Figure S32. These terms are consistent with the structure of the generic response form we indicate in Eq. 176.

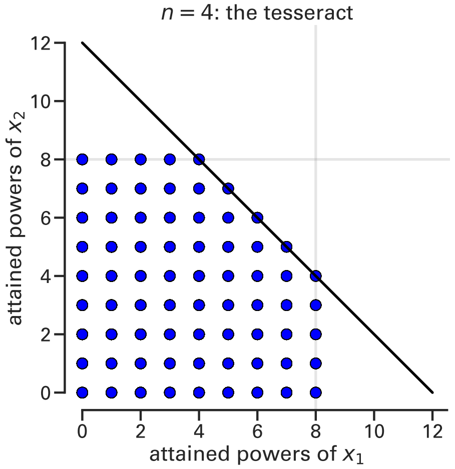

Fig. S32. Powers of $x_1^i x_2^j$ involved in the numerator and denominator of the output function of a tesseract graph (hypercube for $n = 4$), representing a regulation unit with three transcription factor binding sites and depicted in Figure S31. x_2 and x_1 are the respective concentrations of two transcription factor.If there is a dot in the (i, j) plane, then there is a term proportional to $x_1^i x_2^j$ in both numerator and denominator of the output function, at constant x_3 and p (concentration of the third transcription factor and the polymerase). In this graph there are $\sim 6.8 \times 10^8$ spanning trees dependent on x_1, x_2, x_3, p.

B.5. Collapse of the output function at detailed balance. Earlier, in the square graph ($n = 2$-cube), we originally arrived at the response's demotion to a ratio of linear polynomials at detailed balance by explicitly using the cycle condition (namely, that the product of rate constants in one direction around the cycle equals that around the opposite direction at equilibrium). However, now for the $n = 3$-cube, we have no fewer than twelve coupled cycles, complicating a direct translation of our earlier tact to such more complicated graphs. Instead of explicit appeals to cycle conditions, we use the alternative structural reasoning we used in § of this SI.

By emphasizing the powers of control parameter accumulated along paths between states that are disconnected, this reasoning helps us understand how the $n = 3$-cube demotes its response at detailed balance. As before, we are essentially interested in expressing a given state j's statistical weight ρ_j relative to the statistical weight of a reference state (call it P again without loss of generality). The state P is directly connected to states $\{S, X_1P, X_2P\}$, so these terms contribute at most a pure linear power $[X_1]$ or $[X_2]$ to any responses. This leaves the remaining, disconnected states: $\{X_2, X_1, X_1X_2P\}$ are two edges away from P in the graph, and state X_1X_2 is a full graph diameter of three edges away from P. While it *is* possible to create paths between P and these other states that involve two edges dependent on an individual control parameter, actually this does *not* imply that the response is demoted to only a quadratic polynomial in that parameter, since all of these paths also must contain edges scaling as the inverse of that control parameter, canceling at least one power of this dependence.

This cancellation immediately becomes more clear when we return to the binary encoding of states we mentioned in Fig. S22. Every state in the hypercube graph, no matter how many edges apart, differs from any other state by at most a negation in each of its binary digits. When comparing two states in the graph, a negation in a particular binary coordinate i is exactly a difference in whether or not transcription factor i is present or absent between the two states. Since every edge at most changes one binary coordinate at a time, this just says that the net number of binding versus unbinding events of transcription factor i along any path connecting two states is at most the maximum binary distance along a binary coordinate possible allowed by the graph, namely one. Summarizing in other words, even if two states are connected by a long path of possibly $[X_i]$−dependent arrows, the simple state space requires that every such path ultimately contribute at most a factor $[X_i]^{\pm 1}$ to the ratio of the statistical weights of the two states.

To wit, the response of a hypercube graph formed by the interaction of N transcription factors $\{X_i\}_{i=1:N}$ must ultimately collapse at equilibrium to the dramatically simplified multilinear form,

$$\langle r \rangle^{\text{eq.}} = \boxed{\frac{\displaystyle\sum_{z_1, z_2, z_3, \ldots z_N \in \{0,1\}} \alpha_{z_1, z_2, z_3, \ldots z_N} \; [X_1]^{z_1} [X_2]^{z_2} [X_3]^{z_3} \cdots [X_n]^{z_n}}{\displaystyle\sum_{z_1, z_2, z_3, \ldots z_N \in \{0,1\}} \beta_{z_1, z_2, z_3, \ldots z_N} [X_1]^{z_1} [X_2]^{z_2} [X_3]^{z_3} \cdots [X_n]^{z_n}}} \qquad [177]$$

$$= \frac{\displaystyle\sum_{z_1, z_2, z_3, \ldots z_N \in \{0,1\}} \alpha_{z_1, z_2, z_3, \ldots z_N} \prod_{i=1}^{n} [X_i]^{z_i}}{\displaystyle\sum_{z_1, z_2, z_3, \ldots z_N \in \{0,1\}} \beta_{z_1, z_2, z_3, \ldots z_N} \prod_{i=1}^{n} [X_i]^{z_i}}. \qquad [178]$$

For our $n = 3$ (two-transcription factor) example, the detailed balance response has the form,

$$\langle r \rangle^{\text{eq.},\ m=2} = \frac{\alpha_{111}[X_1][X_2]p + \alpha_{110}[X_1][X_2] + \alpha_{100}[X_1] + \alpha_{010}[X_2] + \alpha_{001}p + \alpha_{000}}{\beta_{111}[X_1][X_2]p + \beta_{110}[X_1][X_2] + \beta_{100}[X_1] + \beta_{010}[X_2] + \beta_{001}p + \beta_{000}}. \quad [179]$$

In this example, if e.g. $[X_2]$ is held at some constant, then this form collapses dramatically all the way back down to a ratio of linear polynomials in $[X_1]$. This collapse is still true in n dimensions: if all but a specific transcription factor's concentration $[X_i]$ were held fixed, then the functional form with just this non-fixed control variable is still just a ratio of linear polynomials in $[X_i]$.

In summary, we have shown how the response function attains a restricted subset of possible combinatorial powers of control variables, in or out of equilibrium, on the n-cubic graph. We graphically summarize the algebraic constraints on these responses in Figure S33, which shows the attainable powers of any two control variables among the full n-space.

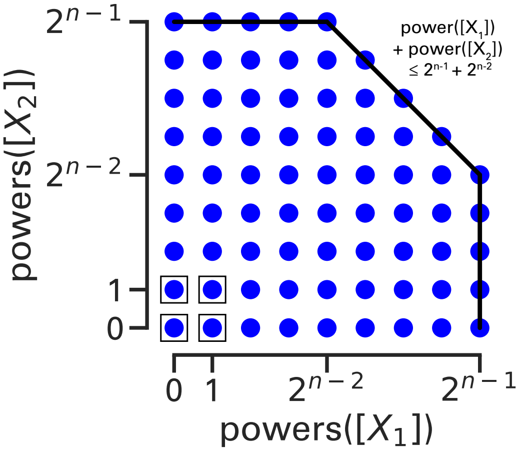

Fig. S33. Graphical summary of how any two control variables $[X_1]$ and $[X_2]$ participate algebraically in the nonequilibrium or equilibrium response on an n-cubic graph. If a blue dot appears at position (i, j), then a monomial of the form $[X_1]^i[X_2]^j$ participates in the numerator and denominator of the algebraic form of the response $\langle r \rangle$. The dots outlined by the black boxes are the only powers attainable when the response is at equilibrium. The boundary of the permitted jointly-attained powers is a result of the structural reasoning explained in this section.

B.6. Accommodating multiple binding sites for a transcription factor. The general response function for the hypercubic graph, derived in Eq. 176 and Eq. 178, accommodates equally well the cases where all transcription factors bound to the promoter are molecularly distinct, and the cases where there are multiple copies of a given transcription factor bound to the promoter. For instance, if one transcription factor X_j has two binding sites (and thus can be found at a copy number of zero, one, or two copies on the promoter), against a backdrop of m other transcription factors with single binding sites, then the response function is given by Eq. 176 and Eq. 178 for a $(m + 2)$-dimensional hypercube, except that two concentrations appearing in these response formulas have been set to the same value $[X_j]$.

mini-Appendix: All spanning trees in the $n = 3$-cube graph over other roots.

all trees rooted in X1

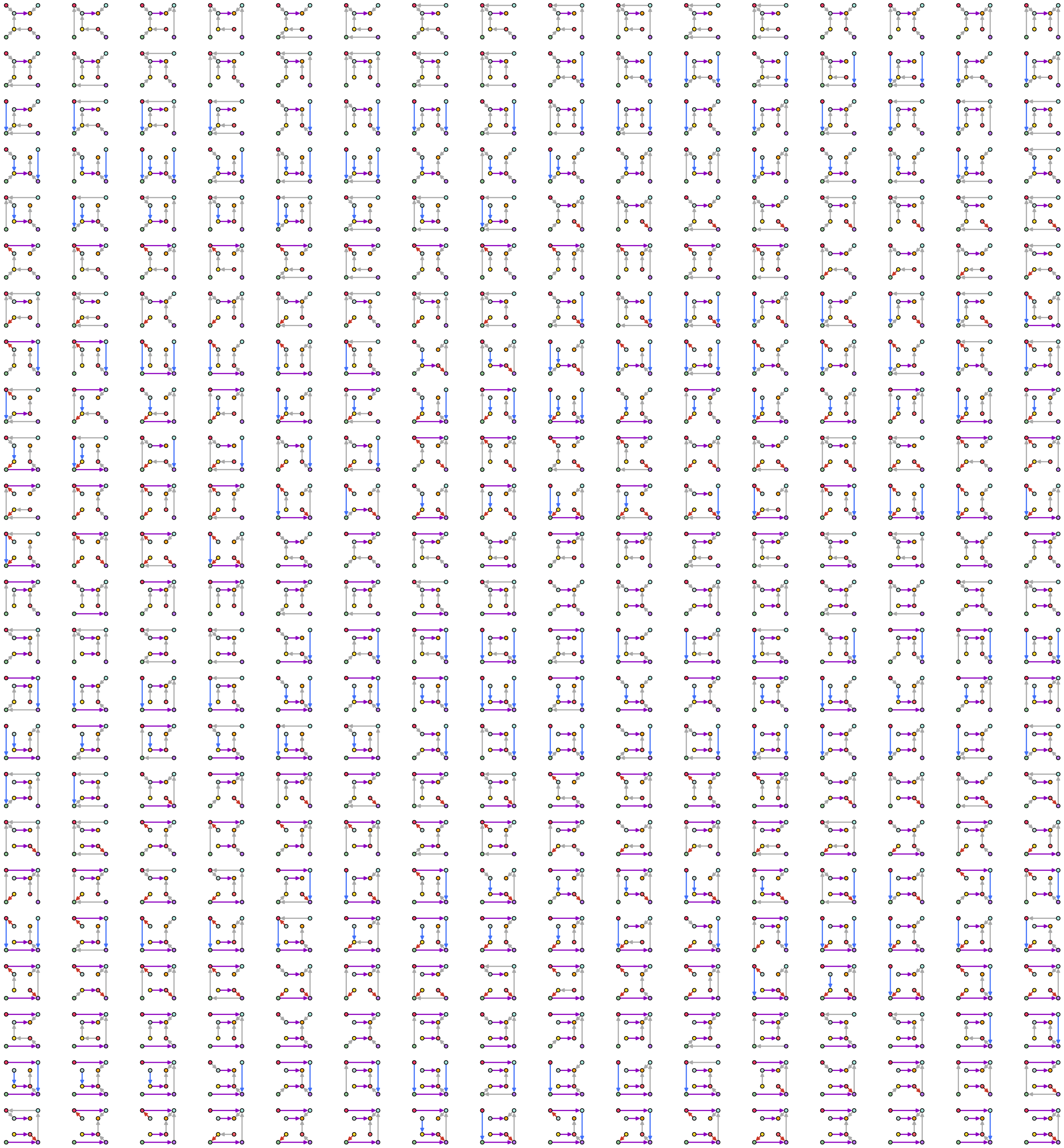

Fig. S34. All directed spanning trees that are rooted in state X_1 for a cubic graph, representing a regulation unit with two transcription factor binding sites. The powers of transcription factors $[X_1]$ and $[X_2]$ found in each spanning tree increases from the top left to the bottom right in this grid. $[X_1]$ and $[X_2]$ are the concentration of the two transcription factors and X_1 is the state in which only the transcription factor X_1 is bound to the DNA.

all trees rooted in X1P

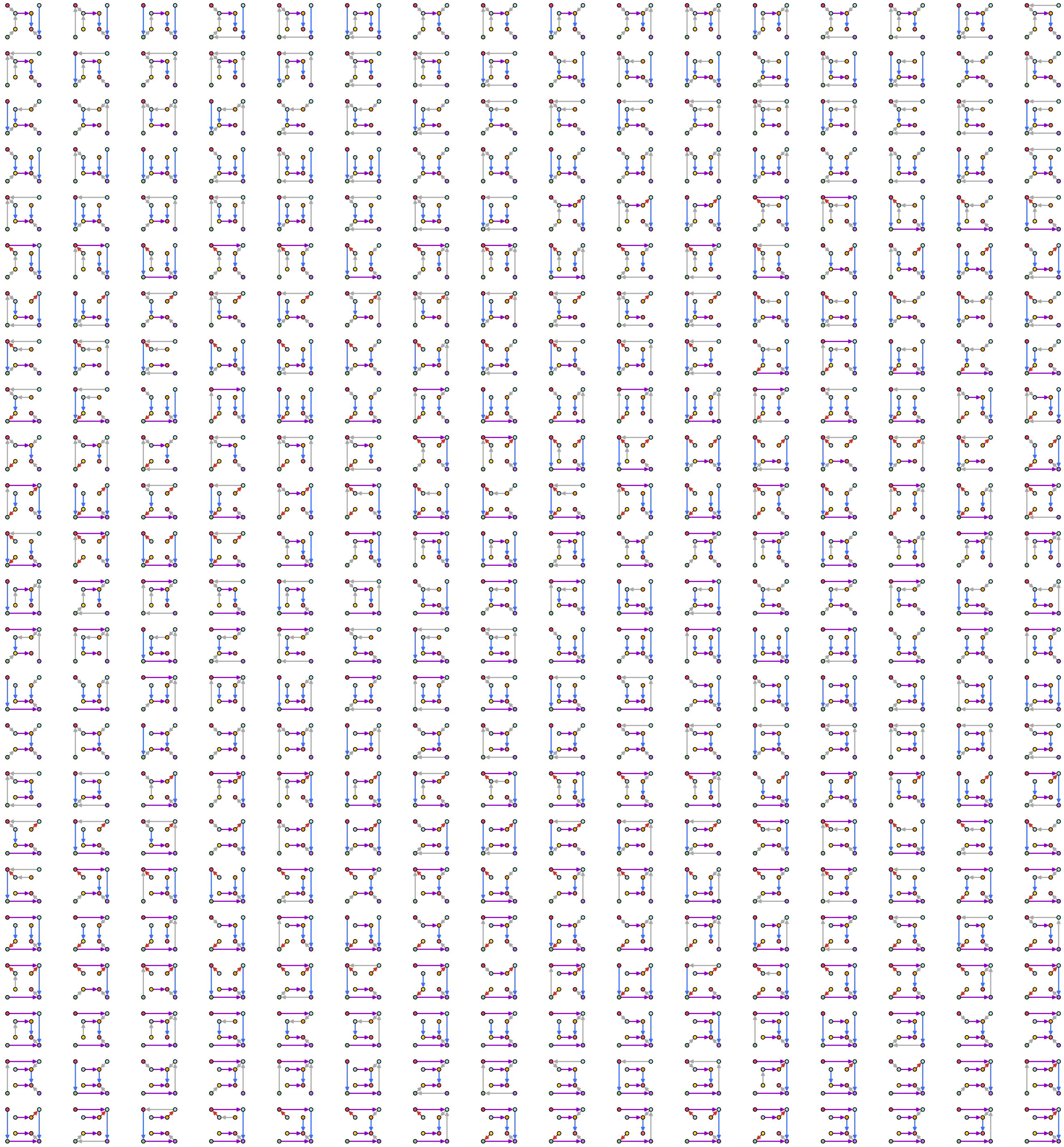

Fig. S35. All directed spanning trees that are rooted in state X_1P for a cubic graph, representing a regulation unit with two transcription factor binding sites. The powers of transcription factors $[X_1]$ and $[X_2]$ found in each spanning tree increases from the top left to the bottom right in this grid. $[X_1]$ and $[X_2]$ are the concentration of the two transcription factors and X_1P is the state in which only the transcription factor X_1 and the polymerase P are bound to the DNA.

all trees rooted in P

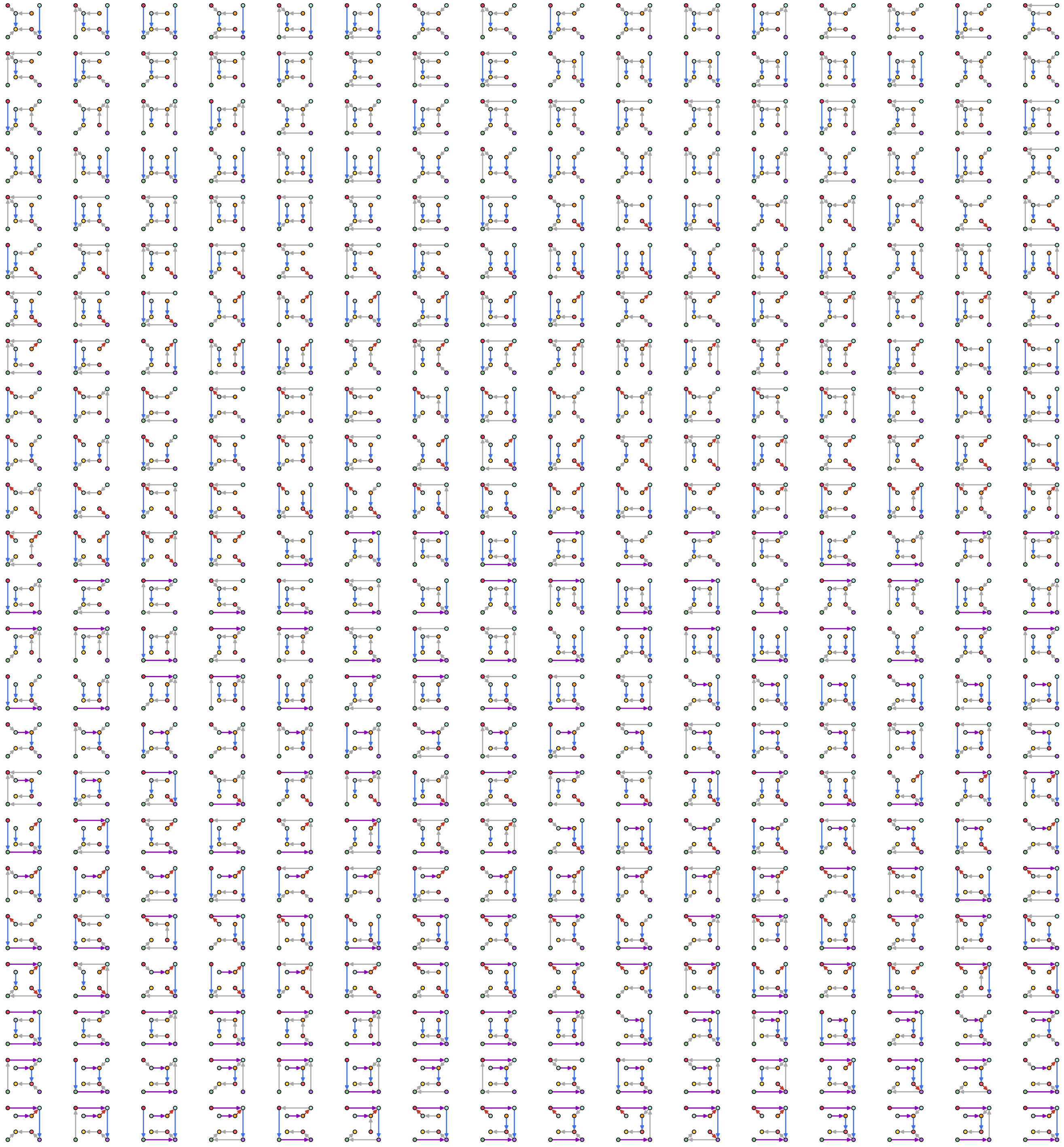

Fig. S36. All directed spanning trees that are rooted in state P for a cubic graph, representing a regulation unit with two transcription factor binding sites. The powers of transcription factors $[X_1]$ and $[X_2]$ found in each spanning tree increases from the top left to the bottom right in this grid. $[X_1]$ and $[X_2]$ are the concentration of the two transcription factors and P is the state in which only the polymerase P is bound to the DNA.

all trees rooted in X2

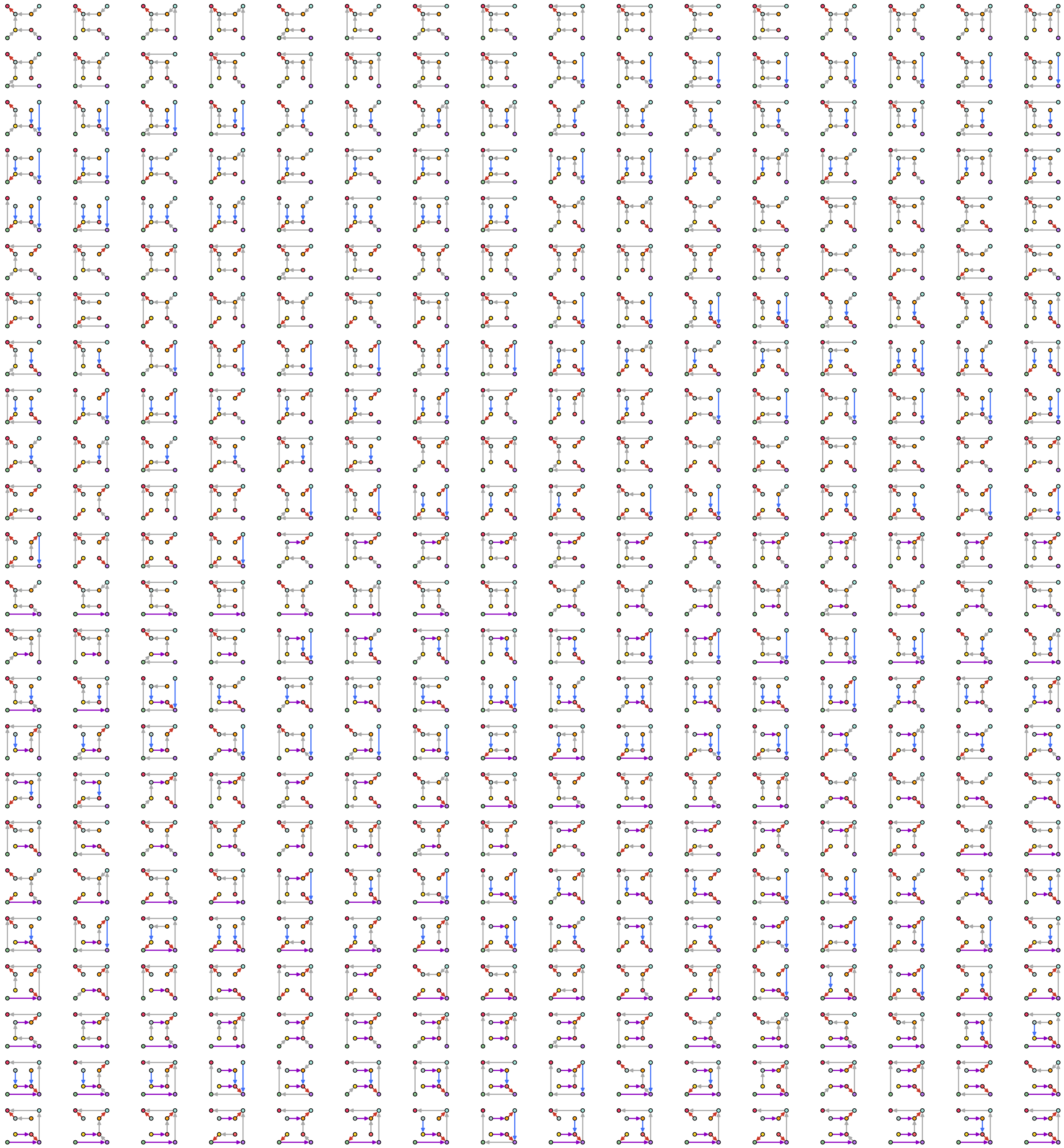

Fig. S37. All directed spanning trees that are rooted in state X_2 for a cubic graph, representing a regulation unit with two transcription factor binding sites. The powers of transcription factors $[X_1]$ and $[X_2]$ found in each spanning tree increases from the top left to the bottom right in this grid. $[X_1]$ and $[X_2]$ are the concentration of the two transcription factors and X_2 is the state in which only the transcription factor X_2 is bound to the DNA.

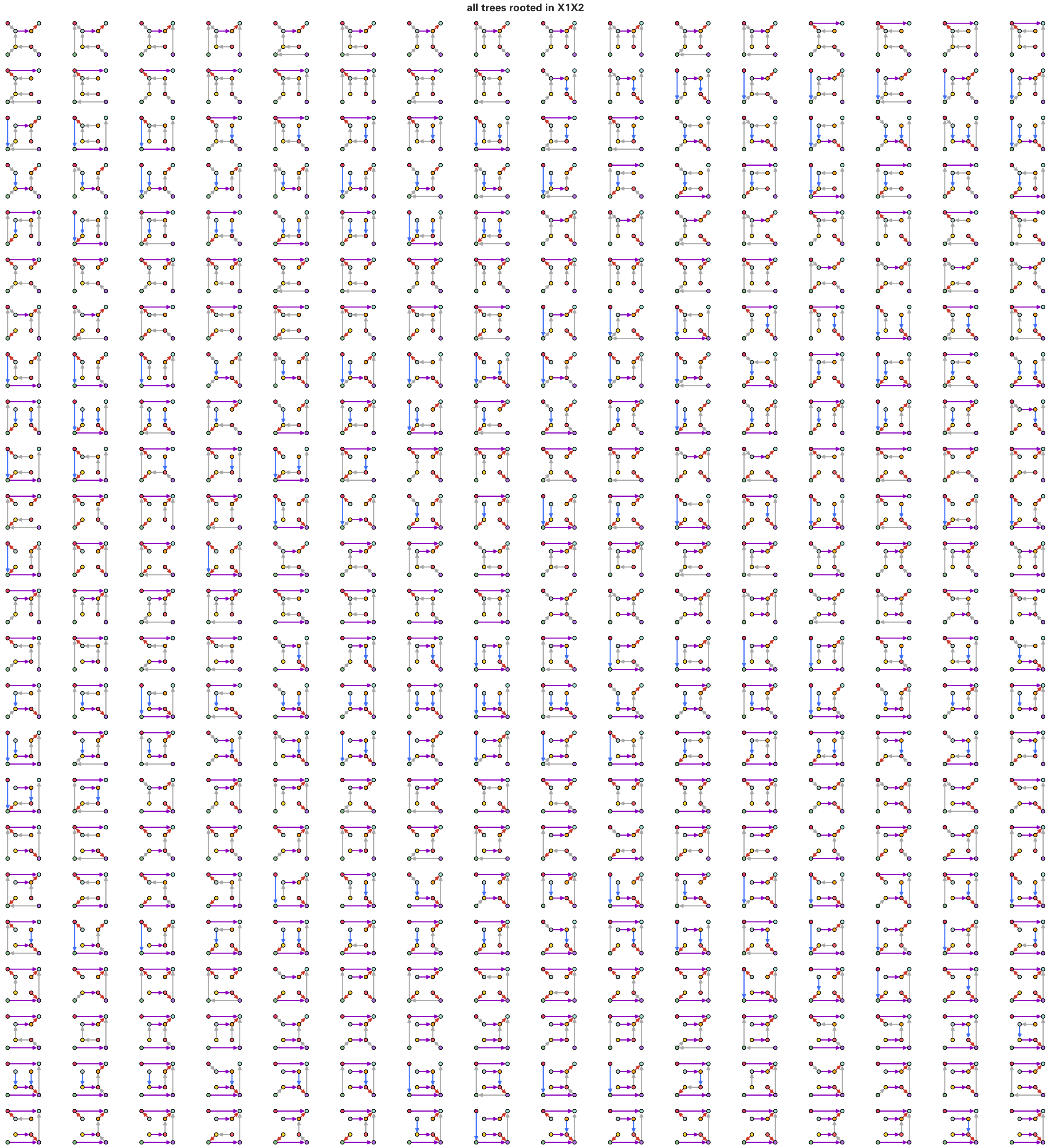

Fig. S38. All directed spanning trees that are rooted in state X_1X_2 for a cubic graph, representing a regulation unit with two transcription factor binding sites. The powers of transcription factors $[X_1]$ and $[X_2]$ found in each spanning tree increases from the top left to the bottom right in this grid. $[X_1]$ and $[X_2]$ are the concentration of the two transcription factors and X_1X2 is the state in which both the transcription factors X_1 and X_2 are bound to the DNA.

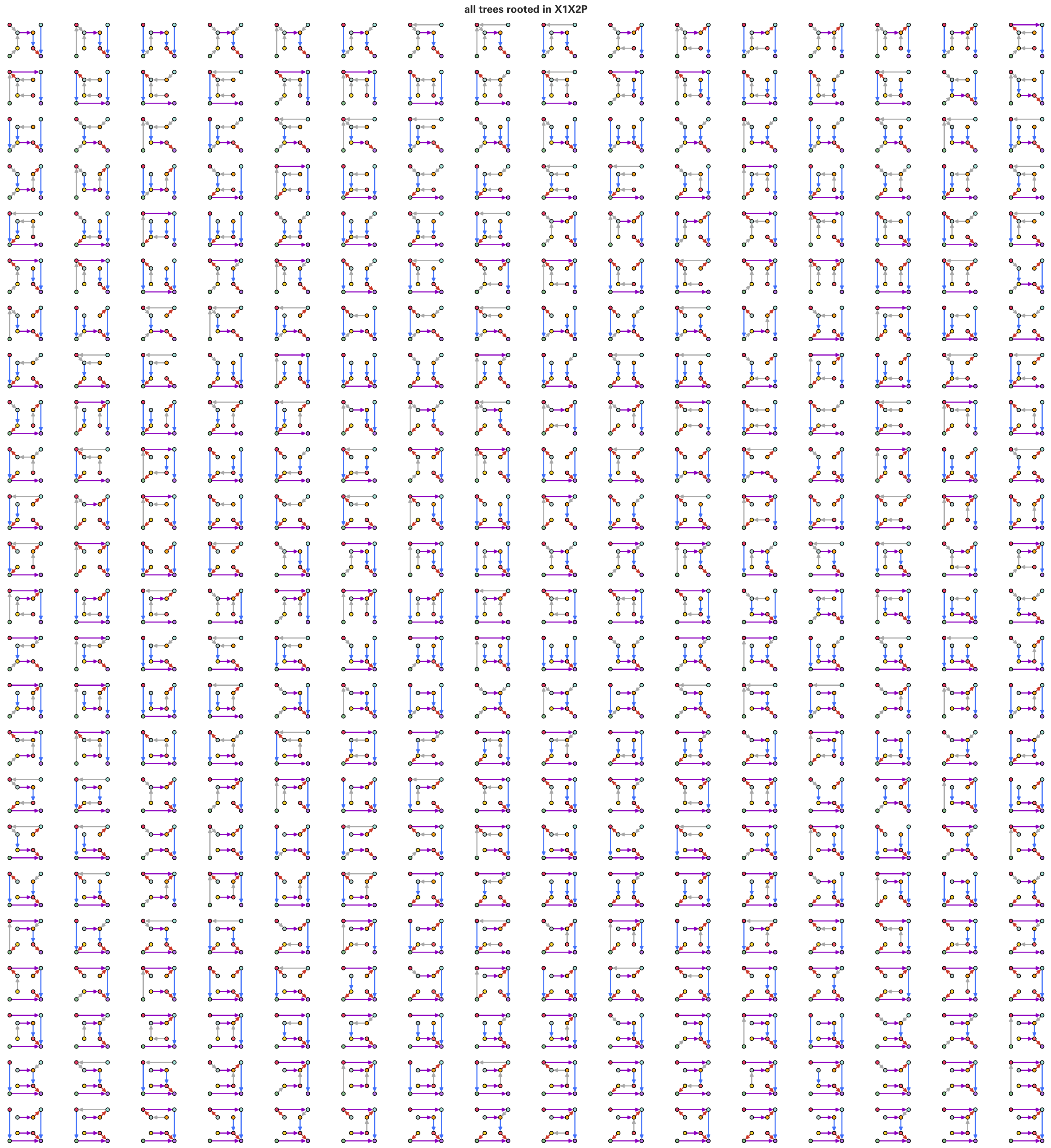

Fig. S39. All directed spanning trees that are rooted in state X_1X_2P for a cubic graph, representing a regulation unit with two transcription factor binding sites. The powers of transcription factors $[X_1]$ and $[X_2]$ found in each spanning tree increases from the top left to the bottom right in this grid. $[X_1]$ and $[X_2]$ are the concentration of the two transcription factors and X_1X_2P is the state in which both of the transcription factors X_1 and X_2, as well as the polymerase P are bound to the DNA.

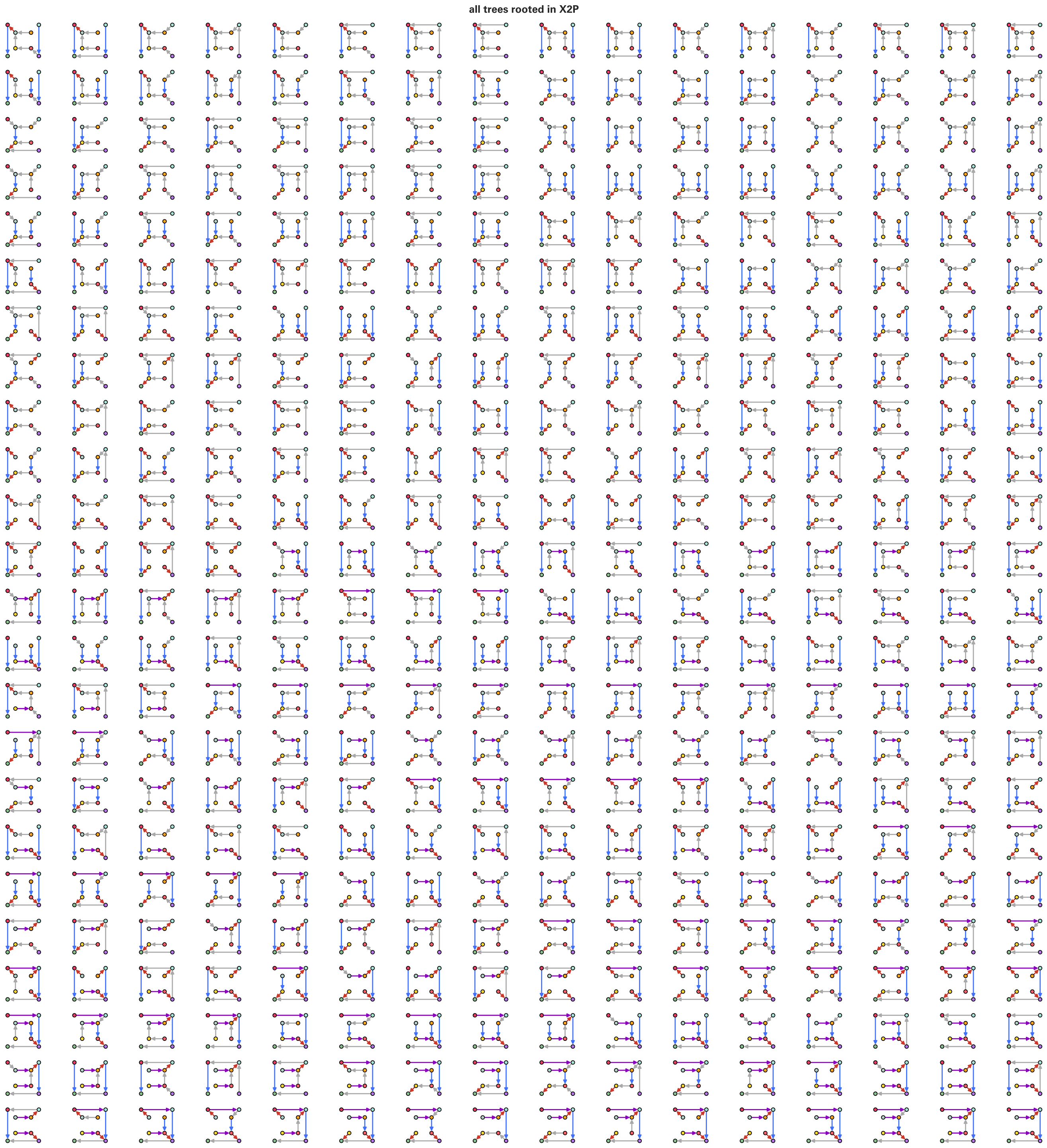

Fig. S40. All directed spanning trees that are rooted in state X_2P for a cubic graph, representing a regulation unit with two transcription factor binding sites. The powers of transcription factors $[X_1]$ and $[X_2]$ found in each spanning tree increases from the top left to the bottom right in this grid. $[X_1]$ and $[X_2]$ are the concentration of the two transcription factors and X_2P is the state in which only the transcription factor X_2 and the polymerase P are bound to the DNA.

C. Common graphs formed by subsets of the hypercube.

C.1. N ***strictly exclusive transcription factors: the book/rolodex graph.*** Often, the binding sites for transcription factors overlap. For instance, four promoters with appreciably-overlapping binding sites in *E. coli* are visualized in Fig. S41. These common overlaps suggest that the presence of transcription factors on a promoter might exclude others from also being bound.

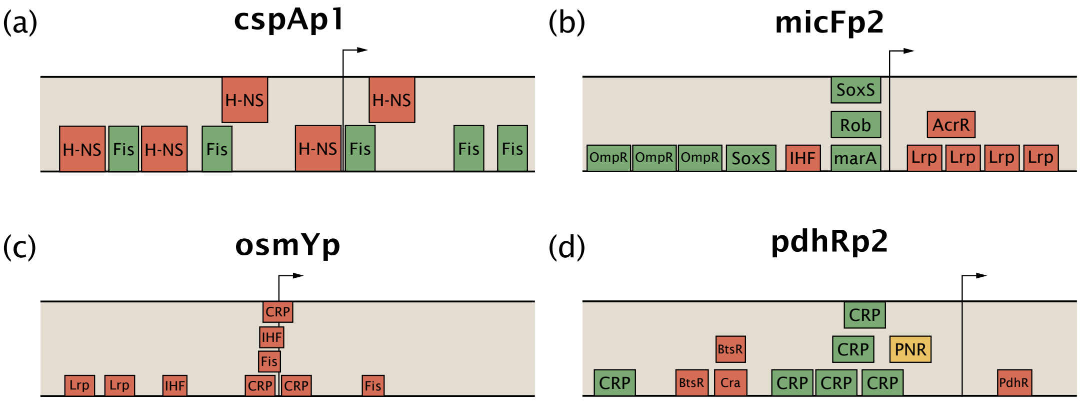

Fig. S41. Four examples showing how *E. coli* promoters regulated by multiple transcription factors often present extensive overlaps between binding sites. (A) Regulation of the promoter cspAp1. (B) Regulation of the promoter micFp2. (C) Regulation of the promoter osmYp. (D) Regulation of the promoter pdhRp2. Binding sites are annotated by their name and colored in green, red, or yellow if the sites are understood to be activating, repressing, or both, respectively. The transcription starting site is indicated by the black arrow. (Note that these schematics reflect the topology/overlaps and relative positions of binding sites, but the size of each binding site's box is not to scale in genomic coordinates.)

If some transcription factors are mutually exclusive with each other and/or the polymerase, the hypercubic graph is pruned to a smaller state space, significantly changing the observed response functional form of the response with respect to the control variables. Consider the highly biologically plausible case where two transcription factors X_1 and X_2 cannot coexist with each other, but each can coexist with the polymerase. The trees that span the resulting graph have particularly simple structure and give correspondingly simple forms for the ultimate response function of the system.

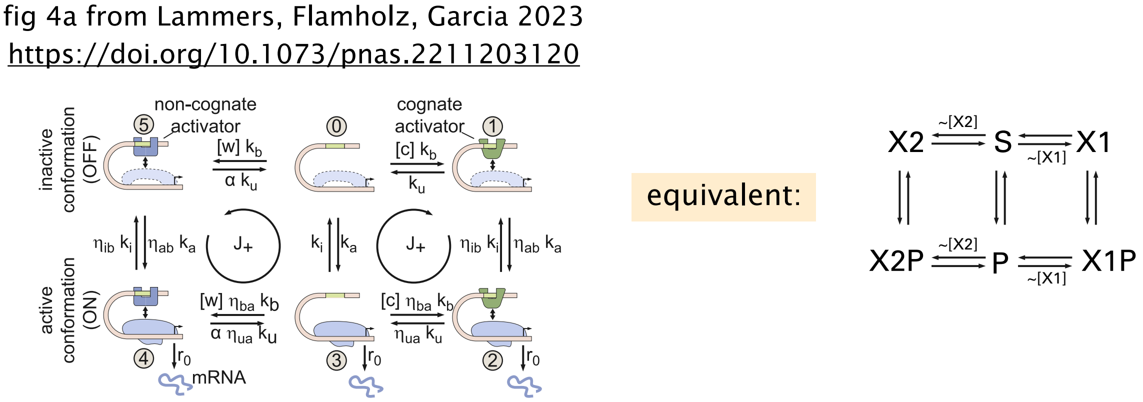

Fig. S42. Regulation unit with two transcription factor, which binding is mutually exclusive, from Reference (28) .When only one transcription factor can bind at a time, but both can bind in the presence of the polymerase, a smaller state space graph is formed. One biological scenario yielding this simple exclusion structure is the setting studied by Reference (28), where both cognate and noncocagnate enhancers modulate the action of polymerase in cellular decision making.

For instance, consider the biological example invoked by Fig 4A of Lammers, Flamholz and Garcia (28), which they use to model interference by noncognate factors in cellular decision making. The state space collapses from a three-dimensional cubic graph (with eight conjoined cycles) to just two conjoined square cycles, as shown in Fig. S42.

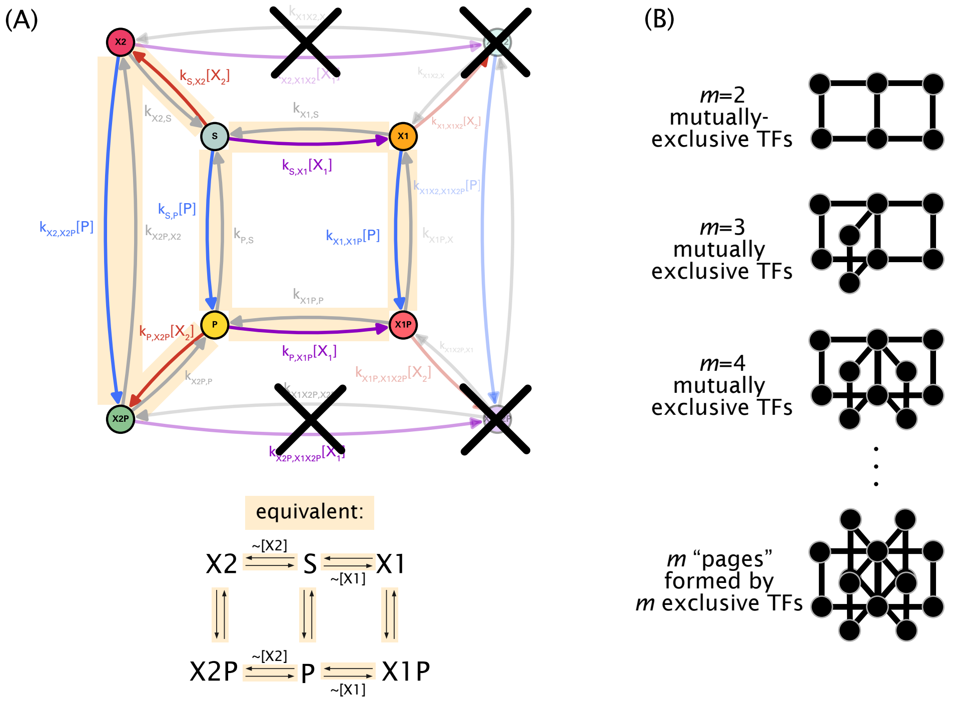

Fig. S43. Relation between the cubic graph and the "book graph" and a generalisation of this "book graph" in m dimensions corresponding to m exclusive transcription factor binding.(A) Schematic localizing the state space (tan outline) of two mutually-exclusive transcription factors inside a full hypercube graph (subject to no exclusions). Forbidden interactions and states are crossed out in black. (B) When multiple transcription factors govern a network and all are mutually exclusive (except with the polymerase), the resulting graph is always a set of m square cycle "pages," each associated with a given transcription factor, conjoined along one central "spine"/edge-pair between the empty S and polymerase-bound P states.

Figure S43 localizes the resulting states of this example as a subgraph of the hypercube graph we just investigated in §B. This graph also achieves analogous structures in higher dimension (with a larger number of transcription factors, still mutually exclusive except for the polymerase): extra "pages" are added around the "axis" of the $S \leftrightarrow P$ transition, creating a structure rather like a cylindrical rolodex. Each of the independent square pages is a cycle between the empty state S, the polymerase-bound state P, plus the states X_j and X_jP where a particular transcription factor X_j is bound alone or bound along with the polymerase, respectively; the exclusions among transcription factors enforce that these cycles are joined only along the central axis. It transpires that some literature refers to this general simple graph structure as a "book graph" (see e.g. Wolfram MathWorld).

Another important, biologically-distinct example where this regulatory architecture manifests is the setting of post-translational modifications, such as phosphorylation, made to a standing pool of transcription factors. For instance, if both unphosphorylated and phosphorylated forms of a transcription factor bind (perhaps with distinct affinity) to the same site on the genome, then these species are effectively mutually-exclusive distinct transcription factors, and will produce a book graph like those visualized in Figure S43B.

C.2. General response function of the book graph from multiple exclusive transcription factors. Having understood that multiple transcription factors form a book graph when they are mutually exclusive, we can deduce the form of any resulting response function. The crucial point is that the states S and P are the only states in the graph which have any outgoing edges dependent on transcription factor concentrations. We conclude that in any spanning tree, at most one outgoing edge can depend on a transcription factor (say X_i) outgoing from S, and at most one edge can depend on a transcription factor (say X_j, possibly with $i = j$) from P. This structural constraint immediately requires that any tree (hence any response function) can only attain exactly the following dependencies on transcription factor concentrations: $\sim [X_i]^2, \sim [X_j]^2$, $\sim [X_i][X_j]$, $\sim [X_i]$, or $\sim [X_j]$, for all pairs of distinct transcription factors (i, j). Accordingly, any response function of a book graph always exhibits the

remarkably simple form[‡],

$$\boxed{\langle r\rangle^{\text{book}} = \frac{a_0 + \sum_{i=1}^{m}(a_{i,1}[X_i] + a_{i,2}[X_i]^2) + \sum_{i\neq j\leq m} b_{ij}[X_i][X_j]}{c_0 + \sum_{i=1}^{m}(c_{i,1}[X_i] + c_{i,2}[X_i]^2) + \sum_{i\neq j\leq m} d_{ij}[X_i][X_j]}}. \qquad [180]$$

Importantly, observe that if only one of the transcription factor concentrations $[X_i]$ is taken to vary (namely, regarding the function above from a hyperplanar slice with all but one transcription factors fixed), then this form collapses all the way down to a ratio of quadratic polynomials in this concentration, exactly the mathematical form studied extensively in the main text.

At equilibrium, detailed balance enforces that every ratio of statistical weights has at most one power of a transcription factor's concentration. This collapse can be understood by the same means as the collapses found for the hypercube, or DNA looping graphs, analyzed earlier: focusing on the possible paths between any pair of states, we see that at most one power of any given transcription factor's concentration $[X_i]$ is accumulated along any simple path between any two states in the graph. Thus any response of a book graph is a linear rational function of transcription factor concentrations at equilibrium, such that Eq. 180 collapses to,

$$\langle r\rangle^{\text{book, eq.}} = \frac{a_0 + \sum_{i=1}^{m} a_i[X_i]}{b_0 + \sum_{i=1}^{m} b_i[X_i]}. \qquad [181]$$

D. All nontrivial, transcriptionally-potent subgraphs of the $n = 4$ hypercube. While we just analyzed the limiting case where all transcription factors are mutually exclusive, more relaxed and complex exclusion topologies (where only some transcription factors or polymerase are incompatible with each other on the genome) are also plausible. In this section, we systematically find the architectures (and associated response functions) of all possible networks with up to three transcription factors. We find these networks by beginning with the tesseract ($n = 3 + 1$ dimensional hypercube) graph and removing all states formed by possible individual, pairwise, triplet, or quadruplet mutual exclusions among the three transcription factors and polymerase. (For instance, the book graph is generated by forbidding states where any pair of transcription factors are present, here specifically excluding states X_1X_2, X_2X_3, and X_1X_3 and their accompanying transitions.) Further, we require that the resulting graphs remain transcriptionally potent (e.g. retain at least one state where polymerase can be bound to the genome) and discard redundant (e.g. literally isomorphic) networks generated during this enumeration. Figs. S44 and S45 summarize the results of this exercise.

This exercise reveals that a large fraction of networks constrained by exclusions show simple mathematical dependencies in at least one of their transcription factors. Specifically, the majority (90 out of the total nontrivial 147 networks) show at most a quadratic dependence in at least one transcription factor; such networks are shaded in purple in Figs. S44 and S45. This quadratic dependence is the same as that analyzed thoroughly in the main text; when the simply-varying transcription factor is the only transcription factor that varies, the resulting response function is subject to all the precise and quantitative mathematical constraints born by the square graph. The result of this exercise is also summarized in Figure 2B of the main text.

[‡] In this work, we have usually absorbed the response function's dependence on polymerase concentrations into coefficients since we regard polymerase as a completely separate (and plausibly much more constrained) biological control variable. However, here it is structurally amusing to notice that the $[X_i]^2$-dependent terms of the response function present all possible powers of $[P]$ from $[P]^0$ (constant) to $[P]^m$. Why? Each tree delivering a quadratic dependence on some transcription factor $\sim [X_i]^2$ cannot accumulate any further dependencies on other transcription factor concentrations, nor can the $S \to P$ edge participate in the tree (since both S and P have "spent" their possible outgoing edges exactly to achieve the $\sim [X_i]^2$ dependence). However, every page of the book graph formed by the transcription factors can easily either include a $[P]$-dependent edge (going from $X_j \to X_jP$) or not (then involving the unbinding edge $X_jP \to P$), and still span the graph's states. In addition, if we relax the requirement that $[X_i]^2$ appear, a separate $[P]^{m+1}$ dependency can be attained by taking all $X_j \to X_jP$ edges with the $S \to P$ (all downward edges) and including up to one $[X_i]$-dependent edge, where the tree is completed by all other radial edges being oriented inwards as unbinding edges, $X_jP \to P$. We propose these structural considerations could be useful for other scenarios where an analog of the book graph appears and the variable P, with which all other transcription factor control variables are compatible, is perhaps instead understood as itself a separate transcription factor.

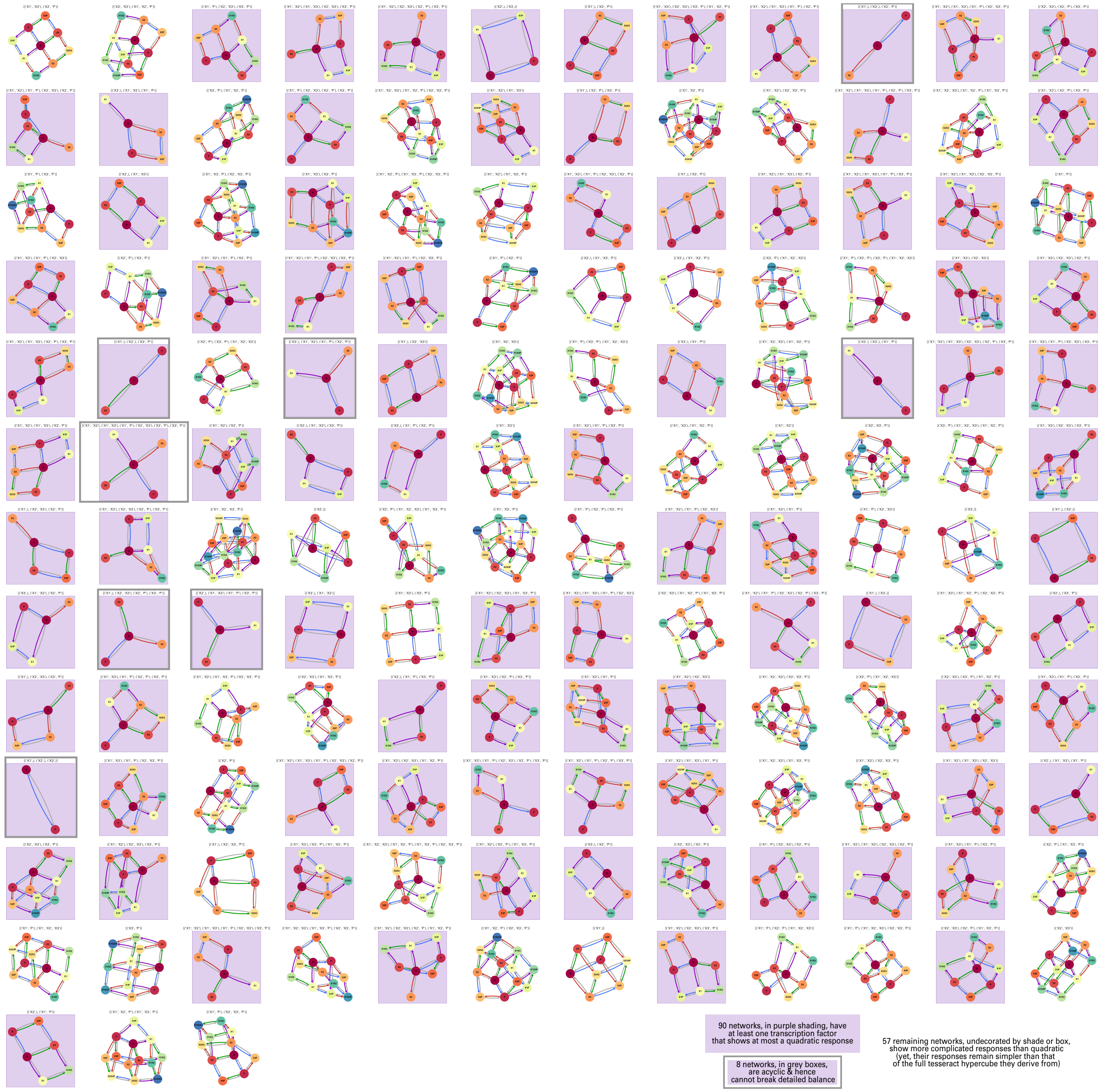

Fig. S44. Comprehensive census of all subsets of the tesseract cube formed by all possible pairwise, triplet, or individual exclusions among the transcription factors and polymerase, restricted to the set where polymerase can still be present. Each panel depicts the state topology of a distinct possible set of mutual exclusions between transcription factors (among themselves) and/or polymerase, as long as the polymerase can be found bound to the genome in at least one state. (The ordering of panels follows an arbitrary enumeration of while maintaining that the polymerase can bind in at least one state.) Purple shading emphasizes those networks that display at least one transcription factor whose response varies as a quadratic response or simpler (namely, matching the mathematical response of a square graph).

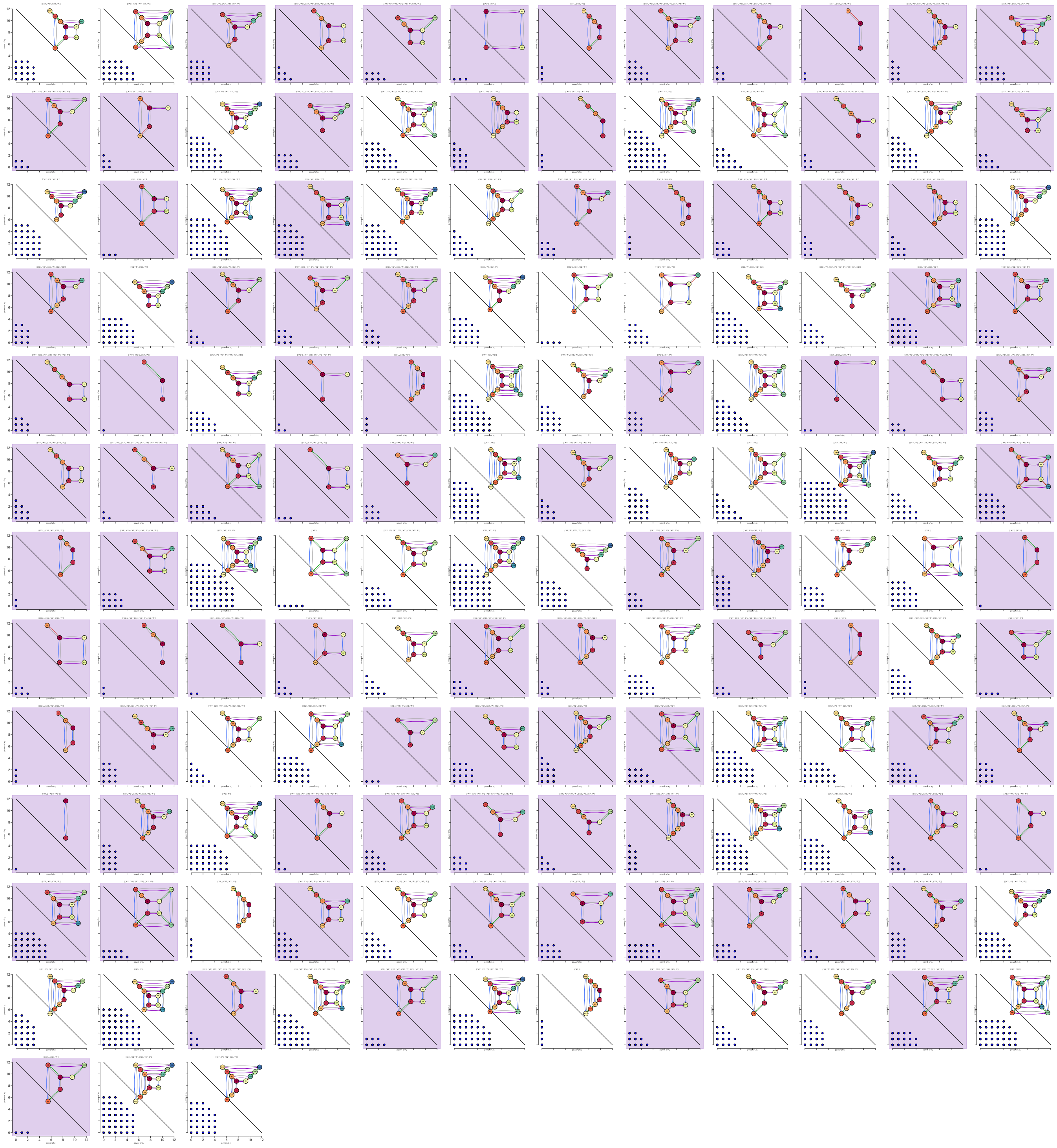

Fig. S45. The same three-transcription-factor-networks identified in Fig. S44, now visualized alongside their attained powers in two of their transcription factor concentrations, $[X_1]$, $[X_2]$. The networks (and their ordering) are the same as those in the earlier Fig. S44; the nodes are just arranged according to a different layout to emphasize their nature as a subgraph of the tesseract graph, whose full set of nodes is shown in Fig. S31B.

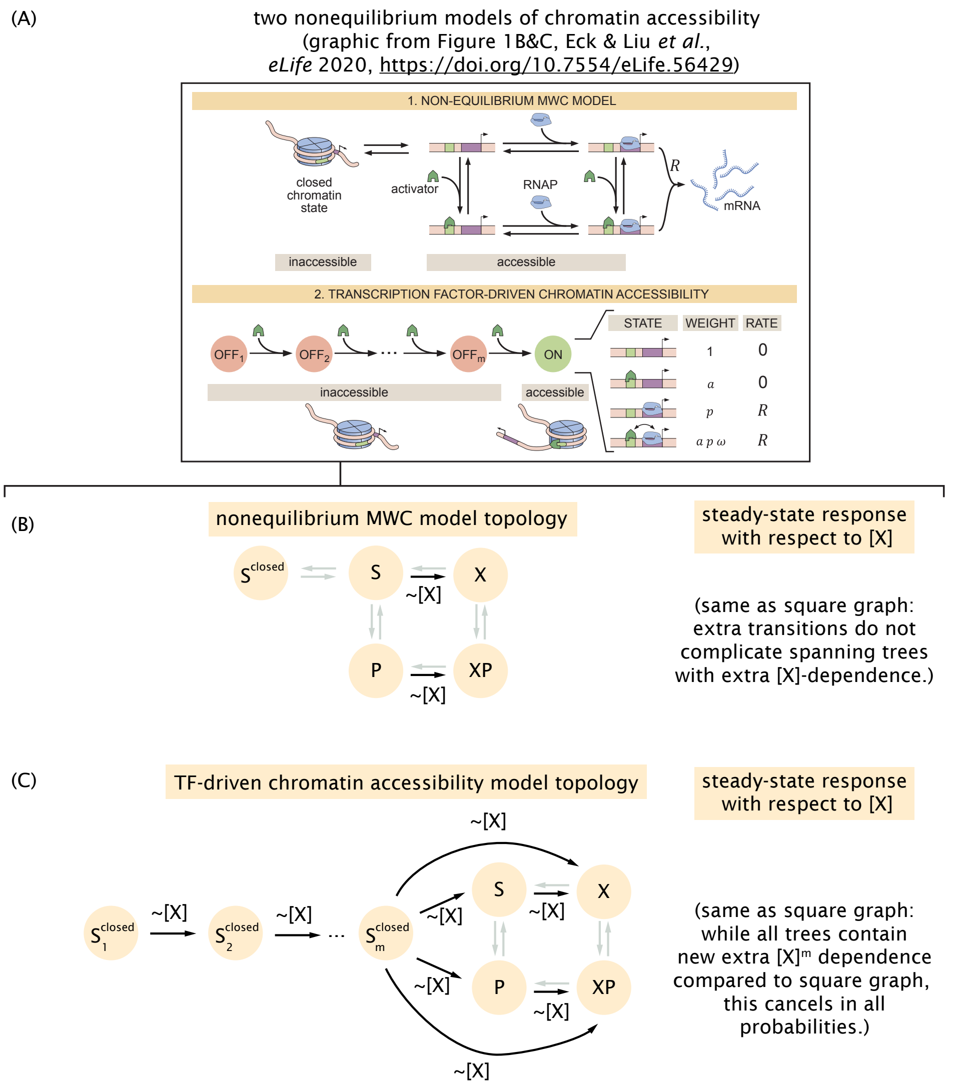

Fig. S46. Successful kinetic models of chromatin accessibility mediated by transcription factors are relateable to the square graph's response. (A) Reference (60) explored how well two contrasting nonequilibrium models—a nonequilibrium MWC model where undriven fluctuations render polymerase binding sites accessible, and a model where transcription factors explicitly and unidirectionally drive egress from a number of inaccessible states—could account for experimental measurements on *hunchback* transcription by the activator Bicoid and the transcription factor Zelda. (B) The nonequilibrium MWC model contains an extra closed state when the genome cannot bound by any proteins; the model also contains two extra accompanying transitions. (B) The second nonequilibrium model, featuring transcription-factor-driven chromatin accessibility, contains m extra states and $m + 4$ extra $[X]$-driven-edges.

E. Connecting to successful nonequilibrium dynamic models of transcription-factor-driven chromatin accessibility. In Reference (60), Eck and Liu and coworkers contrast how two nonequilibrium models can describe how transcription factors activate chromatin accessibility and ultimately transcription. Here we discuss how despite the highly distinct biological details of the eukaryotic transcriptional dynamics in such models, both scenarios inherit the exact quantitative behavior of the square graph while modeling the steady-state mRNA production rate with respect to a transcription factor.

Figure S46A illustrates the models proposed by Reference (60). In the first model, which shows the graph topology of Fig. S46B, a single extra state (representing the genome wrapped inaccessibly around chromatin) is added to the square graph; this state carries just two additional transitions, both to the empty genome state S. Every spanning tree on this graph can be represented as a spanning tree on the square graph times one of these additional edges. Since Reference (60) proposes that these transitions as thermally-activated, and not explicitly controlled by the transcription factor concentration, the resulting spanning trees inherit exactly the same dependence on transcription factor concentrations as in the square graph. So, any steady-state response observable also exactly mathematically evokes the square graph's response.

In contrast, the second model—illustrated in Fig. S46C—proposes instead that transcription factors explicitly drive the system to exit some number m of closed-chromatin states where the genome is unavailable to bind to the polymerase. This introduces many (specifically, $m+4$) new edges that depend on the transcription factor concentration $[X]$ relative to the square graph, which might superficially suggest a different algebraic dependence of observables in this graph. However, these additional edges are modeled as irreversible transitions—with considerable quantitative success while matching dynamic measurements, as reported in (60)'s analysis. Accordingly, every tree T_{large} on this larger network can be factored as a product of rates along the

 S. D. Mahdavi*, G. L. Salmon*, P. Daghlian, H. G. Garcia, and R. Phillips (* contributed equally)

irreversible path from the first OFF state to the last OFF state, times a spanning tree on the square graph. Since *all* trees on this network inherit this common irreversible factor, all *probabilities* (and hence all response observables) enjoy a cancellation of this common factor. In sum, despite the additional complexity of transitions, we directly conclude that all steady-state outputs from this transcription-factor-mediated chromatin accessibility out of equilibrium still manifest exactly the response of the square graph.

Ultimately, Reference (60) advocates for this second model; they establish that this model is deeply consistent with a battery of precision measurements on transcriptional *dynamics*. We note that while our analysis on response observables focuses on steady-state probabilities, not explicit transient dynamics *per se*, it is not difficult to establish that the dynamic behavior of mRNA accumulating according to this second model is also dominated by the a polynomial in the transcription factor concentration $[X]$ and time that exponentially decays to the square-graph's steady-state. This behavior follows from the statement that

$$\frac{d\text{mRNA}}{dt} = r\underbrace{\left(p_X([X] + p_{XP}([X])\right)}_{\sim\text{square graph}} p_m([X], t), \qquad [182]$$

where $p_m([X], t)$ represents the probability of being in the last OFF-state at time t, and r is the rate at which transcription proceeds when the polymerase is in the ON state. When the system begins in the first, most-removed OFF state, the latter probability $p_m(t)$ can be shown to increase essentially exponentially in time. This behavior also emphasizes the role played by the square graph even in dynamics. Such straightforward connections beckon to be interrogated further in future work.

F. Analysis of larger regulatory networks involving DNA looping. Next, we study transcriptional regulation when DNA looping is possible. Looping can induce repression (61) or activation (62) of a gene; these modalities are achieved using very different regulatory network topologies.

F.1. Repression. We consider the case of the Lac repressor. The repressor forms stable dimers, which can still bind to the DNA and repress (63). Near the promoter binding site, there is a main and auxiliary operator (36). When the repressor binds to the main operator, the gene is effectively repressed. The three states of simple repression depicted in Figure S4 are still present: empty DNA, DNA with the polymerase being bound, and DNA with the repressor being bound. When Lac repressor binds to the auxiliary site, there is no effective repression because it doesn't prevent the polymerase from binding. Three extra states are added: two Lac repressor dimers binding to both main and auxiliary operators, one dimer binding to the auxiliary operators with or without bound polymerase (64). DNA looping enables the concurrent binding of the Lac repressor to both the main and auxiliary operators. Looping is believed to need some energy expenditure because of the bending of the DNA, but once the DNA is looped the energy of bending is compensated by the stabilization of the dimer the repression is stronger. We represent this regulation unit in the graph represented in Figure S47A.

To describe new regulatory architectures, with the ability for the DNA to loop, we write down new graphs represented in Figures S47 and S50. In this new setting, we want to do a similar systematic study than in the main text for the 4 state cycle, when we break equilibrium by adding energy along each edge, and reporting the evolution of the output curves. In the four state cycles, we used the notion of drive, characterised by a chemical potential $\Delta\mu$ to measure how we are from equilibrium Eq. [130]. We recall that in this setting $\Delta\mu = k_B T \ln\gamma$, with γ the ratio of the product of the clockwise rates and the product of the counter clockwise rates of the cycle. We saw that this notion of drive is generalisable for any cyclic network in the section D. Nevertheless, we notice, that there are more than one cycle in the networks enabling looping. Therefore, we need a new definition for the drive. We first compute the entropy dissipation rate $\dot{W} = \frac{dS}{dt}$ in the repression by looping graph, starting from the following formula (65):

$$\frac{\dot{W}}{k_B T} = \sum_{i<j} \Delta J_{ij} \ln \frac{k_{ij} p_i}{k_{ji} p_j}, \qquad [183]$$

with $\Delta J_{ij} = k_{ij} p_i - k_{ji} p_j$.

At steady state, we can write the following equations to express the conservation of fluxes :

$$\begin{cases} \Delta J_{SP} = \Delta J_{PXP} = \Delta J_{XPX} = \Delta J_1 \\ \Delta J_{RXR} = \Delta J_{XRX} = \Delta J_2 \\ \Delta J_{XL} = \Delta J_{LR} = \Delta J_3 \\ \Delta J_{XS} = \Delta J_1 - \Delta J_2 - \Delta J_3 \\ \Delta J_{RS} = \Delta J_2 + \Delta J_3. \end{cases} \qquad [184]$$

We then use these equations in the expression of the entropy rate to write :

$$\begin{aligned} \frac{\dot{W}}{k_2 T} &= J_1 \ln \frac{k_{SP} k_{PXP} k_{XPX}}{k_{PS} k_{XPP} k_{XXP}} \frac{p_S}{p_X} + J_2 \ln \frac{k_{XRX} k_{RXR}}{k_{RXX} k_{RRX}} \frac{p_X}{p_R} + J_3 \ln \frac{k_{XL} k_{LR}}{k_{LX} k_{RL}} \frac{p_X}{p_R} + (\Delta J_1 - \Delta J_2 - \Delta J_3) \ln \frac{k_{XS} p_X}{k_{SX} p_S} + (\Delta J_2 + \Delta J_3) \ln \frac{k_{RS} p_R}{k_{SR} p_S} \\ &= J_1 \ln\gamma_1 + J_2 \ln\gamma_2 + J_c \ln\gamma_3, \end{aligned} \qquad [185]$$

with $\gamma_1 = \frac{k_{SP} k_{PXP} k_{XPX} k_{XS}}{k_{PS} k_{XPP} k_{XXP} k_{SX}}$, $\gamma_2 = \frac{k_{RS} k_{SX} k_{XRX} k_{RXR}}{k_{SR} k_{XS} k_{RXX} k_{RRX}}$ and $\gamma_3 = \frac{k_{RS} k_{SX} k_{XL} k_{LR}}{k_{SR} k_{XS} k_{LX} k_{RL}}$.

So the entropy rate can be decomposed into three driving potentials $\Delta\mu_1 = \ln\gamma_1$, $\Delta\mu_2 = \ln\gamma_2$, $\Delta\mu_3 = \ln\gamma_3$ and fluxes $\Delta J_1, \Delta J_2, \Delta J_3$ of 3 independent cycles, with the following expression :

$$\frac{\dot{W}}{k_B T} = \Delta J_1 \Delta\mu_1 + \Delta J_2 \Delta\mu_2 + \Delta J_3 \Delta\mu_3. \qquad [186]$$

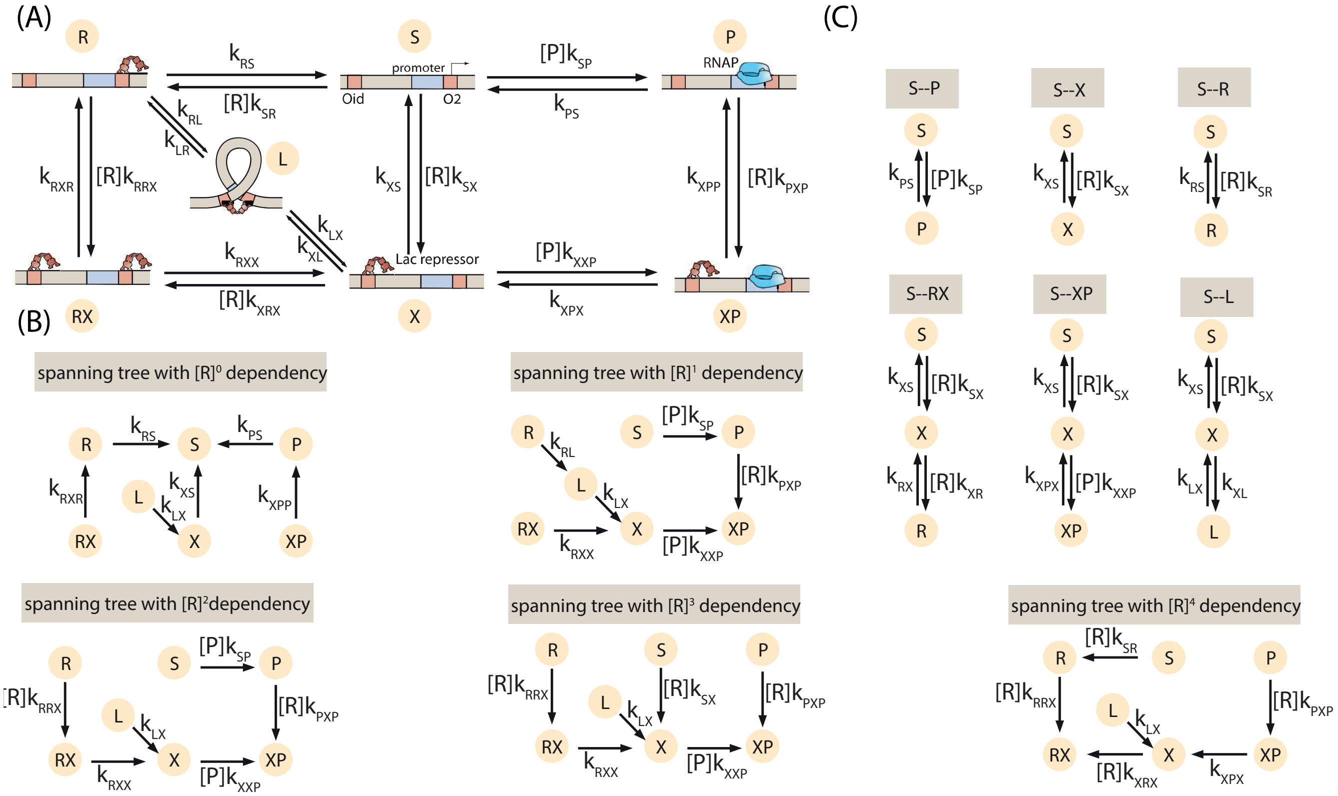

Fig. S47. Graph representing a repression motif with looping of the DNA and characteristic paths and spanning trees explaining the functional form of the output function. (A) The different states and transitions between them for a regulatory unit performing repression and looping. On each arrow is written the rate at which this transition occurs. When a molecule (here RNA polymerase P or repressor R) binds, the rate is proportional to the concentration in this molecule. The state S represents the empty DNA, the state P, the DNA with only the RNAP bound, X the DNA with only the repressor bound to the auxiliary (non-repressing) site, R the repressor bound to the main (repressing). XP is the state in which both the polymerase is bound to the DNA, as well as the repressor on the auxiliary site, and RX is the state in which two repressors are bound on the two operons. Finally, L is the looped state in which a single repressor dimer is bound to both operons. (B) Weighted spanning trees having no, linear, quadratic, cubic, or quartic dependency on $[R]$.(C) Paths from the state S to any other state of the graph of (A).

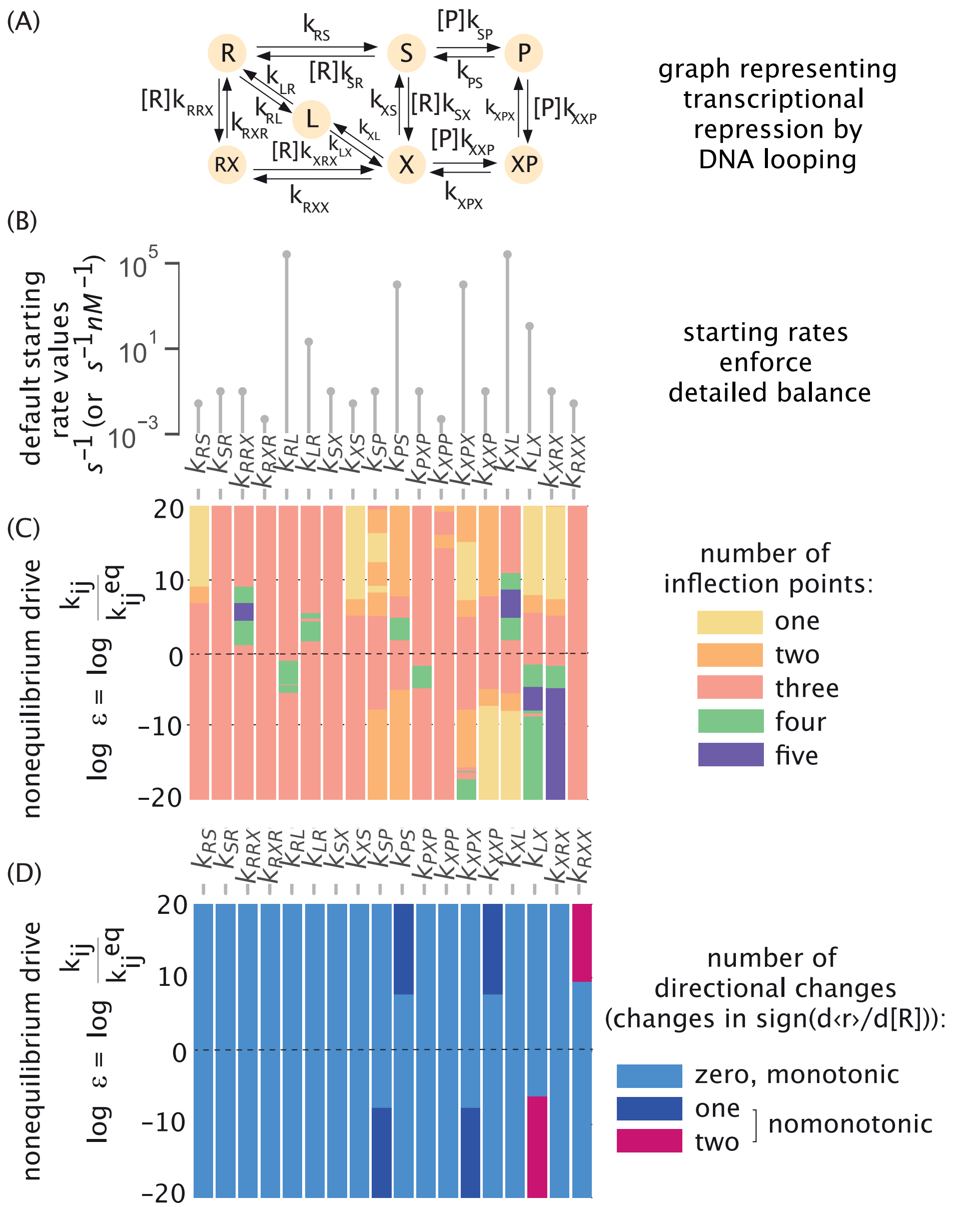

Fig. S48. Incorporating energy along each edge of the repression by looping graph. (A) Graph representing a regulation unit, with two repressor binding sites and the possibility for the DNA to loop. This is a schematized version of the Figure S47(A). On each arrow is written the rate at which this transition occurs. When a molecule (here RNA polymerase P or repressor R) binds, the rate is proportional to the concentration in this molecule. The state S represents the empty DNA, the state P, the DNA with only the RNAP bound, X the DNA with only the repressor bound to the auxiliary (non-repressing) site, R the repressor bound to the main (repressing). XP is the state in which both the polymerase is bound to the DNA, as well as the repressor on the auxiliary site, and RX is the state in which two repressors are bound on the two operons. Finally, L is the looped state in which a single repressor dimer is bound to both operons. (B) Default starting equilibrium rates. (C) Number of inflections, with respect to the concentration of repressor in a logarithmic scale, of the output curve $\langle r \rangle$, which can go from 1 to 5. (D) Number of zeros of the first derivative, with respect to the concentration of repressor in a logarithmic scale, of this rate, which can go from 0 to 2. Note that $\epsilon_{ij} = \frac{k_{ij}}{k_{ij}^{eq}}$.

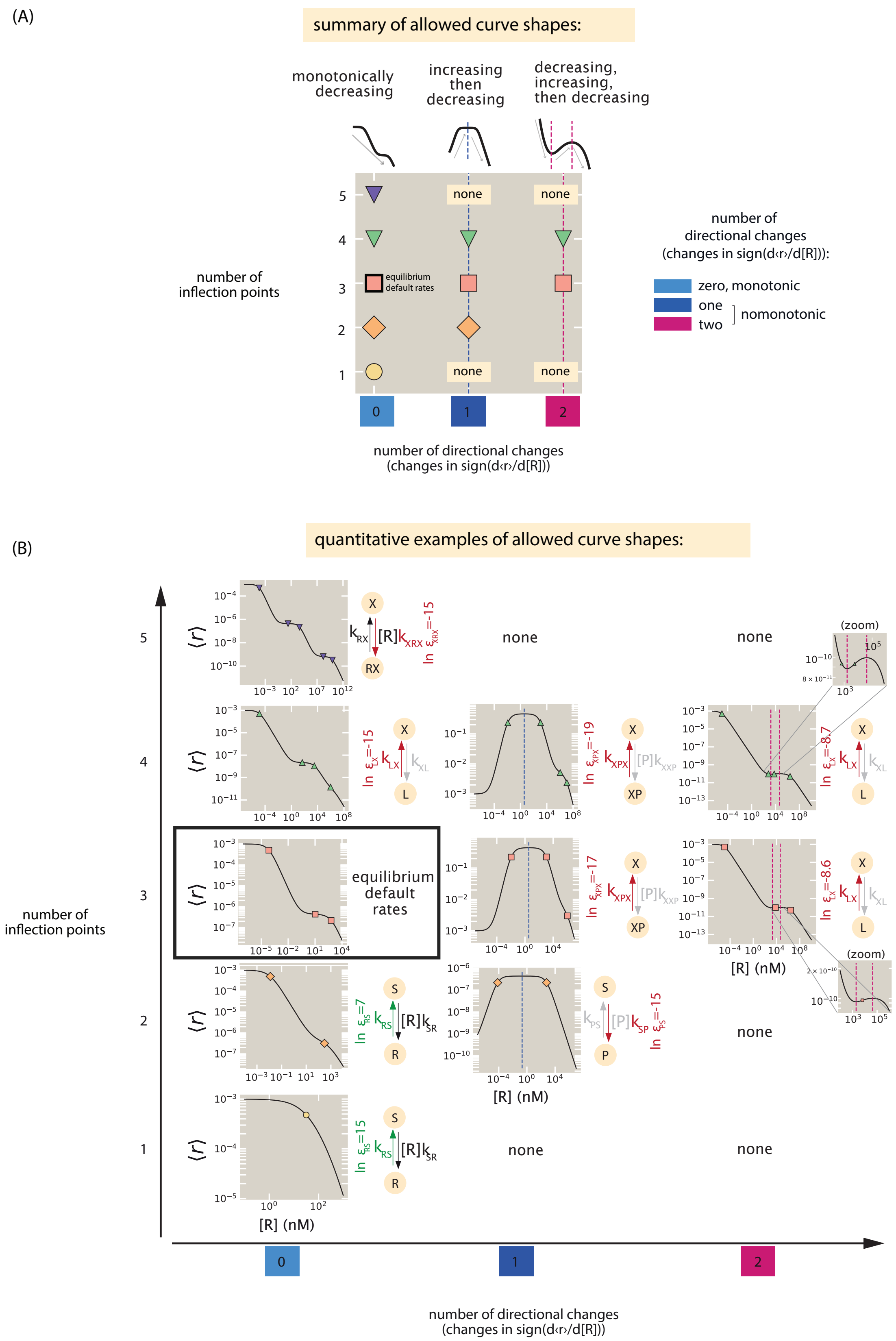

Fig. S49. Different shapes of output curves reachable when driving one single edge and departing from equilibrium at the default rates represented in Figure S48(B). (A) Reachable number of inflection points in a logarithmic scale and change of sign of the derivative or $\langle r \rangle([R])$. When a symbol is plotted at the location (i, j), a curve with i changes of sign of the first derivative and j inflections can be reached. The bold symbol in the phenotype of the curve at equilibrium for the default starting rate values. (B) Examples of curves for these phenotypes, specifying the amount of drive injected in the system and which edged is pushed. The boxed plot is the one at equilibrium.

To find the functional form of the output out of equilibrium, we find one spanning with every possible order of $[R]$ dependency, and illustrate the result in Figure S47(B). This results in the following output function :

$$\langle r \rangle = \frac{\sum_{i=0}^{4} a_i [R]^i}{\sum_{i=0}^{4} b_i [R]^i}. \quad [187]$$

Let's first consider this function at equilibrium and see how it reduces to a simpler function. At equilibrium, we can use the result stated in Eq. [46]. We chose the reference state i_0 to be S, we consider all the paths from S to all the other nodes of the network. As the binding events are bi-molecular events, any of these paths has at most dependency $[R]^2$ (for state S to state RX, where two repressors are bound, involving two bi-molecular steps) and at least $[R]^0 = 1$. This is illustrated in Figure S47(A). The resulting output form at equilibrium is therefore :

$$\langle r \rangle_{eq} = \frac{a_0^{eq} + a_1^{eq}[R] + a_2^{eq}[R]^2}{b_0^{eq} + b_1^{eq}[R] + b_2^{eq}[R]^2}. \quad [188]$$

So at equilibrium, the output function is a ratio of polynomials of order 2. For the rest of the analysis we settle to $\langle r \rangle = p_P + p_{XP}$, the probability for the polymerase to be bound to the DNA, proportional to the mRNA production rate. This observable has the feature of having respectively a polynomial of order 3 in the concentration $[R]$ in the numerator, and of order 4 in the denominator. Therefore, the limit at an infinite concentration of repressor is zero. The system always represses at high concentrations. Let's first consider the system at equilibrium. For this, we compute the equilibrium rates, based on measurements found in the literature. We proceed to a couple of simplifications. We take all the on rates of the system to be equal to k_{on}, the diffusion-limited values. We take $k_{on} = 0.1 s^{-1} nM^{-1}$, based on the computation of diffusion limited rates of Section B. The off-rates are then computed with the equilibrium constants, which have been measured for diverse repressors and promoters. We report three useful dissociation constants: K_{RNAP} the dissociation constant of polymerase to the DNA, K_{O_1}, and $K_{O_{id}}$. The two latter are respectively the dissociation constant of the LacI tetramer to the main operator O_1 and to the auxiliary operator O_{id} (66). We can compute all the rates with these parameters except the rates involving looped states. The looping rates k_{XL} and k_{RL} are taken to be equal because of the symmetry of this looping state with respect to the principal or auxiliary operator. We take this rate to be limited by the activation energy of looping, following the Eyring equation. : $k_{XL} = \frac{\kappa k_B T}{h} e^{-\beta \Delta G^\dagger}$, with k_B the Boltzmann constant, T the temperature, h the Planck constant, $\beta = \frac{1}{k_B T}$, $\Delta G^\dagger$ the Gibbs energy of activation κ is the transmission coefficient. The transmission coefficient κ is frequently postulated to be unity, as it represents the proportion of flux traversing the transition state which advances to the product without any retrograde passage through the transition state. Consequently, an ascribed value of unity to the transmission coefficient denotes the ideal fulfillment of the fundamental assumption in transition state theory, wherein the absence of recrossing is assumed. The Gibbs energy takes into account binding/unbinding energy barriers as well as entropic barriers. We will assimilate it to the modeled free energy activation barrier of looping found in the literature (67). For the unlooping rates. We use the notion of dissociation equilibrium constant and their expression based on the difference of energy between two stable states as stated in Eq. [61]. Therefore, we write $k_{LR} = k_{RL} K_{O_{id}} \frac{e^{\beta \Delta F_{loop}}}{N_{ns}}$, with N_{ns} the number of non specific sites. The free of looping is also taken from the literature (67).

To conclude, we just need to have access to a few parameters, written in the equation below.

$$\begin{cases} [P] & = 100 nM \\ K_{O_1} & = 0.27 nM \\ K_{O_{id}} & = 0.05 nM \\ K_{RNAP} & = 10^5 nM \\ N_n s & = 5 \cdot 10^6 \\ \beta \Delta G^\dagger & = 17 \\ \beta \Delta F_{loop} & = 9 \end{cases} \quad [189]$$

We can then compute all the rates as follows.

$$
\begin{cases}
k^{eq}_{SR} &= k_{on} = 0.1s^{-1}nM^{-1} \\
k^{eq}_{RS} &= K_{O_1}k^{eq}_{SR} = 2.7 \cdot 10^{-2}s^{-1} \\
k^{eq}_{RRX} &= k_{on} = 0.1s^{-1}nM^{-1} \\
k^{eq}_{RXR} &= K_{O_{id}}k^{eq}_{RRX} = 5 \cdot 10^{-3}s^{-1} \\
k^{eq}_{RL} &= k_0 e^{\Delta G^\dagger} = 2.6 \cdot 10^{5}s^{-1} \\
k^{eq}_{LR} &= k^{eq}_{RL}K_{O_{id}}\frac{e^{\beta \Delta F_{loop}}}{N_{ns}} = 2.1 \cdot 10^{1}s^{-1} \\
k^{eq}_{SX} &= k_{on} = 0.1s^{-1}nM^{-1} \\
k^{eq}_{XS} &= k^{eq}_{SX}K_{O_1} = 2.7 \cdot 10^{-2}s^{-1} \\
k^{eq}_{SP} &= k_{on} = 0.1s^{-1}nM^{-1} \\
k^{eq}_{PS} &= K_{RNAP}k^{eq}_{SP} = 1.0 \cdot 10^{4}s^{-1} \\
k^{eq}_{PXP} &= k_{on} = 0.1s^{-1}nM^{-1} \\
k^{eq}_{XPP} &= K_{O_{id}}k^{eq}_{PXP} = 5.0 \cdot 10^{-3}s^{-1} \\
k^{eq}_{XXP} &= k_{on} = 0.1s^{-1}nM^{-1} \\
k^{eq}_{XPX} &= K_{RNAP}k^{eq}_{XXP} = 1.0 \cdot 10^{4}s^{-1} \\
k^{eq}_{XL} &= k_0 e^{\Delta G^\dagger} = 2.6 \cdot 10^{5}s^{-1} \\
k^{eq}_{LX} &= k^{eq}_{XL}K_{O_1}\frac{e^{\beta \Delta F_{loop}}}{N_{ns}} = 1.1 \cdot 10^{2}s^{-1} \\
k^{eq}_{XRX} &= k_{on} = 0.1s^{-1}nM^{-1} \\
k^{eq}_{RXX} &= k^{eq}_{XRX}K_{O_1} = 2.7 \cdot 10^{-2}s^{-1}
\end{cases}
\qquad [190]
$$

The curve with the rates valued at default equilibrium values is depicted in Figure S48(B). The equilibrium curve is plotted in Figure S48. It is monotonic and has three inflections, this phenotype is reachable at equilibrium because of the stated form at equilibrium Eq. [188]. When the concentration of repressor R increases, the probability for the polymerase to be bound decreases until vanishing because the system is in state RX with probability 1. This more complex functional dependence on $[R]$ can result in new phenotypes. In a systematic study, we increase or decrease a rate along each edge of the graph. Through this operation, we incorporate energy in the system along this edge, starting from equilibrium. Indeed for a single edge push, we can always write the dissipation rate as follows: $\dot{W}_{\epsilon_{ij}} = k_B T \Delta J_{ij} \ln \epsilon_{ij}$, with ΔJ_{ij} the flux along the edge (i, j). From Eq. [186], we can see that there is a simple connection between the magnitude of the edge pushed away from equilibrium and the energy dissipation: $|\Delta\mu| = |\ln \epsilon|$, with ϵ the ratio between the equilibrium rate and the new value of the rate out of equilibrium. We dissect the different resulting curve shapes. In Figure S48(C), we notice that the number of inflection points that can be reached is up to 5, in comparison to up to 3 for a network with 1 transcription factor. This means that the slope of the function reaches more extrema, and therefore abrupt changes of sensitivity, at different values of the concentration of the transcription factor. Therefore, at different values of concentration, we can have significantly different values of mRNA production but also of sensitivity, enabling different behavior of the regulation motif when the copy number of the repressor varies.

We stated that for a square graph, representing the binding of one transcription factor, the output curve can be nonmonotonic, enabling the regulation unit to behave like an activator or a repressor depending on the concentration of the transcription factor. For this architecture, the output function conserves this reachable feature. In fact, the sign of the first derivative can change up to 2 times. as we see in Figure S48(D). This means that the regulatory identity can change up to two times. As the function is decreasing to 0 at infinite concentration, we know that output curves with one change of sign in its first derivative are first activating and then repressing as depicted in the pictogram of Figure S48(E). Similarly, for two changes of signs of the first derivative, the function is first decreasing then increasing, before decreasing again. The total number of phenotypes reachable with energy expenditure along one edge, departing from the equilibrium rate constants S48(B), is now broadened, as pictured in Figure S48. Nevertheless, it is interesting to note, that some phenotypes could be reached at equilibrium: from our analysis in the main text of a 4 states regulation motif with one transcription factor, we know that an output having the shape of a ratio of polynomials of order 2, can have either one change in sign of the first derivative and two inflection points, or be monotonic and have one or three inflection points. So in full generality 3 phenotypes of Figure S48(F) are reachable at equilibrium. Nevertheless, as we settle to the case of repression by looping the system has also constraints on the values of the rates and not just on the architecture of the network. This is why equilibrium in this setting is less general.

F.2. Repression by DNA looping is subject to kinetic criteria constraining shapes. Now, we conduct a short analysis on the accessibility of non-monotonicty for the output curve at equilibrium. As claimed in Eq. [188], any output curve, as function of the concentration of transcription factor [R], of a graph with an architecture depicted in Figure S47(A) is a ratio of polynomial or order maximum 2, over a polynomial of order two. We saw previously, that such algebraic expression could be non monotonic under certain conditions. To derive this condition, based on rate constants, we need to express the different coefficients of Eq. [188]. If we consider the output function to be the mRNA production rate, we have to consider an observable proportional to the probability of the polymerase being bound to the DNA, written in Eq. [191], with ρ_Y the statisical weight of a state Y.

$$
\langle r \rangle = \frac{\rho_P + \rho_{XP}}{\rho_P + \rho_{XP} + \rho_S + \rho_X + \rho_R + \rho_L + \rho_{RX}} \qquad [191]
$$

As done previously, we rearrange this expression to make detailed balance ratios appear. This is written in equation Eq. [192]

$$\langle r \rangle = \frac{1 + \frac{\rho_{XP}}{\rho_P}}{1 + \frac{\rho_{XP}}{\rho_P} + \frac{\rho_S}{\rho_P} + \frac{\rho_X}{\rho_S}\frac{\rho_S}{\rho_P} + \frac{\rho_R}{\rho_S}\frac{\rho_S}{\rho_P} + \frac{\rho_L}{\rho_X}\frac{\rho_X}{\rho_S}\frac{\rho_S}{\rho_P} + \frac{\rho_{RX}}{\rho_X}\frac{\rho_X}{\rho_S}\frac{\rho_S}{\rho_P}} \qquad [192]$$

We apply detailed balance along every neighboring pair of edges needed, written hereafter in Eq. [193].

$$\begin{cases} \rho_L k_{LX} = \rho_X k_{XL} \\ \rho_{XP} k_{XPP} = \rho_P k_{PXP} \\ \rho_S k_{SP} = \rho_P k_{PS} \\ \rho_X k_{XS} = \rho_S k_{SX}[R] \\ \rho_R k_{RS} = \rho_S k_{SR}[R] \\ \rho_{RX} k_{RXX} = \rho_{XP} k_{XRX}[R] \end{cases} \qquad [193]$$

This gives us the following expression at equilibrium.

$$\langle r \rangle = \frac{1 + \frac{k_{PXP}}{k_{XPP}}[R]}{1 + \frac{k_{PS}}{k_{SP}} + \left(\frac{k_{PXP}}{k_{XPP}} + \frac{k_{SX}k_{PS}}{k_{XS}k_{SP}} + \frac{k_{SR}k_{PS}}{k_{RS}k_{SP}} + \frac{k_{XL}k_{SX}k_{PS}}{k_{LX}k_{XS}k_{SP}}\right)[R] + \frac{k_{SX}k_{PS}k_{XRX}}{k_{XS}k_{SP}k_{RXX}}[R]^2} \qquad [194]$$

We notice that for the mRNA production the coefficient a_2^{eq} vanishes, in other terms, the output function is a polynomial of order one, over a polynomial of order two. We also write the expression of the other coefficients hereafter.

$$\begin{cases} a_0^{eq} & = 1 \\ a_1^{eq} & = \frac{k_{PXP}}{k_{XPP}} \\ b_0^{eq} & = 1 + \frac{k_{PS}}{k_{SP}} \\ b_1^{eq} & = \frac{k_{PXP}}{k_{XPP}} + \frac{k_{SX}k_{PS}}{k_{XS}k_{SP}} + \frac{k_{SR}k_{PS}}{k_{RS}k_{SP}} + \frac{k_{XL}k_{SX}k_{PS}}{k_{LX}k_{XS}k_{SP}} \\ b_2^{eq} & = \frac{k_{SX}k_{PS}k_{XRX}}{k_{XS}k_{SP}k_{RXX}} \end{cases} \qquad [195]$$

We keep the notations for a_1^{eq}, b_0^{eq}, b_1^{eq}, b_2^{eq} and drop the notation for $a_0^{eq} = 1$.

$$\frac{d\langle r \rangle([R])}{d[R]} = -\frac{a_1^{eq} b_2^{eq}[R]^2 + 2b_2^{eq}[R] - a_1^{eq} b_0^{eq} + b_1^{eq}}{(b_0^{eq} + b_1^{eq}[R] + b_2^{eq}[R]^2)^2} \qquad [196]$$

So for the output curve to be non monotonic, the first derivative should change sign, and therefore have at least one zero. With the use of Decartes rule of sign, we know that the output function is non monotonic, if the following condition is verified.

$$a_1^{eq} b_0^{eq} > b_1^{eq} \qquad [197]$$

If we plug in the rate constants again, we have the following condition.

$$\frac{k_{PXP}}{k_{XPP}} > \frac{k_{SX}}{k_{XS}} + \frac{k_{SR}}{k_{RS}} + \frac{k_{SX}}{k_{XS}}\frac{k_{XL}}{k_{LX}} \qquad [198]$$

We assume that $k_{PXP} = k_{SX} = k_{SR}$ because these rate constants represent the on-rates of the same protein. Furthermore, we posit that the polymerase and the repressor bound at site X do not interact, as this binding site is not immediately adjacent to the promoter of interest. Consequently, we assume that the off-rates of the repressor at binding site X, whether interacting with the polymerase or not, are equal, i.e. $k_{XPP} = k_{XS}$. We further simplify the condition for non-monotonicity in Eq. [199] and note that since all rate constants are positive, this condition cannot be satisfied. Namely, our condition reveals that non-monotonicity at equilibrium is inaccessible because the condition Eq. [198] cannot be satisfied under this biological constraints.

$$0 > \frac{1}{k_{RS}} + \frac{1}{k_{XS}}\frac{k_{XL}}{k_{LX}} \qquad [199]$$

In a 4-state graph, arguments prevent non-monotonicity in a simple activation graph: at equilibrium, non-monotonic curves cannot arise, and even out of equilibrium, non-monotonicity remains unreachable if the on-rates of the polymerase with or without the transcription factor are equal. A similar situation occurs in the looping architecture by repression: due to biological constraints, non-monotonicity is unlikely at equilibrium, even though the output curve's functional form would have *a priori* allowed it.

F.3. Activation. Looping may also prove advantageous in the context of regulatory elements associated with activation units. We consider two different activators A_1 and A_2 binding on two different binding sites and contacting two distinct surfaces of the RNA polymerase (the case of activation by CRP dimers (62)). The binding site of A_2 is considered to be further than the binding site of A_1. Activation through A_1 resembles a simple activation scheme. Nevertheless, for A_2 and the polymerase to interact with each other, the DNA has to bend, which has an extra-energy cost. Once again this cost is balanced by the fact that the polymerase is now very strongly bound to the polymerase. We consider that looping can only be performed between A_2 and the polymerase. This architecture is similar to the one involving two independent activators. The added steps are the looping events and the fact that A_1 can still bind on the looped DNA. The architecture is depicted in the Figure S50(A). We derive the functional form of the output curve departing from the one derived from

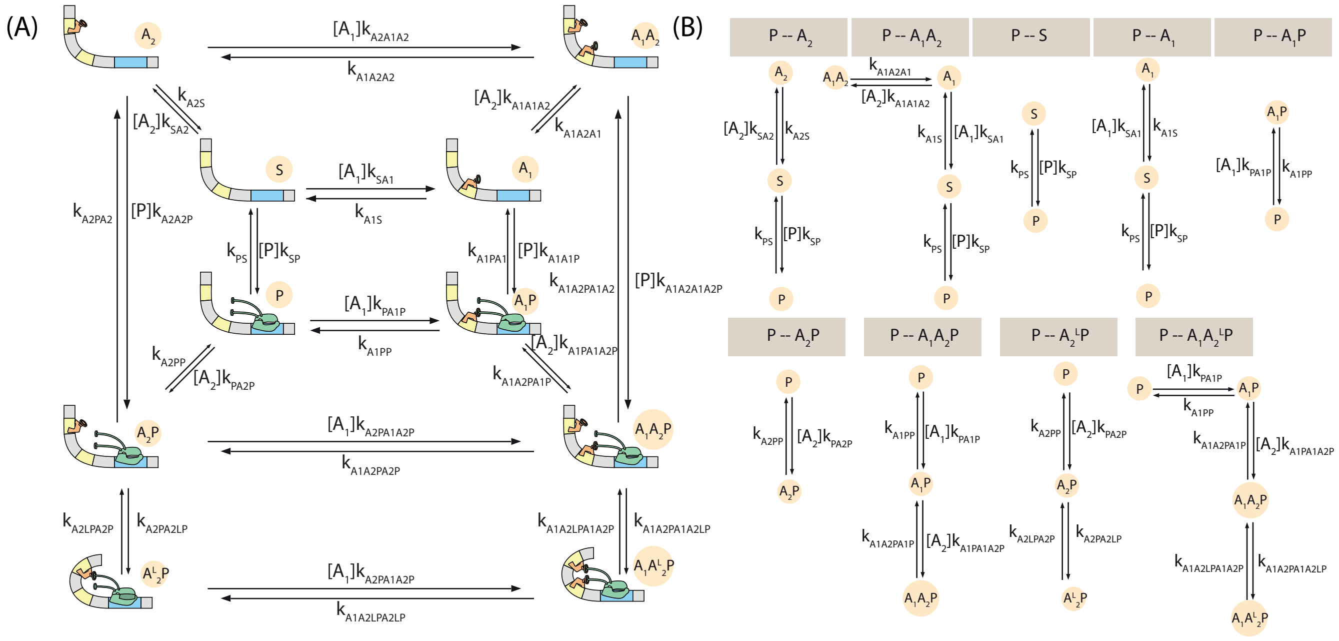

Fig. S50. Graph representing an activation motif with looping of the DNA and characteristic paths the functional form of the output function at equilibrium. (A) The different states and transitions between them for a regulatory unit performing activation and looping. On each arrow is written the rate at which this transition occurs. When a molecule (here RNA polymerase P or activators A_1, A_2) binds, the rate is proportional to the concentration in this molecule. The state S represents the empty DNA, the state P, the DNA with only the RNAP bound, state A_1 and A_2 in which only the activator having its binding site respectively closest or farthest to the RNAP binding site is bound, the repressor bound to the main (repressing). The other non looped states are a combination of the presence and absence of the polymerase or the activators A_1 and A_2. Finally, $A_2^L P$ is the looped state in which A_2 and P are both bound to the DNA and they interact, $A_2^L A_1 P$ is a similar state, but with the presence of A_1, added. (B) Paths from the state P to any other state of the graph (A). These paths play a role into determining the output functional form at equilibrium.

the cube. Indeed, to enumerate the spanning trees of the graph of Figure S50(A), we can enumerate the spanning trees of the cube composed of the states with no looping involved. We then have to complete them with edges from the set of edges $\{A_2P \to A_2^LP, A_2^LP \to A_2P, A_2^LP \to A_1A_2^LP, A_1A_2^LP \to A_2^LP, A_1A_2^LP \to A_1A_2P, A_1A_2P \to A_1A_2^LP\}$, which are not in the mentioned cube, to create the spanning trees of the total graph. There is only one edge dependent on $[A_1]$ ($A_2^LP \to A_1A_2^LP$) and the path $\{A_2^LP \to A_1A_2^LP, A_1A_2^LP \to A_1A_2P\}$ added to any spanning tree of the cube C_{A_1,A_2}, systematically creates a spanning tree of the total graph $G^L_{A_1,A_2}$, because it connects the rest of the nodes to the tree and only adds an incoming edge to a node from the cube, which still respects the structure of a spanning tree. So concerning the dependency on the concentration, the order on $[A_1]$ of the maximally weighted spanning tree(s) is increased by one. The minimally weighed spanning remains the same, because we can also complete any spanning tree from $C_{A_1,A2}$ by the path $\{A_1A_2^LP \to A_2^LP, A_2^LP \to A_2P\}$, of weight of order 0 in both $[A_1]$ and $[A_2]$, applying the same reasoning. To conclude, we can write the following functional form for the output.

$$\langle r \rangle = \frac{\sum\limits_{i+j\leq 7,\ i\leq 5,\ j\leq 4} a_{ij}[A_1]^i[A_2]^j}{\sum\limits_{i+j\leq 7,\ i\leq 5,\ j\leq 4} b_{ij}[A_1]^i[A_2]^j} \qquad [200]$$

This dependency is depicted in Figure S51(B), a dot on the (i,j) space corresponding to the presence of a term proportional to $[A_1]^i[A_2]^j$ in both the numerator and the denominator of the output function. Again, we can explore the new phenotypes of output curves in a 3D space now, in which we can tune both the concentration $[A_1]$ and $[A_2]$. In Figure S51(A), we see that with respect to $[A_1]$ the output function goes from strongly nonmonotonic to monotonic by the simple change of concentration $[A_1]$ when the system is out of equilibrium.

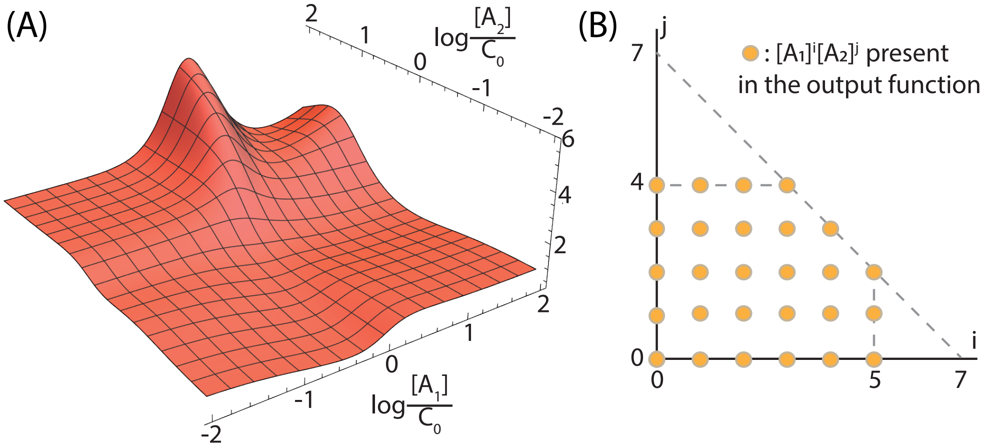

Fig. S51. Example of an output curve $\langle r \rangle$ for activation by looping and graphical representation of the powers of the concentrations of activator present in the numerator and denominator of the output function. (A) Output curve of the graph S50(A) out of equilibrium, defined in Eq. [200]. This is plotted with respect to the concentrations $[A_1]$ and $[A_2]$, in a logarithmic scale. The log is in base 10 and $C_0 = 1nM$. This is plotted for the values :$\{a_{00} = 1, a_{10} = 1, a_{20} = 1, a_{30} = 1, a_{40} = 10, a_{50} = 10, a_{01} = 20, a_{11} = 20, a_{21} = 10, a_{31} = 10, a_{41} = 20, a_{51} = 1, a_{02} = 1, a_{12} = 1, a_{22} = 1, a_{32} = 20, a_{42} = 20, a_{52} = 10, a_{03} = 10, a_{13} = 20, a_{23} = 1, a_{33} = 1, a_{43} = 20, a_{04} = 20, a_{14} = 1, a_{24} = 30, a_{34} = 1\}$ and $\{b_{00} = 1, b_{10} = 10, b_{20} = 1, b_{30} = 10, b_{40} = 1, b_{50} = 10, b_{01} = 10, b_{11} = 20, b_{21} = 20, b_{31} = 20, b_{41} = 20, b_{51} = 30, b_{02} = 10, b_{12} = 1, b_{22} = 30, b_{32} = 1, b_{42} = 1, b_{52} = 1, b_{03} = 1, b_{13} = 1, b_{23} = 1, b_{33} = 10, b_{43} = 10, b_{04} = 10, b_{14} = 1, b_{24} = 1, b_{34} = 1\}$. (B) Powers of $[A_1]^i[A_2]^j$ involved in the numerator and denominator of the output function Eq. [200]. If there is an orange dot in the (i, j) plane, then there is a term proportional to $[A_1]^i[A_2]^j$ in both numerator and denominator of Eq. [200].

At equilibrium, we can use the result stated in Eq. [46], like the repression looping scheme. Choosing the reference state to be P, the paths from P to all the other nodes of the network have at most dependency $[A_1][A_2]$ and at least 0 in $([A_1], [A_2])$ because of the bi-molecular nature of a single step. This is illustrated in Figure S50(B).

Therefore the output at equilibrium has the following shape.

$$\langle r \rangle_{eq} = \frac{a_{00}^{eq} + a_{10}^{eq}[A_1] + a_{01}^{eq}[A_2] + a_{11}^{eq}[A_1][A_2]}{b_{00}^{eq} + b_{10}^{eq}[A_1] + b_{01}^{eq}[A_2] + b_{11}^{eq}[A_1][A_2]} \qquad [201]$$

References

1. JA Owen, JM Horowitz, Size limits the sensitivity of kinetic schemes. *Nat. Commun.* **14**, 1280 (2023).
2. R Grah, B Zoller, G Tkačik, Nonequilibrium models of optimal enhancer function. *Proc. Natl. Acad. Sci. U.S.A.* **117**, 31614–31622 (2020).
3. M Morrison, M Razo-Mejia, R Phillips, Reconciling kinetic and thermodynamic models of bacterial transcription. *PLoS Comput. Biol.* **17**, e1008572 (2021).
4. TL Forcier, et al., Measuring *cis*-regulatory energetics in living cells using allelic manifolds. *eLife* **7**, e40618 (2018).
5. H Qian, Phosphorylation energy hypothesis: open chemical systems and their biological functions. *Annu. Rev. Phys. Chem.* **58**, 113–142 (2007).
6. C Hueschen, R Phillips, *The Restless Cell: Continuum Theories of Living Matter.* (Princeton University Press), (2024).
7. JJ Hopfield, Kinetic proofreading: a new mechanism for reducing errors in biosynthetic processes requiring high specificity. *Proc. Natl. Acad. Sci.* **71**, 4135–4139 (1974).
8. J Ninio, Kinetic amplification of enzyme discrimination. *Biochimie* **57**, 587–595 (1975).
9. A Murugan, DA Huse, S Leibler, Speed, dissipation, and error in kinetic proofreading. *Proc. Natl. Acad. Sci.* **109**, 12034–12039 (2012).
10. G Lan, P Sartori, S Neumann, V Sourjik, Y Tu, The energy–speed–accuracy trade-off in sensory adaptation. *Nat. physics* **8**, 422–428 (2012).
11. AC Barato, D Hartich, U Seifert, Efficiency of cellular information processing. *New J. Phys.* **16**, 103024 (2014).
12. D Hartich, AC Barato, U Seifert, Nonequilibrium sensing and its analogy to kinetic proofreading. *New J. Phys.* **17**, 055026 (2015).
13. A Goldbeter, DE Koshland Jr, An amplified sensitivity arising from covalent modification in biological systems. *Proc. Natl. Acad. Sci. U.S.A.* **78**, 6840–6844 (1981).
14. P De Los Rios, A Barducci, Hsp70 chaperones are non-equilibrium machines that achieve ultra-affinity by energy consumption. *Elife* **3**, e02218 (2014).
15. H Qian, Thermodynamic and kinetic analysis of sensitivity amplification in biological signal transduction. *Biophys. chemistry* **105**, 585–593 (2003).
16. CC Govern, PR ten Wolde, Energy dissipation and noise correlations in biochemical sensing. *Phys. review letters* **113**, 258102 (2014).

17. CC Govern, PR Ten Wolde, Optimal resource allocation in cellular sensing systems. *Proc. Natl. Acad. Sci.* **111**, 17486–17491 (2014).
18. AC Barato, U Seifert, Coherence of biochemical oscillations is bounded by driving force and network topology. *Phys. Rev. E* **95**, 062409 (2017).
19. J Howard, *Mechanics of Motor Proteins and the Cytoskeleton.* (Sinauer Associates, Publishers), (2001).
20. FM Gartner, IR Graf, E Frey, The time complexity of self-assembly. *Proc. Natl. Acad. Sci.* **119**, e2116373119 (2022).
21. R Ravasio, et al., A minimal scenario for the origin of non-equilibrium order. *arXiv preprint arXiv:2405.10911* (2024).
22. M Nguyen, S Vaikuntanathan, Design principles for nonequilibrium self-assembly. *Proc. Natl. Acad. Sci.* **113**, 14231–14236 (2016).
23. CG Evans, J O'Brien, E Winfree, A Murugan, Pattern recognition in the nucleation kinetics of non-equilibrium self-assembly. *Nature* **625**, 500–507 (2024).
24. TL Hill, *Free energy transduction and biochemical cycle kinetics.* (Courier), (2013).
25. VH Tierrafría, et al., RegulonDB 11.0: Comprehensive high-throughput datasets on transcriptional regulation in *Escherichia coli* K-12. *Microb. Genom.* **8** (2022).
26. WT Ireland, et al., Deciphering the regulatory genome of *Escherichia coli*, one hundred promoters at a time. *eLife* **9**, e55308 (2020).
27. M Rydenfelt, HG Garcia, RS Cox III, R Phillips, The influence of promoter architectures and regulatory motifs on gene expression in *Escherichia coli*. *PLoS One* **9**, e114347 (2014).
28. NC Lammers, AI Flamholz, HG Garcia, Competing constraints shape the nonequilibrium limits of cellular decision-making. *Proc. Natl. Acad. Sci. U.S.A.* **120**, e2211203120 (2023).
29. R Shelansky, H Boeger, Nucleosomal proofreading of activator–promoter interactions. *Proc. Natl. Acad. Sci. U.S.A.* **117**, 2456–2461 (2020).
30. R Shelansky, et al., A telltale sign of irreversibility in transcriptional regulation. *bioRxiv* pp. 2022–06 (2022).
31. LA Mirny, Nucleosome-mediated cooperativity between transcription factors. *Proc. Natl. Acad. Sci. U.S.A.* **107**, 22534–22539 (2010).
32. SV Keränen, A Villahoz-Baleta, AE Bruno, MS Halfon, REDfly: an integrated knowledgebase for insect regulatory genomics. *Insects* **13**, 618 (2022).
33. TC Kaufman, A short history and description of *Drosophila melanogaster* classical genetics: Chromosome aberrations, forward genetic screens, and the nature of mutations. *Genetics* **206**, 665–689 (2017).
34. L Li, Z Wunderlich, An enhancer's length and composition are shaped by its regulatory task. *Front. genetics* **8**, 267395 (2017).
35. Z Wunderlich, LA Mirny, Different gene regulation strategies revealed by analysis of binding motifs. *Trends genetics* **25**, 434–440 (2009).
36. L Bintu, et al., Transcriptional regulation by the numbers: models. *Curr. Opin. Genet. Dev.* **15**, 116–124 (2005).
37. HC Nelson, RT Sauer, Lambda repressor mutations that increase the affinity and specificity of operator binding. *Cell* **42**, 549–558 (1985).
38. R Milo, P Jorgensen, U Moran, G Weber, M Springer, Bionumbers—the database of key numbers in molecular and cell biology. *Nucleic Acids Res.* **38**, D750–D753 (2010).
39. AD Riggs, S Bourgeois, M Cohn, The *lac* represser-operator interaction: III. Kinetic studies. *J. Mol. Biol.* **53**, 401–417 (1970).
40. E Marklund, et al., Sequence specificity in DNA binding is mainly governed by association. *Science* **375**, 442–445 (2022).
41. P Hammar, et al., Direct measurement of transcription factor dissociation excludes a simple operator occupancy model for gene regulation. *Nat. Genet.* **46**, 405–408 (2014).
42. R Milo, R Phillips, *Cell biology by the numbers.* (Garland Science), (2015).
43. J Elf, GW Li, XS Xie, Probing transcription factor dynamics at the single-molecule level in a living cell. *Science* **316**, 1191–1194 (2007).
44. AP Singh, et al., 3D protein dynamics in the cell nucleus. *Biophys. J.* **112**, 133–142 (2017).
45. M Razo-Mejia, et al., Tuning transcriptional regulation through signaling: a predictive theory of allosteric induction. *Cell Syst.* **6**, 456–469 (2018).
46. L Xu, et al., Average gene length is highly conserved in prokaryotes and eukaryotes and diverges only between the two kingdoms. *Mol. Biol. Evol.* **23**, 1107–1108 (2006).
47. AJ Meyer, TH Segall-Shapiro, E Glassey, J Zhang, CA Voigt, *Escherichia coli* "marionette" strains with 12 highly optimized small-molecule sensors. *Nat. Chem. Biol.* **15**, 196–204 (2019).
48. O Shoval, et al., Fold-change detection and scalar symmetry of sensory input fields. *Proc. Natl. Acad. Sci. U.S.A.* **107**, 15995–16000 (2010).
49. RK Zia, B Schmittmann, Probability currents as principal characteristics in the statistical mechanics of non-equilibrium steady states. *J. Stat. Mech.* **2007**, P07012 (2007).
50. J Schnakenberg, Network theory of microscopic and macroscopic behavior of master equation systems. *Rev. Mod. Phys.* **48**, 571 (1976).
51. H Qian, Open-system nonequilibrium steady state: statistical thermodynamics, fluctuations, and chemical oscillations (2006).

52. I Mirzaev, J Gunawardena, Laplacian dynamics on general graphs. *Bull. Math. Biol.* **75**, 2118–2149 (2013).
53. J Estrada, F Wong, A DePace, J Gunawardena, Information integration and energy expenditure in gene regulation. *Cell* **166**, 234–244 (2016).
54. KM Nam, R Martinez-Corral, J Gunawardena, The linear framework: using graph theory to reveal the algebra and thermodynamics of biomolecular systems. *Interface Focus.* **12**, 20220013 (2022).
55. D Curtiss, Recent extentions of descartes' rule of signs. *Ann. Math.* pp. 251–278 (1918).
56. Y Tu, The nonequilibrium mechanism for ultrasensitivity in a biological switch: Sensing by Maxwell's demons. *Proc. Natl. Acad. Sci. U.S.A.* **105**, 11737–11741 (2008).
57. H Tran, et al., Precision in a rush: Trade-offs between reproducibility and steepness of the *hunchback* expression pattern. *PLoS Comput. Biol.* **14**, e1006513 (2018).
58. O Bernardi, On the spanning trees of the hypercube and other products of graphs. *arXiv preprint arXiv:1207.0896* (2012).
59. R Stanley, *Enumerative Combinatorics: Volume 2.* (Cambridge University Press), (1999).
60. E Eck, et al., Quantitative dissection of transcription in development yields evidence for transcription-factor-driven chromatin accessibility. *eLife* **9**, e56429 (2020).
61. SM Law, GR Bellomy, PJ Schlax, MT Record, *In vivo* thermodynamic analysis of repression with and without looping in *lac* constructs: Estimates of free and local *lac* repressor concentrations and of physical properties of a region of supercoiled plasmid DNA *in vivo*. *J. Mol. Biol.* **230**, 161–173 (1993).
62. JK Joung, LU Le, A Hochschild, Synergistic activation of transcription by *Escherichia coli* cAMP receptor protein. *Proc. Natl. Acad. Sci. U.S.A.* **90**, 3083–3087 (1993).
63. M Lewis, The *lac* repressor. *Comptes Rendus Biol.* **328**, 521–548 (2005) Retour sur l'operon lac.
64. JQ Boedicker, HG Garcia, R Phillips, Theoretical and experimental dissection of DNA loop-mediated repression. *Phys. Rev. Lett.* **110**, 018101 (2013).
65. JL Lebowitz, H Spohn, A Gallavotti–Cohen-type symmetry in the large deviation functional for stochastic dynamics. *J. Stat. Phys.* **95**, 333–365 (1999).
66. L Bintu, et al., Transcriptional regulation by the numbers: applications. *Curr. Opin. Genet. Dev.* **15**, 125–135 (2005).
67. YJ Chen, S Johnson, P Mulligan, AJ Spakowitz, R Phillips, Modulation of DNA loop lifetimes by the free energy of loop formation. *Proc. Natl. Acad. Sci. U.S.A.* **111**, 17396–17401 (2014).
